# Supplementary material for: Synaptic and intrinsic membrane defects disrupt early neural network dynamics in Down syndrome
Source: Nat Commun. 2026 Jan 22;17:1287. doi: 10.1038/s41467-025-68048-x (PMC12868644; doi:10.1038/s41467-025-68048-x)
Supplement: Supplementary file 1 — Supplementary Information [file 41467_2025_68048_MOESM1_ESM.pdf]

Supplemental Information

Synaptic and intrinsic membrane defects disrupt early neural network dynamics in Down syndrome

Saad B. Hannan[1,2*], Ivan Alić[3,4], Aoife Murray[3], Joonhong Kwon[5], Martin Mortensen[1], Hyo Jung Kang[5], Ante Plećaš[4], Pollyanna A. Goh[3], Niamh L O'Brien[3], Richard Naud[6], Dean Nižetić[3*] and Trevor G. Smart[1*]

*Corresponding authors: t.smart@ucl.ac.uk and saadhannan@fas.harvard.edu for all queries, and to d.nizetic@qmul.ac.uk for queries regarding iPSC models

The PDF file includes:

Figures. S1 to S9
Table S1
Supplementary data 1 to 6

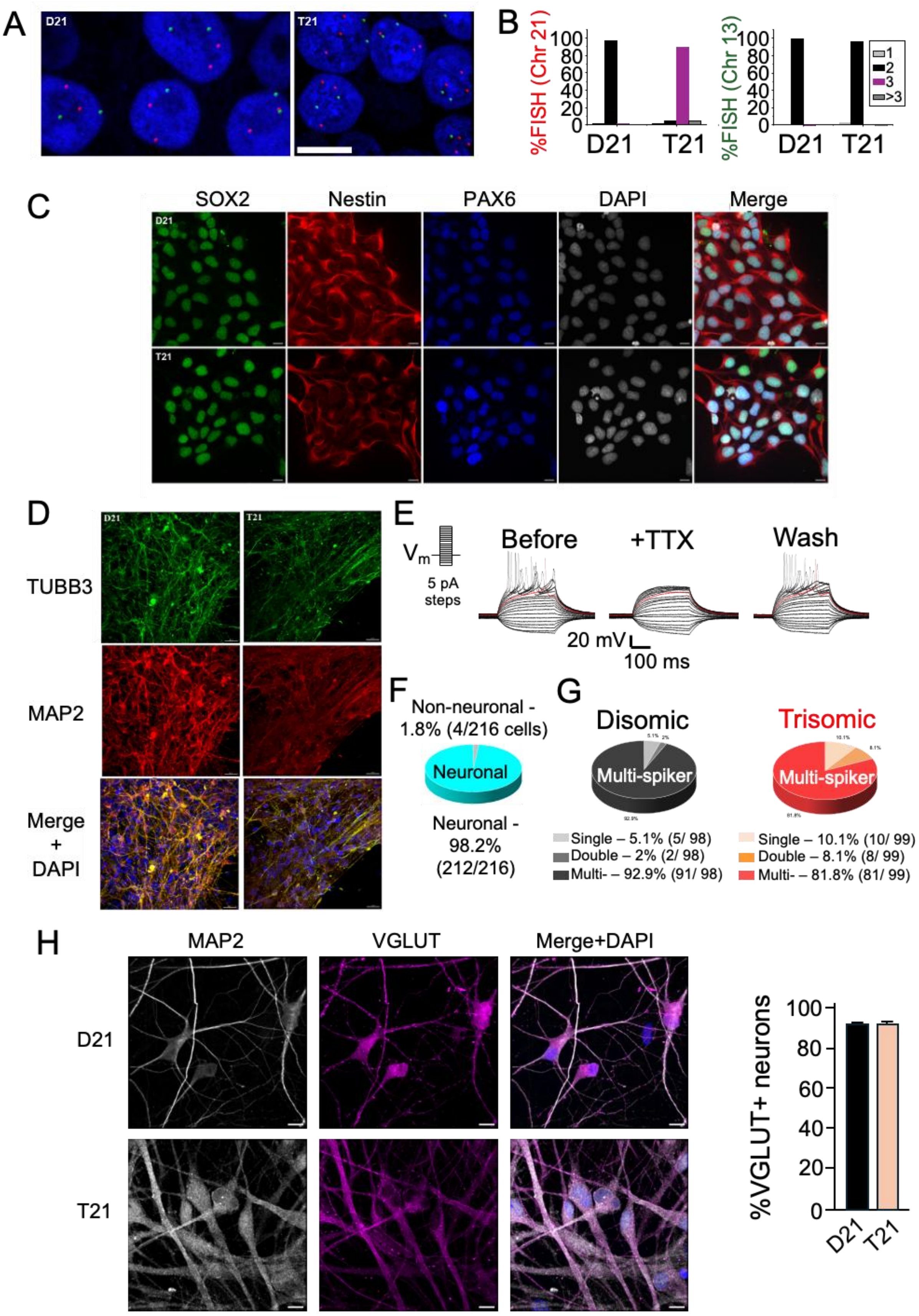
A
D21
T21
B
%FISH (Chr 21)
%FISH (Chr 13)
D21 T21
1
2
3
>3
C
SOX2 Nestin PAX6 DAPI Merge
D21
T21
D
TUBB3
MAP2
Merge + DAPI
E
Before +TTX Wash
5 pA steps
20 mV
100 ms
F
Non-neuronal - 1.8% (4/216 cells)
Neuronal
Neuronal - 98.2% (212/216)
G
Disomic
Trisomic
Multi-spiker
Single – 5.1% (5/ 98)
Double – 2% (2/ 98)
Multi- – 92.9% (91/ 98)
Single – 10.1% (10/ 99)
Double – 8.1% (8/ 99)
Multi- – 81.8% (81/ 99)
H
MAP2 VGLUT Merge+DAPI
D21
T21
%VGLUT+ neurons

Figure S1. – Validation of cell lines and neural differentiation

A, Confocal images of disomic (D21) and trisomic (T21) neural stem cell nuclei (DAPI; blue) stained for chromosome 21 (red) using florescence in-situ hybridization. Chromosome 13 (green) staining has been used as a control. Scale bars - 10 µm. ***B,*** Quantification for chromosome 21 (left) and 13 (right) numbers in D21 and T21 cells showing presence of three copies of chromosome 21 in T21 cells. ***C,*** Representative images of isogenic disomic (top row) and trisomic (bottom row) cells stained with neural stem cell specific markers SOX2 (green), Nestin (red), PAX6 (blue) and counterstained with DAPI (gray). Scale bars - 10 µm. ***D,*** Representative images of mature isogenic disomic (D21, left column) and trisomic (T21, right column) neurons stained with pan-neuronal markers TUBB3 (green) MAP2 (red) and counterstained with DAPI (blue). Scale bars - 20 µm. ***E,*** Action potentials elicited by injection of constant current steps in human Down syndrome neurons. The current at which the neuron fires the first action potential (rheobase) has been depicted in red. Application of tetrodotoxin (TTX; 0.5 µM) abolishes action potentials which can be recovered upon wash off. ***F,*** Percentage of cells that fire stereotypical action potentials in whole cell current clamp recordings. 114 disomic and 102 trisomic cells from seven batches were recorded in current clamp and overall >98% of cells characterized using single cell electrophysiology were neurons based on action potential firing ability. Note that single cell electrophysiology relies on selecting cells using transmitted light microscopy factoring in morphology, quality of membrane and cellular health. ***G,*** Proportion of disomic and trisomic neurons that fired a maximum of one spike, two spikes or more than two spikes (multi-spiker). After establishing current clamp and recording basal spiking, a step current injection protocol was initiated allowing for saturation of spike input-output curve enabling the determination of maximum spiking properties. ***H,*** Confocal images of D21 and T21 neurons expressing MAP2 and glutamatergic marker VGLUT1 along with quantification of percentages of VGLUT1-expressing MAP2 positive cells. Note - >93% cells expressed this glutamatergic marker. Scale bars - 10 µm

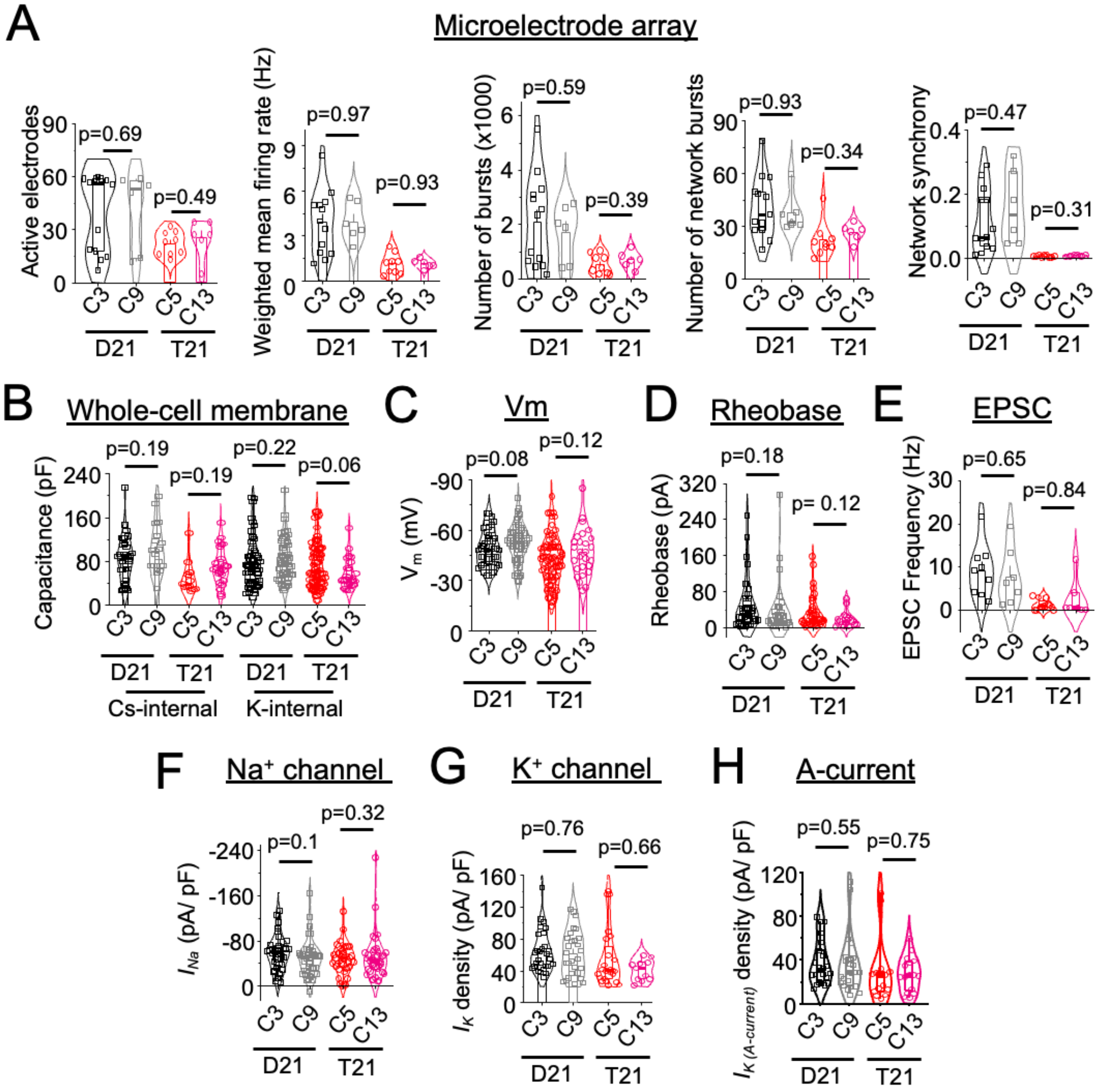

Figure S2. Absence of overt inter-clonal functional variations within disomic (C3/ C9) and trisomic (C5/ C13) iPSC lines

A, Number of active electrodes, weighted mean firing rate, number of electrode bursts, number of network bursts and synchrony index of C3 and C9 disomic lines along with C5 and C13 trisomic lines in microelectrode array recordings of weeks 9-10 iPSC-derived neurons. ***B,*** Whole cell membrane capacitance of disomic and trisomic clones recorded using a Cs- or K-based internal solution. ***C-H,*** Resting membrane potential (V_m) *(C)*, rheobase for spike firing *(D)*, frequency of excitatory postsynaptic currents (EPSCs) in a zero Mg^{2+} bath solution *(E)*, Na^+ channel current density using a depolarizing single step protocol *(F)*, steady-state K^+ channel current density at a 90 mV step of I-V curve *(G)*, and A-type K^+ channel current density *(H)* of C3 and C9 disomic lines along with C5 and C13 trisomic lines. n = 6-13 wells in *(A)*, n = 7-87 cells in *(C-H)*; two tailed-unpaired t-test or Mann-Whitney test between disomic or trisomic clones.

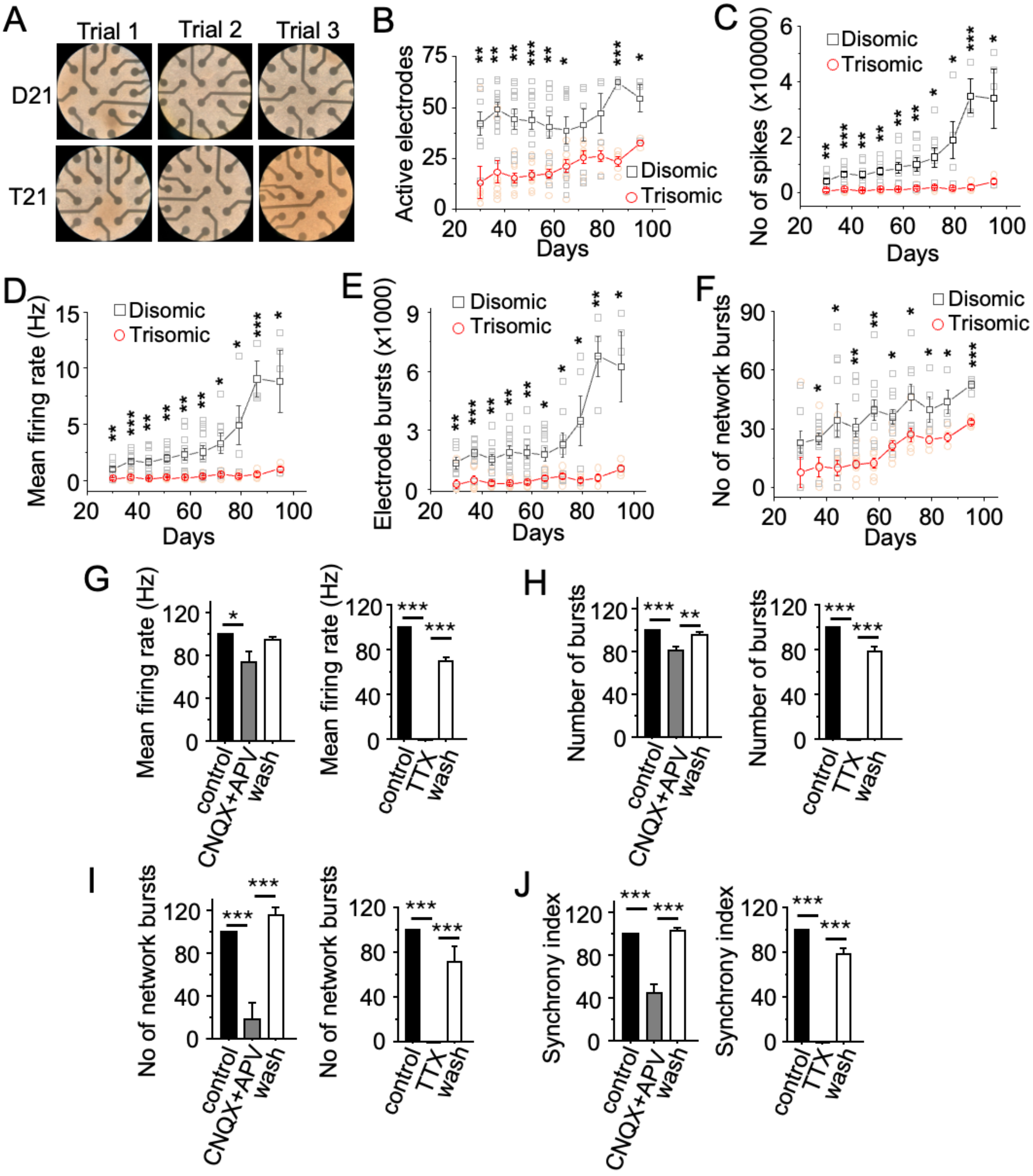

Figure S3. Neural network defects due to trisomy of chromosome 21

A, Transmitted light images of microelectrode array recording electrodes with cells growing on top of them at 14 weeks from three different disomic (D21; top row) and trisomic (T21; bottom row) trial wells. Reduced activity of trisomic cells is not due to absence of cells in contact with recording electrodes. ***B,*** Number of active electrodes in 64-electrode wells during development. Trisomic cells consistently have fewer active electrodes suggesting reduced spiking activity in these networks. ***C,*** Number of total

spikes recorded across development. The number of spikes rises rapidly as disomic cells mature but trisomic cells lag behind at all ages studied. ***D,*** Mean firing rate of disomic and trisomic cells across development and here too trisomic cells lag behind consistently. ***E,*** Number of electrode bursts across development showing deficits in bursting properties of trisomic neurons. ***F,*** Number of network bursts across development showing reduced network bursting of trisomic neurons. ***G-J,*** Normalised mean spike firing rate *(G),* number of electrode bursts *(H)*, number of network bursts *(I)* and synchrony index *(J)* of iPSC-derived disomic and trisomic neurons in presence of the AMPA receptor antagonist CNQX (10 μM) and the NMDA receptor antagonist APV (25 μM) or the Na^{+} channel blocker tetrodotoxin (0.5 μM; TTX). Data has been normalised to basal recordings immediately prior to the application of the pharmacological agents. Note a silencing of spiking and bursting in TTX and reduction of single electrode and network bursts in CNQX and APV confirming that glutamatergic synaptic and intrinsic membrane properties determine spiking and network dynamics in these cortical networks. Results in *A-F* are from five different differentiations of two different lines and at least four wells per time point. In *G-J*, n = 6-8 wells. *P<0.05, **P<0.01. ***P<0.0001, Mann-Whitney test, two-tailed unpaired t-test between or one-way ANOVA.

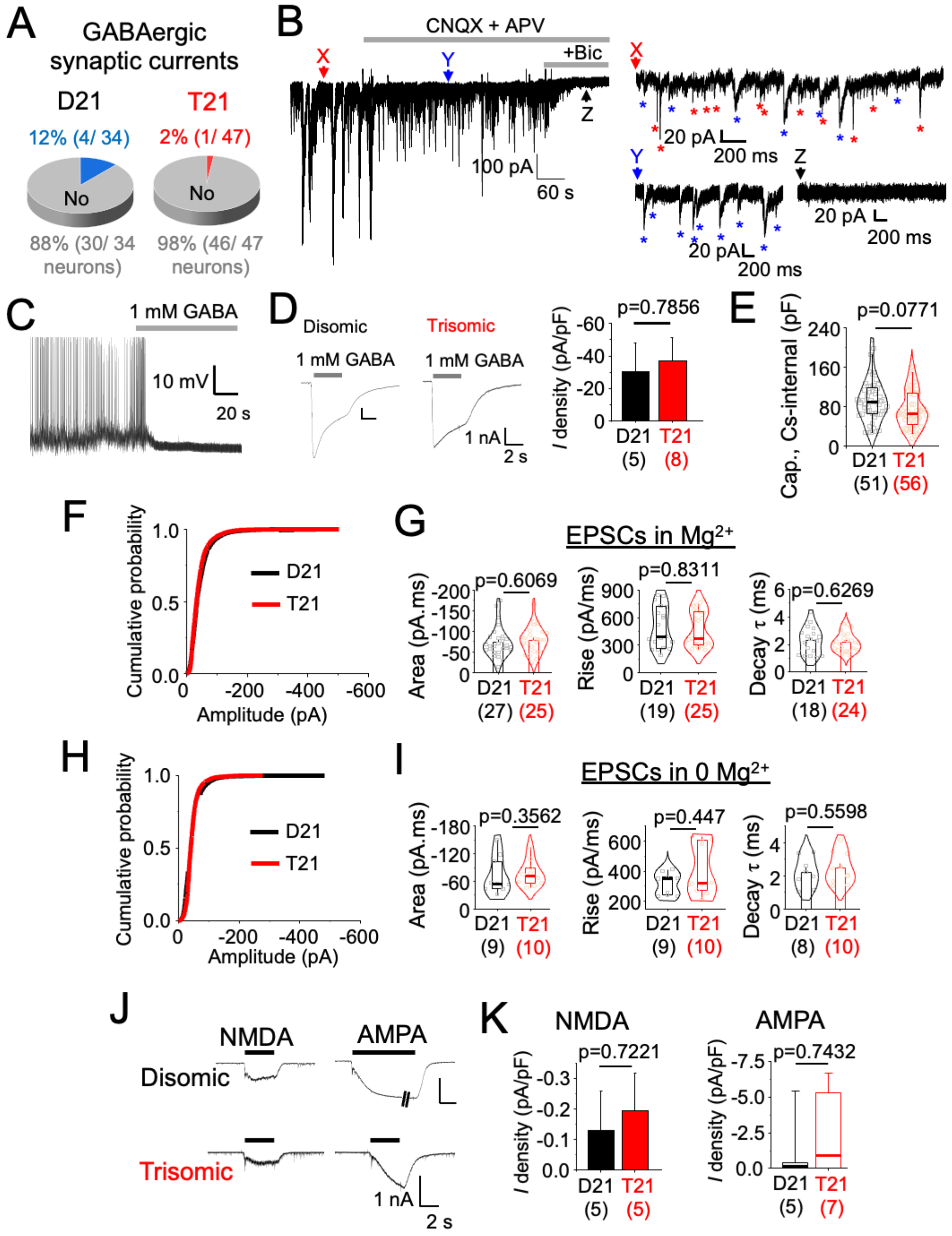

Figure S4. GABAergic postsynaptic currents, EPSC kinetics and AMPA and NMDA whole cell currents

A, Pie chart showing the fraction of cells that had GABAergic inhibitory postsynaptic currents (IPSCs) in cortical glutamatergic iPSC neurons. A limited number of cells (12% disomic (D21) and 2% trisomic (T21)) cells from only one differentiation out of eight received GABAergic inputs. The results from this differentiation have not been incorporated in the analysis. ***B,*** An example trace showing GABAergic postsynaptic currents of an excluded disomic cell. When present, GABAergic synaptic currents were identified by their slower kinetics (blue asterisk) compared to excitatory postsynaptic

currents (red asterisk) in addition to application of the $GABA_A$ receptor antagonist bicuculline (25 µm; +Bic). Section X (red arrow) in the absence antagonists contains a mix of excitatory and inhibitory postsynaptic currents. In Y (blue arrow), application of AMPA and NMDA receptor antagonists CNQX and APV abolishes excitatory events leaving GABAergic events. In Z (black arrow), bicuculline abolished inhibitory events confirming their GABAergic identity. ***C,*** Application of 1 mM GABA blocks spontaneous action potentials in an iPSC-derived disomic neuron showing the presence of functional $GABA_A$ receptor responses. This suggests that the absence of inhibitory postsynaptic currents in these cells are not due to the absence of $GABA_A$ receptors. ***D,*** Representative 1 mM GABA-activated currents and current densities of disomic and trisomic cells. ***E,*** Whole cell capacitance (Cap.), calculated from the area under a -10 mV step capacity discharge curve, shows a trend towards reduced sizes (but not statistically significant) of trisomic (T21) cells compared to their disomic (D21) counterparts. ***F,*** Cumulative probability distributions of excitatory postsynaptic current (EPSC) amplitudes of D21 and T21 cells in Mg^{2+} containing saline solution. n = 12285-38701 events. ***G,*** Unchanged charge transfer, rate of rise and decay times of EPSCs recorded in Mg^{2+} containing saline solution. ***H,*** Cumulative probability distributions of EPSC amplitudes of D21 and T21 cells in saline devoid of Mg^{2+}. n = 5176-23795 events. ***I,*** Similarly, EPSC kinetics of charge transfer, rate of rise and decay times are also unchanged in saline devoid of Mg^{2+}. ***J,*** NMDA- and AMPA-activated currents of disomic (D21) and trisomic cells (T21). NMDA (50 µM) was applied with the co-agonist glycine (10 µM) and AMPA (10 µM) was co-applied with cyclothiazide (50 µM) to prevent desensitization. Cells were held at a potential of -70 mV. ***K,*** Bar charts and box plots showing unchanged NMDA and AMPA current densities in disomic and trisomic iPSC-derived cortical glutamatergic neurons. n numbers shown in brackets under genotypes. n = 5-56 cells; two-tailed unpaired t-test and Mann-Whitney test

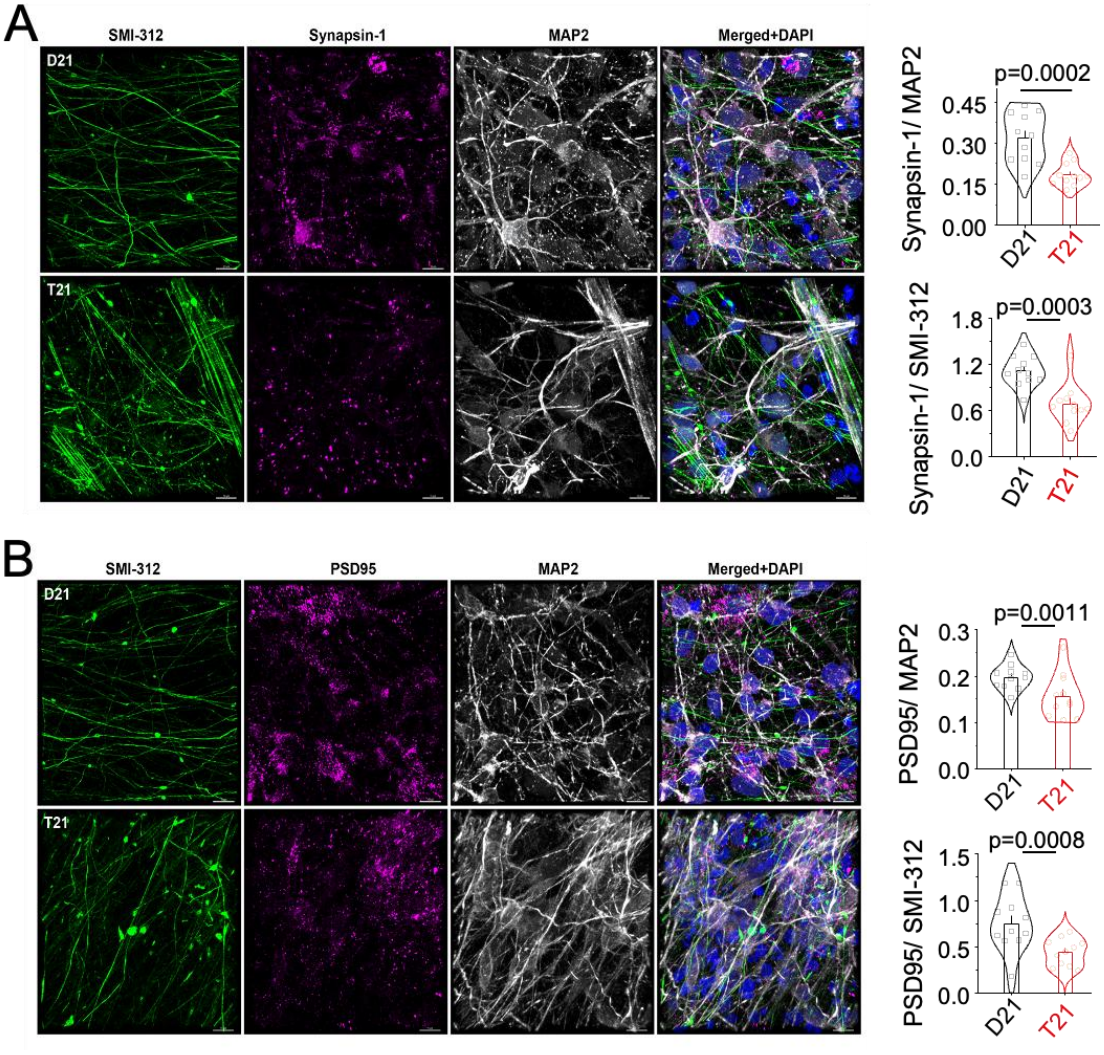

Figure S5. Reduction of number of synapses in trisomy 21 neurons.

*A, Left, r*epresentative images of mature isogenic neurons at sixty days *in vitro* stained with axon-specific marker (SMI-312; green), pre-synaptic marker synapsin-1 (magenta), dendrite-specific marker MAP2 (grey) and counterstained with DAPI (blue). *Right,* analysis showed significantly higher Synapsin-1 expression in both disomic (D21) dendrites and axons compared to trisomic (T21) cells. *B, Left,* Representative images of mature isogenic neurons at sixty days *in vitro* stained with axon-specific marker (SMI-312; green), post-synaptic marker PSD95 (magenta), dendrite-specific marker MAP2 (grey) and counterstained with DAPI (blue). *Right,* analysis showed significantly higher PSD95 expression in both D21 dendrites and axons compared to T21. Scale bars - 10 µm. Graphs represent means ± SEM. n = 10-11 3D image stacks. two-tailed unpaired t-test.

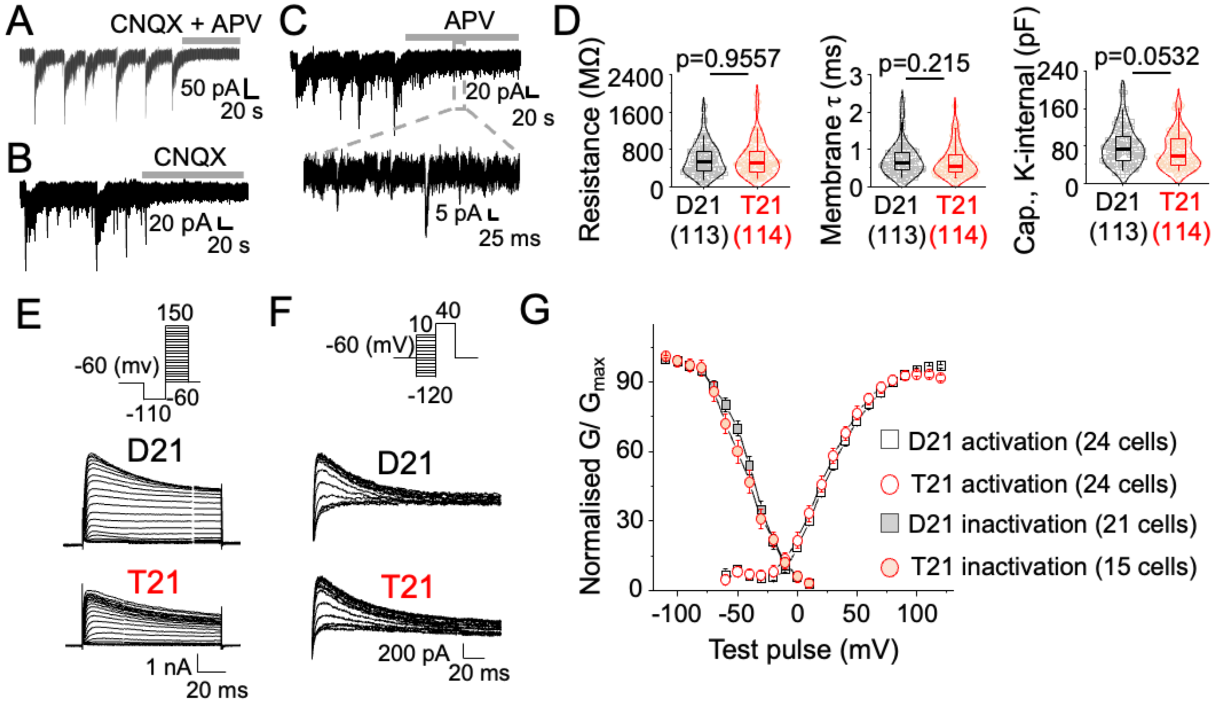

Figure S6. Pharmacological properties of 0 Mg^{2+} bursts, passive membrane properties and A-current activation and inactivation

A, In 0 Mg^{2+} saline, blocking AMPA and NMDA receptors with CNQX and APV abolishes bursts and excitatory postsynaptic currents (EPSCs) in our iPSC-derived cortical glutamatergic neurons. The example shown here is that of a disomic cell. Trisomic cells show similar block of bursting by CNQX and APV. *B,* Blocking AMPA receptors alone with CNQX also abolishes bursts and EPSCs suggesting the importance of AMPA receptor activity and EPSCs for bursting. *C,* Blocking NMDA receptors alone using APV can also abolish bursts but does not block EPSCs (inset) confirming NMDA receptor dependence of bursting. *D,* Input resistance and membrane time constant measured during current clamp recordings do not change between disomic (D21) and trisomic (T21) cells but the capacitance measured from the area under the curve using the same internal solution in response to a -10 mV hyperpolarising step trends towards smaller sized trisomic cells. *E,* Representative A-type K^+ channel currents recorded from disomic (D21) and trisomic (T21) neurons along with the protocol for activating these currents from a hyperpolarised potential (-120 mV) to more depolarized voltages. *F,* Representative A-type K^+ channel inactivation currents along with the step protocol. *G,* Normalized voltage-dependence of activation and inactivation profiles of A-type K^+ channel conductance does not change between disomic and trisomic neurons. N numbers of cells in brackets; Mann-Whitney test.

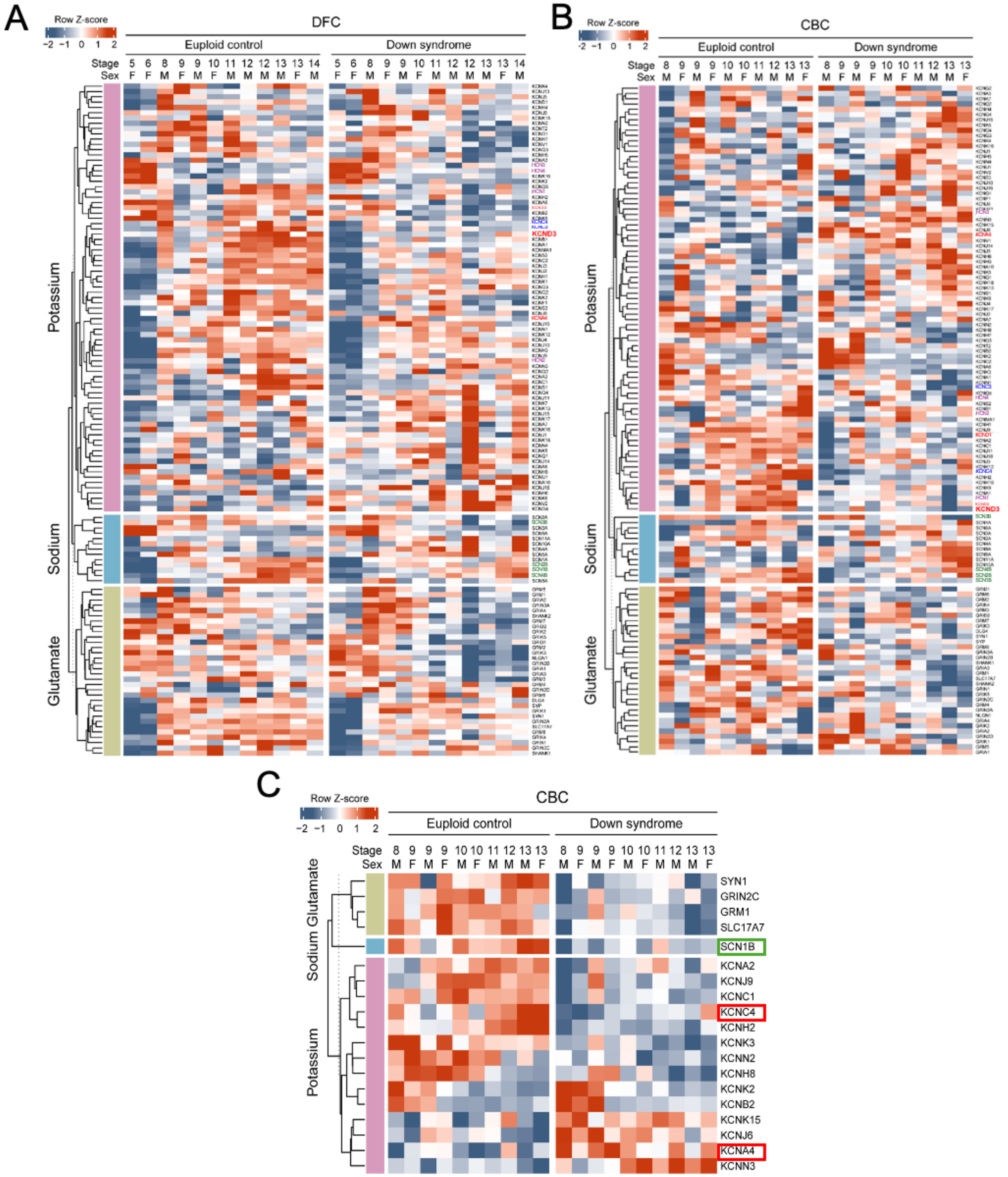

Figure S7. Expression of K⁺, Na⁺ and HCN channels and glutamatergic synapse genes in the dorsolateral prefrontal cortex and cerebellar cortex at various stages of life from human brains

A-B, Heatmaps showing expression of potassium channel-related and hyperpolarization-activated cyclic nucleotide-gated channel-related (purple), sodium channel-related (blue)

and glutamate-related (green) genes in the dorsolateral prefrontal cortex (DFC) (A) or cerebellar cortex (CBC) (B) of euploid controls and Down syndrome brains. Note that HCN channels have been included in the list of K^+ channels. TEA-insensitive A-type K^+ channels have been highlighted with red text, whereas TEA-sensitive A-type K^+ channel genes have been depicted in blue text, HCN channel genes are in purple text and Na^+ channel β-subunit genes that affect channel kinetics have been depicted with green text. The z score indicates intensity of expression (red – high, blue – low). *C,* Heatmap showing changes of expression levels (|Fold Change| > 1.3 and P<0.01) of potassium channel-related (purple), sodium channel-related (blue) and glutamate-related (green) genes in CBC in euploid controls and Down syndrome brains after birth. Changes to TEA-sensitive A-type K^+ channels have been highlighted with red boxes (*KCNC4* has mixed sensitivity depending on oligomerization states) and a Na^+ channel β-subunit gene that affects channel kinetics has been depicted with a green box. Note that *KCND3* is not a differentially expressed gene in this region. Table S1 contains a description of the stages of life.

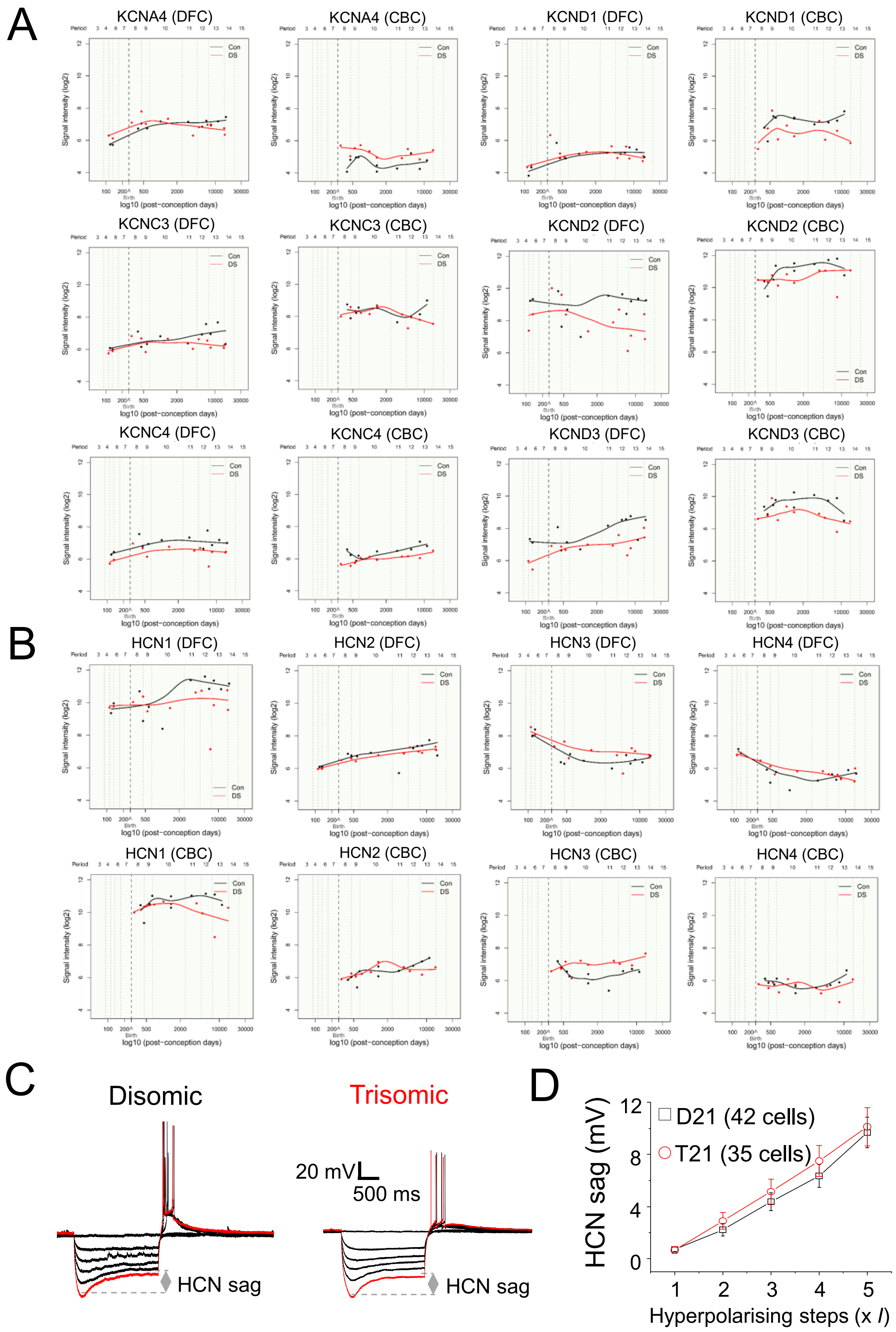
A
KCNA4 (DFC)
KCNA4 (CBC)
KCND1 (DFC)
KCND1 (CBC)
KCNC3 (DFC)
KCNC3 (CBC)
KCND2 (DFC)
KCND2 (CBC)
KCNC4 (DFC)
KCNC4 (CBC)
KCND3 (DFC)
KCND3 (CBC)
B
HCN1 (DFC)
HCN2 (DFC)
HCN3 (DFC)
HCN4 (DFC)
HCN1 (CBC)
HCN2 (CBC)
HCN3 (CBC)
HCN4 (CBC)
Signal intensity (log2)
log10 (post-conception days)
Con
DS
C
Disomic
Trisomic
20 mV
500 ms
HCN sag
HCN sag
D
D21 (42 cells)
T21 (35 cells)
HCN sag (mV)
Hyperpolarising steps (x I)

Figure S8. A-current and HCN gene expression in dorsolateral prefrontal cortex and cerebellar cortex in Down syndrome

A**,* Gene expression patterns of *KCNA4*, *KCNC3*, *KCNC4*, *KCND1*, *KCND2* and *KCND3* are visualized using line graphs in the dorsolateral prefrontal cortex (DFC) and cerebellar cortex (CBC). Note that in the DFC, at early stages of development only expression of *KCND3* and *KCNC4* are significant. Table S1 contains a description of these stages. Stages 5 to 8 correspond to periods from mid-fetal development (16 weeks post-conception) to infancy (up to 6 months after birth). ***B, Gene expression patterns of *HCN1*, *HCN2*, *HCN3* and *HCN4* visualized using line graphs in DFC and CBC. ***C,*** Example HCN sags elicited upon hyperpolarisation of membrane voltage. Note rebound spikes upon release from hyperpolarization consistent with HCN activity. Hyperpolarising steps of currents were carefully injected in suitable equal increments such that the maximum hyperpolarising step yielded membrane potentials close to -150 mV or the cell underwent di-electric breakdown in which case the membrane voltage measures in the sweeps immediately prior to di-electric breakdown were analyzed. ***D,*** HCN sags do not change in trisomic (T21) cells compared to their disomic (D21) counterparts across a range of hyperpolarisations. n = 35-42 cells for HCN analysis, two-tailed unpaired t-test or Mann-Whitney test. Table S1 contains a description of the stages of life.

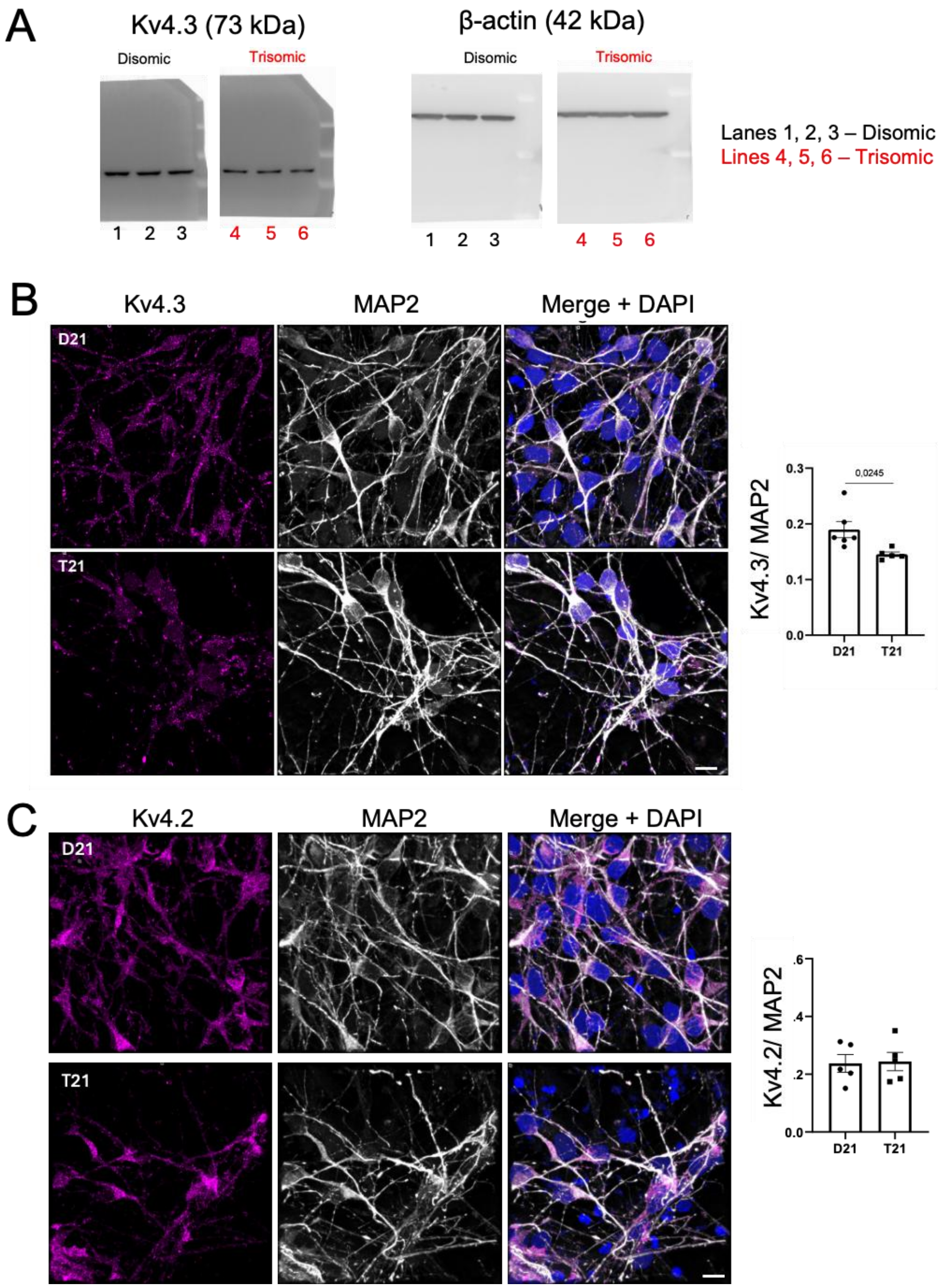

Figure S9. Reduced expression of Kv4.3 but not Kv4.2 in Down syndrome neurons

A, Full Western blots of results in Figure 7G showing the expression levels of Kv4.3 and β-actin in the same lanes. ***B,*** Representative images and quantification of Kv4.3 expression in disomic (D21) and trisomic (T21) isogenic iPSC-derived neurons at 60 days *in vitro* (DIV) showing reduced expression of Kv4.3 due to trisomy 21. ***C,*** Representative images and quantification of Kv4.2 expression in D21 and T21 neurons showing

unchanged expression of a TEA-insensitive A-type K^+ channel Kv4.2. n = 5-6 3D stacks. unpaired t-test. Scale bars - 10 μm.

Table S1. Periods of human development and adulthood

Stage	Age	Description
1	4 ≤ Age < 8 postconceptional weeks	Embryonic development
2	8 ≤ Age < 10 postconceptional weeks	Early fetal development
3	10 ≤ Age < 13 postconceptional weeks	Early fetal development
4	13 ≤ Age < 16 postconceptional weeks	Early mid-fetal development
5	16 ≤ Age < 19 postconceptional weeks	Early mid-fetal development
6	19 ≤ Age < 24 postconceptional weeks	Late mid-fetal development
7	24 ≤ Age < 38 postconceptional weeks	Late fetal development
8	Birth ≤ Age < 6 postnatal months	Neonatal and early infancy
9	6 ≤ Age < 12 postnatal months	Late infancy
10	1 ≤ Age < 6 years	Early childhood
11	6 ≤ Age < 12 years	Middle and late childhood
12	12 ≤ Age < 20 years	Adolescence
13	20 ≤ Age < 40 years	Young adulthood
14	40 ≤ Age < 60 years	Middle adulthood
15	Age ≥ 60	Late adulthood

Supplementary data 1 – Statistical analysis of results

Basal microelectrode array						
Parameter	Week	Disomic	Trisomic	Test	P-Value	Figure
Active electrodes	4	42.1 ± 5.7 (8 wells)	13.1 ± 7.9 (7 wells)	Mann-Whitney test	0.0140	Figure S3B
	5	48.9 ± 3.6 (11)	18.4 ± 5.6 (9)	Mann-Whitney test	0.0027	
	6	44.18 ± 5.1 (11)	15.4 ± 2.4 (9)	Unpaired t-test	0.0002	
	7	43.9 ± 4.4 (11)	16.9 ± 2.2 (9)	Unpaired t-test	0.0001	
	8	40.6 ± 5.6 (11)	17.5 ± 2.1 (8)	Unpaired t-test	0.0037	
	9	38.7 ± 6.6 (11)	21.2 ± 3 (8)	Unpaired t-test	0.0485	
	10	41.6 ± 7.9 (8)	25.6 ± 3 (7)	Unpaired t-test	0.0948	
	11	47.2 ± 9.6 (5)	26 ± 2.7 (5)	Unpaired t-test	0.0674	
	12	62.8 ± 1 (4)	23.6 ± 2.4 (5)	Unpaired t-test	2.66E-06	
	14	54.5 ± 6.9 (4)	32.5 ± 1.4 (4)	Unpaired t-test	0.0201	
Number of spikes	4	39733 ± 8955 (8 wells)	6304 ± 5305 (7 wells)	Mann-Whitney test	0.0037	Figure S3C
	5	65110 ± 8915 (11)	10050 ± 3879 (9)	Mann-Whitney test	0.000083	
	6	63776 ±12412 (11)	7614 ± 2039 (9)	Unpaired t-test	0.0008	
	7	75609 ± 13041 (11)	9656 ± 1930 (9)	Unpaired t-test	0.0003	
	8	88607 ± 19327 (11)	11293 ± 2329 (8)	Unpaired t-test	0.0036	
	9	101146 ± 24497 (11)	14989 ± 3476 (8)	Unpaired t-test	0.0088	
	10	126481 ± 35296 (8)	18978 ± 3250 (7)	Unpaired t-test	0.0143	
	11	188302 ± 67428 (5)	13930 ± 3355 (5)	Unpaired t-test	0.0325	
	12	354403 ± 44013 (4)	19604 ± 6125 (5)	Unpaired t-test	0.000061	
	14	338730 ±107206 (4)	38555 ± 8773 (4)	Unpaired t-test	0.0316	
Mean firing rate (Hz)	4	1.01 ± 0.2 (8 wells)	0.16 ± 0.13121 (7 wells)	Mann-Whitney test	0.0022	Figure S3D
	5	1.69 ± 0.2 (11)	0.26 ± 0.1 (9)	Mann-Whitney test	0.000083	
	6	1.64 ± 0.3 (11)	0.2 ± 0.05 (9)	Unpaired t-test	0.0007	
	7	1.95 ± 0.3 (11)	0.25 ± 0.1 (9)	Unpaired t-test	0.0003	
	8	2.29 ± 0.5 (11)	0.29 ± 0.1 (8)	Unpaired t-test	0.0036	
	9	2.53 ± 0.6 (11)	0.36 ± 0.1 (8)	Unpaired t-test	0.0081	
	10	3.28 ± 0.9 (8)	0.49 ± 0.1 (7)	Unpaired t-test	0.0145	
	11	4.89 ± 1.7 (5)	0.36 ± 0.1 (5)	Unpaired t-test	0.0325	
	12	9.21 ± 1.1 (4)	0.51 ± 0.16 (5)	Unpaired t-test	0.00006	
	14	8.8 ± 2.8 (4)	1 ± 0.2 (4)	Unpaired t-test	0.0319	
Weighted Mean firing rate (Hz)	4	1.35 ± 0.19 (8 wells)	0.38 ± 0.11 (7 wells)	Unpaired t-test	0.0010	Figure 1C
	5	2.1 ± 0.21 (11)	0.77 ± 0.19 (9)	Unpaired t-test	0.0003	
	6	2.25 ± 0.29 (11)	0.78 ± 0.15 (9)	Unpaired t-test	0.0005	

	7	2.66 ± 0.27 (11)	1.06 ± 0.28 (9)	Unpaired t-test	0.0008	
	8	3.43 ± 0.41 (11)	1.03 ± 0.14 (8)	Unpaired t-test	0.0002	
	9	3.57 ± 0.49 (11)	1.1 ± 0.22 (8)	Unpaired t-test	0.0008	
	10	4.36 ± 0.76 (8)	1.22 ± 0.19 (7)	Unpaired t-test	0.0023	
	11	5.9 ± 1.39 (5)	0.84 ± 0.15 (5)	Unpaired t-test	0.0069	
	12	9.42 ± 1.07 (4)	1.49 ± 0.35 (5)	Unpaired t-test	0.0004	
	14	8.38 ± 2.58 (4)	1.62 ± 0.44 (4)	Unpaired t-test	0.0420	
Electrode bursts	4	1338.37 ± 290.64 (8)	249.28 ± 216.17 (7)	Mann-Whitney test	0.0059	Figure S3E
	5	1843.36 ± 186.06 (11)	468.89 ± 187.29 (9)	Mann-Whitney test	0.00048	
	6	1509.54 ± 260.77 (11)	294.33 ± 89.48 (9)	Unpaired t-test	0.0008	
	7	1883.27 ± 317.45 (11)	292.78 ± 59.7 (9)	Unpaired t-test	0.0003	
	8	1875.36 ± 316.64 (11)	351.87 ± 58.22 (8)	Unpaired t-test	0.0009	
	9	1771.36 ± 369.23 (11)	577.25 ± 129.18 (8)	Unpaired t-test	0.0167	
	10	2254 ± 630.67 (8)	655.14 ± 119.27 (7)	Unpaired t-test	0.0366	
	11	3480.4 ± 1281.92 (5)	437.6 ± 108.26 (5)	Unpaired t-test	0.0456	
	12	6758.75 ± 1001.06 (4)	581.6 ± 146.42 (5)	Unpaired t-test	0.0002	
	14	6235 ± 1780.23 (4)	1045.5 ± 182.7 (4)	Unpaired t-test	0.0273	
Spikes per electrode burst	4	19.03 ± 3.3 (8 wells)	12.17 ± 3.1 (7 wells)	Mann-Whitney test	0.0813	Figure 1D
	5	27.94 ± 5 (11)	10 ± 0.92 (9)	Unpaired t-test	0.0050	
	6	33.6 ± 5.14 (11)	10.44 ± 0.96 (9)	Unpaired t-test	0.0008	
	7	34.91 ± 4.36 (11)	15.58 ± 5.03 (9)	Mann-Whitney test	0.0097	
	8	39.96 ± 6.39 (11)	10.94 ± 0.6 (8)	Unpaired t-test	0.0013	
	9	45.15 ± 6.62 (11)	9.2 ± 0.56 (8)	Unpaired t-test	0.0003	
	10	47.25 ± 9.92 (8)	11.22 ± 0.73 (7)	Unpaired t-test	0.0050	
	11	56.12 ± 9.44 (5)	9.97 ± 1.26 (5)	Unpaired t-test	0.0013	
	12	54.43 ± 10.91 (4)	14.14 ± 2.69 (5)	Unpaired t-test	0.0052	
	14	44.61 ± 15.34 (4)	16.46 ± 1.59 (4)	Unpaired t-test	0.1177	
Burst Duration (s)	4	0.38 ± 0.04 (8 wells)	0.38 ± 0.12 (7 wells)	Mann-Whitney test	0.3450	Figure 1D
	5	0.47 ± 0.04 (11)	0.37 ± 0.04 (9)	Unpaired t-test	0.0832	
	6	0.50 ± 0.04 (11)	0.38 ± 0.04 (9)	Unpaired t-test	0.0593	
	7	0.51 ± 0.04 (11)	0.42 ± 0.04 (9)	Unpaired t-test	0.0971	
	8	0.54 ± 0.05 (11)	0.36 ± 0.02 (8)	Unpaired t-test	0.0105	
	9	0.59 ± 0.05 (11)	0.33 ± 0.02 (8)	Unpaired t-test	0.0006	
	10	0.58 ± 0.06 (8)	0.38 ± 0.03 (7)	Unpaired t-test	0.0151	
	11	0.62 ± 0.05 (5)	0.33 ± 0.03 (5)	Unpaired t-test	0.0007	
	12	0.61 ± 0.06 (4)	0.38 ± 0.03 (5)	Unpaired t-test	0.0086	
	14	0.54 ± 0.09 (4)	0.4 ± 0.04 (4)	Unpaired t-test	0.2013	

Inter-burst interval	4	48.32 ± 7.12 (8 wells)	61.07 ± 11.54 (7 wells)	Unpaired t-test	0.3420	Figure 1D
	5	34.92 ± 4.93 (11)	51.96 ± 4.39 (9)	Mann-Whitney test	0.0057	
	6	38.04 ± 5.34 (11)	46.42 ± 7.67 (9)	Unpaired t-test	0.3689	
	7	29.65 ± 2.36 (11)	44.65 ± 4.96 (9)	Unpaired t-test	0.0095	
	8	29.36 ± 4.83 (11)	49.84 ± 8.94 (8)	Unpaired t-test	0.0448	
	9	33.09 ± 4.08 (11)	43.58 ± 7.39 (8)	Mann-Whitney test	0.3100	
	10	25.68 ± 4.05 (8)	43.67 ± 7.05 (7)	Unpaired t-test	0.0396	
	11	23.28 ± 5.64 (5)	59.38 ± 9.47 (5)	Unpaired t-test	0.0113	
	12	11.55 ± 1.1 (4)	38.61 ± 10.36 (5)	Unpaired t-test	0.0552	
	14	13.83 ± 5.63 (4)	45.28 ± 2.91 (4)	Unpaired t-test	0.0025	
Number of network bursts	4	23 ± 5.58 (8 wells)	7.71 ± 7.71 (7 wells)	Mann-Whitney test	0.0545	Figure S3F
	5	24.82 ± 2.73 (11)	10.44 ± 4.99 (9)	Unpaired t-test	0.0162	
	6	34.18 ± 8.47 (11)	9.78 ± 4.102 (9)	Unpaired t-test	0.0266	
	7	30.54 ± 4.49 (11)	11.89 ± 2.23 (9)	Mann-Whitney	0.0028	
	8	39.54 ± 5.03 (11)	12.37 ± 2.46 (8)	Unpaired t-test	0.0005	
	9	36.09 ± 3.61 (11)	21.12 ± 2.49 (8)	Mann-Whitney test	0.0105	
	10	46.12 ± 6.64 (8)	27 ± 3.46 (7)	Unpaired t-test	0.0296	
	11	39.8 ± 6.25 (5)	24.4 ± 1.86 (5)	Unpaired t-test	0.0458	
	12	43.75 ± 5.86 (4)	25.6 ± 2.09 (5)	Unpaired t-test	0.0150	
	14	52.5 ± 1.55 (4)	33.25 ± 1.11 (4)	Unpaired t-test	<0.0001	
Network burst frequency	4	0.043 ± 0.01 (7 wells)	0 (5 wells)	Mann-Whitney test	0.0006	Figure 1E
	5	0.041 ± 0.004 (11)	0.031 ± 0.012 (5)	Unpaired t-test	0.3430	
	6	0.062 ± 0.01 (10)	0.029 ± 0.01 (5)	Unpaired t-test	0.1423	
	7	0.05 ± 0.01 (11)	0.02 ± 0.004 (9)	Mann-Whitney test	0.0021	
	8	0.07 ± 0.01 (11)	0.02 ± 0.004 (8)	Unpaired t-test	0.0005	
	9	0.057 ± 0.01 (11)	0.034 ± 0.004 (8)	Unpaired t-test	0.0090	
	10	0.076 ± 0.01 (8)	0.045 ± 0.01 (7)	Mann-Whitney test	0.0078	
	11	0.066 ± 0.01 (5)	0.041 ± 0.0031 (5)	Unpaired t-test	0.0448	
	12	0.073 ± 0.01 (4)	0.043 ± 0.0035 (5)	Unpaired t-test	0.0137	
	14	0.087 ± 0.0026 (4)	0.055 ± 0.0018 (4)	Unpaired t-test	5.49E-05	
Spikes per network burst	4	1397 ± 226 (7 wells)	0 (7 wells)	Unpaired t-test	0.0004	Figure 1E
	5	2001 ± 267 (11)	183 ± 75 (5)	Unpaired t-test	0.0005	
	6	1882 ± 514 (10)	229 ± 49 (5)	Mann-Whitney test	0.0007	
	7	2277 ± 453 (11)	314 ± 80 (9)	Unpaired t-test	0.0011	
	8	2099 ± 555 (11)	292 ± 77 (8)	Mann-Whitney test	0.0002	
	9	2372 ± 571 (11)	380 ± 91 (8)	Unpaired t-test	0.0093	
	10	2451 ± 688 (8)	516 ± 101 (7)	Unpaired t-test	0.0221	
	11	4819 ± 2074 (5)	402 ± 110 (5)	Unpaired t-test	0.0661	

	12	8372 ± 1285 (4)	563 ± 191 (5)	Unpaired t-test	0.0003	
	14	6163 ± 2032 (4)	957 ± 237 (4)	Unpaired t-test	0.0438	
Synchrony (area under normalised cross-correlation curve)	4	0.047 ± 0.022 (7 wells)	0.001 ± 6.67E-4 (7 wells)	Mann-Whitney test	0.0073	Figure 1F
	5	0.07493 ± 0.025 (11)	0.00149 ± 4.88e-4 (5)	Mann-Whitney test	0.0005	
	6	0.09433 ± 0.023 (10)	0.00195 ± 4.03e-4 (5)	Unpaired t-test	0.0009	
	7	0.10019 ± 0.022 (11)	0.00513 ± 0.002 (9)	Mann-Whitney test	0.0006	
	8	0.10471 ± 0.029 (11)	0.00298 ± 6.36e-4 (8)	Mann-Whitney test	0.0005	
	9	0.12173 ± 0.03 (11)	0.0039 ± 6.71e-4 (8)	Unpaired t-test	0.0044	
	10	0.14083 ± 0.037 (8)	0.00727 ± 9.94e-4 (7)	Unpaired t-test	0.0080	
	11	0.16045 ± 0.034 (5)	0.00648 ± 0.001 (5)	Unpaired t-test	0.0018	
	12	0.19306 ± 0.04 (4)	0.00742 ± 0.001 (5)	Unpaired t-test	0.0011	
	14	0.16143 ± 0.057 (4)	0.01079 ± 0.001 (4)	Unpaired t-test	0.0363	
**Microelectrode array - pharmacology in CNQX and APV**						
Parameter	Control	CNQX+APV	Wash	Test	P-value	Figure
Normalised mean firing rate (Hz)	100 (6 wells)	74.1 ± 9.4 (6 wells)	94.5 ± 2.6 (6 wells)	One way-ANOVA	0.0132	Figure S3G-J
Normalised number of bursts	100 (6 wells)	80.4 ± 4.2 (6 wells)	95.2 ± 2.7 (6 wells)	One way-ANOVA	0.0006	
Normalised no. of network bursts	100 (6 wells)	18.3 ± 15.5 (6 wells)	115.3 ± 8.4 (6 wells)	One way-ANOVA	P<0.0001	
Normalised synchrony index	100 (6 wells)	44.8 ± 7.7 (6 wells)	102.5 ± 3.3 (6 wells)	One way-ANOVA	P<0.0001	
**Microelectrode array - pharmacology in tetrodotoxin**						
Parameter	Control	Tetrodotoxin	Wash	Test	P-value	Figure
Normalised mean firing rate (Hz)	100 (8 wells)	0.003 ± 0.002 (8 wells)	69.7 ± 3.1 (8 wells)	One way-ANOVA	P<0.0001	Figure S3G-J
Normalised number of bursts	100 (8 wells)	0 ± 0 (8 wells)	78.3 ± 4.1 (8 wells)	One way-ANOVA	P<0.0001	
Normalised no. of network bursts	100 (8 wells)	0 ± 0 (8 wells)	71.4 ± 13.7 (8 wells)	One way-ANOVA	P<0.0001	
Normalised synchrony index	100 (8 wells)	0 ± 0 (8 wells)	78.9 ± 5.2 (8 wells)	One way-ANOVA	P<0.0001	
**Microelectrode array – clonal parameters for disomic cells**						
Parameter	Genotype	C3 clone	C9 clone	Test	P-value	Figure
Active electrodes	Disomic	56 (13 wells)	53 (6 wells)	Mann-Whitney test	0.6928	Figure S2A
Weighted mean firing rate (Hz)	Disomic	3.9 ± 0.6 (13 wells)	3.9 ± 0.5 (6 wells)	Unpaired t-test	0.967	
Number of electrode bursts	Disomic	2100.2 ± 456 (13 wells)	1702.5 ± 418.7 (6 wells)	Unpaired t-test	0.5955	
Number of network bursts	Disomic	37 (13 wells)	34 (6 wells)	Mann-Whitney test	0.9301	
Synchrony	Disomic	0.06592 (13 wells)	0.1353 (6 wells)	Mann-Whitney test	0.467	
**Microelectrode array – clonal parameters for trisomic cells**						
Parameter	Genotype	C5 clone	C13 clone	Test	P-value	Figure
Active electrodes	Trisomic	22 ± 2.3 (9 wells)	25.2 ± 4.4 (6 wells)	Unpaired t-test	0.492	Figure S2A
Weighted mean firing rate (Hz)	Trisomic	1.14 ± 0.2 (9 wells)	1.17 ± 0.1 (6 wells)	Unpaired t-test	0.9347	
Number of electrode bursts	Trisomic	551 ± 118.1 (9 wells)	707.5 ± 124.1 (6 wells)	Unpaired t-test	0.3927	
Number of network bursts	Trisomic	22.1 ± 3.3 (9 wells)	26.5 ± 2.1 (6 wells)	Unpaired t-test	0.3373	
Synchrony	Trisomic	0.005 ± 0.001 (9 wells)	0.006 ± 0.001 (6 wells)	Unpaired t-test	0.3088	

Excitatory postsynaptic currents (EPSC)						
Parameter	Condition	Disomic	Trisomic	Test	P-value	Figure
EPSC Frequency (Hz)	In Mg^{2+}	2.020 (39 cells)	0.56 (32 cells)	Mann Whitney test	0.00006528	Figure 2C
	0 Mg^{2+}	7.242 (16 cells)	0.7312 (19 cells)	Mann Whitney test	0.00002789	Figure 2H
EPSC Amplitude (pA)	In Mg^{2+}	-36.6 ± 2.2 (39 cells)	-37.7 ± 2.8 (30)	Unpaired t-test	0.762	Figure 2D
	0 Mg^{2+}	-37.25 ± 2.9 (14 cells)	-32.2 ± 1.2 (16 cells)	Unpaired t-test	0.1011	Figure 2I
EPSC kinetics (charge transfer; pA.ms)	In Mg^{2+}	-73.8 ± 7 (27 cells)	-78.8 ± 6.4 (25 cells)	Unpaired t-test	0.6069	Figure S4G
	0 Mg^{2+}	-54.885 (9 cells)	-71.574 (10 cells)	Mann Whitney test	0.3562	Figure S4I
EPSC kinetics (rate of rise; $pAms^{-1}$)	In Mg^{2+}	393.24 (19 cells)	369.77 (25 cells)	Mann Whitney test	0.8311	Figure S4G
	0 Mg^{2+}	350.02 (9 cells)	319.08 (10 cells)	Mann Whitney test	0.447	Figure S4I
EPSC kinetics (decay τ; ms)	In Mg^{2+}	2.277 ± 0.23 (18 cells)	2.153 ± 0.14 (24 cells)	Unpaired t-test	0.6269	Figure S4G
	0 Mg^{2+}	2.187 ± 0.32 (8 cells)	2.447 ± 0.29 (10 cells)	Unpaired t-test	0.5598	Figure S4I
**Excitatory postsynaptic currents (EPSC) – clonal parameters for disomic cells**						
Parameter	Genotype	C3 clone	C9 clone	Test	P-value	Figure
EPSC Frequency (Hz)	Disomic	9.2 ± 2 (9 cells)	7.7 ± 2.5 (7 cells)	Unpaired t-test	0.6488	Figure S2E
**Excitatory postsynaptic currents (EPSC)– clonal parameters for trisomic cells**						
Parameter	Genotype	C5 clone	C13 clone	Test	P-value	Figure
EPSC Frequency (Hz)	Trisomic	0.9839 (12 cells)	0.6024 (7 cells)	Mann Whitney test	0.8369	Figure S2E
**Bursts in 0 Mg^{2+}**						
Parameter		Disomic	Trisomic	Test	P-value	Figure
Bursts rate (min^{-1})		1.319 (20 cells)	0.8745 (28 cells)	Mann Whitney test	0.0395	Figure 3C
Burst amplitude (pA)		117.07 (20 cells)	42.7 (19 cells)	Mann Whitney test	0.0002	Figure 3D
Burst charge transfer (pA.ms)		-722392 ± 161212 (18)	-226700 ± 33474 (17)	Unpaired t-test	0.0061	Figure 3E
Capacitance in Cs-based internal (pF)		89.524 (51 cells)	69.762 (56 cells)	Mann Whitney test	0.0771	Figure S4E
**Action potentials**						
Parameter		Disomic	Trisomic	Test	P-value	Figure
Resting membrane potential (mV)		-50 (112 cells)	-44 (100 cells)	Mann Whitney test	0.061	Figure 4A
Rheobase (pA)		24.5 (72 cells)	16 (62 cells)	Mann Whitney test	0.0939	Figure 4B
Spike threshold (mV)		-34 (85 cells)	-35 (87 cells)	Mann Whitney test	0.1653	Figure 4E
I at max spiking (pA)		72.5 (78 cells)	55 (83 cells)	Mann Whitney test	0.002	Figure 4F
Spike peak (mV)		78.7 ± 2.4 (70 cells)	66.3 ± 3.5 (60 cells)	Unpaired t-test	0.0032	Figure 4J
Spike area (mV.ms)		169.4 (70 cells)	192.80 (60 cells)	Mann Whitney test	0.000033	Figure 4K
Rise time (ms)		1.437 (70 cells)	2 (60 cells)	Mann Whitney test	0.0052	Figure 4L
T_{50} (ms)		1.087 (70 cells)	1.508 (60 cells)	Mann Whitney test	0.000038	Figure 4M
Capacitance using a K-based internal (pF)		72.803 (113 cells)	58.216 (114 cells)	Mann Whitney test	0.0532	Figure S6D
Input resistance using a K-based internal (MΩ)		544.10 (113 cells)	511.73 (114 cells)	Mann Whitney test	0.9557	Figure S6D

Supplementary data 1 – Statistical analysis of results

Membrane time constant (ms)		0.6218 (113 cells)	0.5511 (114 cells)	Mann Whitney test	0.215	Figure S6D
Average number of spikes	Rheo x 1	1 (72 cells)	1 (62 cells)	-	-	Figure 4D
	Rheo x 2	3.583 ± 0.15 (72 cells)	3.095 ± 0.17 (62 cells)	Unpaired t-test	0.0351	
	Rheo x 3	5.035 ± 0.21 (72 cells)	3.994 ± 0.26 (62 cells)	Mann Whitney test	0.0055	
	Rheo x 4	5.694 ± 0.25 (72 cells)	4.258 ± 0.3 (62 cells)	Mann Whitney test	0.0004	
Average latency at first spike (s)	Rheo x 1	0.227 ± 0.007 (64 cells)	0.194 ± 0.01 (57 cells)	Mann Whitney test	0.0408	Figure 4G
	Rheo x 2	0.069 ± 0.004 (64 cells)	0.062 ± 0.004 (57 cells)	Unpaired t-test	0.2537	
	Rheo x 3	0.042 ± 0.003 (64 cells)	0.037 ± 0.003 (57 cells)	Unpaired t-test	0.1517	
	Rheo x 4	0.03 ± 0.002 (64 cells)	0.026 ± 0.002 (57 cells)	Unpaired t-test	0.2388	
Spike jitter		0.1235 (65 cells)	0.1457 (54 cells)	Mann Whitney test	0.0412	Figure 4H
**Current clamp – clonal parameters for disomic cells**						
Parameter	Genotype	C3 clone	C9 clone	Test	P-value	Figure
V_m (mV)	Disomic	-48 (47 cells)	-53 (57 cells)	Mann Whitney test	0.0812	Figure S2C
Rheobase (pA)	Disomic	28 (39 cells)	17 (33 cells)	Mann Whitney test	0.1824	Figure S2D
**Current clamp – clonal parameters for trisomic cells**						
Parameter	Genotype	C3 clone	C9 clone	Test	P-value	Figure
V_m (mV)	Trisomic	-41.931 ± 1.5 (87 cells)	-46.741 ± 2.6 (27 cells)	Unpaired t-test	0.1157	Figure S2C
Rheobase (pA)	Trisomic	17 (46 cells)	14.25 (16 cells)	Mann Whitney test	0.1206	Figure S2D
**Na^+ channel properties**						
Parameter		Disomic	Trisomic	Test	P-value	Figure
I_{Na} density (pA/ pF)		-54.635 (52 cells)	-42.209 (31 cells)	Mann Whitney test	0.2854	Figure 5A
50% fast inactivation (mV)		-38.83 ± 0.9 (29 cells)	-44.31 ± 0.6 (16 cells)	Unpaired t-test	P<0.0001	Figure 5C
50% slow inactivation (mV)		-42.7 ± 0.4 (29 cells)	-46.1 ± 0.3 (14 cells)	Unpaired t-test	P<0.0001	Figure 5D
**Na^+ channel properties – clonal parameters for disomic cells**						
Parameter	Genotype	C3 clone	C9 clone	Test	P-value	Figure
I_{Na} density (pA/ pF)	Disomic	-65.328 (27 cells)	-42.284 (28 cells)	Mann Whitney test	0.1008	Figure S2F
**Na^+ channel properties – clonal parameters for trisomic cells**						
Parameter	Genotype	C5 clone	C13 clone	Test	P-value	Figure
I_{Na} density (pA/ pF)	Trisomic	-38.188 (14 cells)	-54.403 (17 cells)	Mann Whitney test	0.3163	Figure S2F
**K^+ channel properties – IV curve**						
Parameter	Condition	Disomic	Trisomic	Test	P-value	Figure
K^+ IV curve (current density; pA/ pF)	-110 mV step	-3.58 ± 0.3 (56 cells)	-4.60 ± 0.54 (35 cells)	Unpaired t-test	ns	Figure 6B
	-100 mV step	-2.83 ± 0.24 (56 cells)	-3.76 ± 0.47 (35 cells)	Unpaired t-test	ns	
	-90 mV step	-2.13 ± 0.18 (56 cells)	-2.93 ± 0.39 (35 cells)	Unpaired t-test	ns	
	-80 mV step	-1.49 ± 0.13 (56 cells)	-2.07 ± 0.33 (35 cells)	Unpaired t-test	ns	
	-70 mV step	-0.83 ± 0.08 (56 cells)	-1.21 ± 0.28 (35 cells)	Unpaired t-test	ns	
	-60 mV step	-0.21 ± 0.06 (56 cells)	-0.54 ± 0.23 (35 cells)	Unpaired t-test	ns	
	-50 mV step	0.16 ± 0.07 (56 cells)	-0.05 ± 0.22 (35 cells)	Unpaired t-test	ns	

	-40 mV step	0.53 ± 0.09 (56 cells)	0.42 ± 0.22 (35 cells)	Unpaired t-test	ns	
	-30 mV step	0.88 ± 0.13 (56 cells)	0.91 ± 0.24 (35 cells)	Unpaired t-test	ns	
	-20 mV step	1.48 ± 0.19 (56 cells)	1.63 ± 0.31 (35 cells)	Unpaired t-test	ns	
	-10 mV step	3.07 ± 0.33 (56 cells)	2.79 ± 0.46 (35 cells)	Unpaired t-test	ns	
	0 mV step	6.1 ± 0.56 (56 cells)	4.91 ± 0.68 (35 cells)	Mann-Whitney test	0.0860	
	10 mV step	10.34 ± 0.83 (56 cells)	8.08 ± 0.99 (35 cells)	Mann-Whitney test	0.0241	
	20 mV step	15.52 ± 1.17 (56 cells)	11.99 ± 1.39 (35 cells)	Mann-Whitney test	0.0186	
	30 mV step	21.41 ± 1.56 (56 cells)	16.47 ± 1.82 (35 cells)	Mann-Whitney test	0.0182	
	40 mV step	27.94 ±1.98 (56 cells)	21.72 ± 2.27 (35 cells)	Mann-Whitney test	0.0212	
	50 mV step	34.87 ± 2.3 (56 cells)	27.4 ± 2.83 (35 cells)	Mann-Whitney test	0.0182	
	60 mV step	42.01 ± 2.75 (56 cells)	33.4 ± 3.45 (35 cells)	Mann-Whitney test	0.0171	
	70 mV step	49.44 ± 3.1 (56 cells)	39.47 ± 4.1 (35 cells)	Mann-Whitney test	0.0101	
	80 mV step	56.46 ± 3.39 (56 cells)	45.53 ± 4.76 (35 cells)	Mann-Whitney test	0.0079	
	90 mV step	63.31 ± 3.67 (56 cells)	50.9 ± 5.34 (35 cells)	Mann-Whitney test	0.003	
$I_{k(A\ current)}$ (pA/ pF)	Basal	29.145 (45 cells)	24.736 (31 cells)	Mann-Whitney test	0.0311	Figure 6D
A-current inhibition(% control)	+TEA	16.3 ± 5.5 (24 cells)	38.9 ± 4.9 (23 cells)	Unpaired t-test	0.0038	Figure 6E
	+TEA+4AP	67.8 ± 3.8 (12 cells)	81 ± 3.9 (11 cells)	Unpaired t-test	0.0251	Figure 6E
**K^+ channel properties – clonal parameters for disomic cells**						
Parameter	Genotype	C3 clone	C9 clone	Test	P-value	Figure
K^+ I-V (*I* density at 90 mV; pA/ pF)	Disomic	64.362 ± 4.9 (28 cells)	62.260 ± 5.3 (28 cells)	Unpaired t-test	0.7618	Figure S2G
$I_{k(A\ current)}$ (pA/ pF)	Disomic	30.578 (22 cells)	29.145 (23 cells)	Mann-Whitney test	0.5474	Figure S2H
**K^+ channel properties – clonal parameters for trisomic cells**						
Parameter	Genotype	C5 clone	C13 clone	Test	P-value	Figure
K^+ I-V (*I* density at 90 mV; pA/ pF)	Trisomic	40.471 (24 cells)	43.615 (11 cells)	Mann-Whitney test	0.6617	Figure S2G
$I_{k(A\ current)}$ (pA/ pF)	Trisomic	26.2 (15 cells)	26.13 (16 cells)	Mann-Whitney test	0.7518	Figure S2H
**Capacitance – clonal parameters for disomic cells**						
Parameter	Genotype	C3 clone	C9 clone	Test	P-value	Figure
In Cs-based internal	Disomic	86.8 ± 7 (33 cells)	103.23 ± 10.7 (18 cells)	Unpaired t-test	0.1882	Figure S2B
In K-based internal	Disomic	70.559 (56 cells)	77.141 (57 cells)	Mann-Whitney test	0.2181	Figure S2B
**Capacitance – clonal parameters for trisomic cells**						
Parameter	Genotype	C5 clone	C13 clone	Test	P-value	Figure
In Cs-based internal	Trisomic	86.2 ± 8.5 (31 cells)	71.6 ± 6.7 (25 cells)	Unpaired t-test	0.1865	Figure S2B
In K-based internal	Trisomic	62.174 (87 cells)	46.309 (27 cells)	Mann-Whitney test	0.0593	Figure S2B
**Immunofluorescence staining**						
Label		Disomic	Trisomic	Test	P-value	Figure
Synapsin-1/ MAP2		0.32 ± 0.03 (11 stacks)	0.18 ± 0.012 (11 stacks)	Unpaired t-test	0.0002	Figure 2K
Synapsin-1/ SMI-321		1.11 ± 0.06 (11 stacks)	0.68 ± 0.075 (11 stacks)	Unpaired t-test	0.0003	Figure S5A
PSD95/ MAP2		0.2 ± 0.01 (10 stacks)	0.15 ± 0.01 (10 stacks)	Unpaired t-test	0.0011	Figure S5B

PSD95/ SMI-321		0.81 ± 0.07 (10 stacks)	0.46 ± 0.045 (10 stacks)	Unpaired t-test	0.0008	Figure S5B
**RNA expression**						
Gene	Sliding window	Control	Down syndrome	Test	P-Value	Figure
KCND3	5 to 8	7.51 ± SEM 0.29 (3)	6.11 ± SEM 0.43 (3)	Paired t-test	0.010824	Figure 7B
	6 to 9	7.24 ± 0.3 (4)	6.47 ± 0.35 (4)	Paired t-test	0.160675	
	8 to 10	7.14 ± 0.33 (4)	6.86 ± 0.07 (4)	Paired t-test	0.477507	
	9 to 11	7.16 ± 0.35 (4)	6.87 ± 0.07 (4)	Paired t-test	0.470149	
	10 to 12	7.99 ± 0.44 (4)	6.96 ± 0.26 (4)	Paired t-test	0.142504	
	11 to 13	8.26 ± 0.26 (5)	7.13 ± 0.3 (5)	Paired t-test	0.097811	
	12 to 14	8.33 ± 0.27 (5)	7.23 ± 0.3 (5)	Paired t-test	0.104055	
KCND2	5 to 8	9.46 ± SEM 0.18 (3)	8.59 ± SEM 0.77 (3)	Paired t-test	0.278329	Figure 7C
	6 to 9	8.86 ± 0.48 (4)	9.09 ± 0.42 (4)	Paired t-test	0.738238	
	8 to 10	8.28 ± 0.62 (4)	8.83 ± 0.61 (4)	Paired t-test	0.346505	
	9 to 11	8.21 ± 0.56 (4)	8.3 ± 0.48 (4)	Paired t-test	0.910073	
	10 to 12	8.65 ± 0.62 (4)	7.51 ± 0.54 (4)	Paired t-test	0.137881	
	11 to 13	9.23 ± 0.22 (5)	7.63 ± 0.47 (5)	Paired t-test	0.004834	
	12 to 14	9.17 ± 0.2 (5)	7.42 ± 0.48 (5)	Paired t-test	0.005844	
KCNC3	5 to 8	6.39 ± SEM 0.36 (3)	6.15 ± SEM 0.34 (3)	Paired t-test	0.072349	Figure 7D
	6 to 9	6.39 ± 0.25 (4)	6.3 ± 0.26 (4)	Paired t-test	0.711641	
	8 to 10	6.6 ± 0.22 (4)	6.49 ± 0.22 (4)	Paired t-test	0.673465	
	9 to 11	6.41 ± 0.14 (4)	6.3 ± 0.22 (4)	Paired t-test	0.640508	
	10 to 12	6.92 ± 0.25 (4)	6.46 ± 0.15 (4)	Paired t-test	0.100199	
	11 to 13	7.1 ± 0.24 (5)	6.28 ± 0.13 (5)	Paired t-test	0.026606	
	12 to 14	7.09 ± 0.24 (5)	6.33 ± 0.11 (5)	Paired t-test	0.049084	
KCNC4	5 to 8	6.76 ± SEM 0.4 (3)	6.21 ± SEM 0.38 (3)	Paired t-test	0.003055	Figure 7E
	6 to 9	6.93 ± 0.23 (4)	6.44 ± 0.23 (4)	Paired t-test	0.036929	
	8 to 10	7.12 ± 0.16 (4)	6.64 ± 0.17 (4)	Paired t-test	0.041127	
	9 to 11	7.06 ± 0.12 (4)	6.53 ± 0.14 (4)	Paired t-test	0.046405	
	10 to 12	7.23 ± 0.23 (4)	6.38 ± 0.28 (4)	Paired t-test	0.179934	
	11 to 13	7.16 ± 0.2 (5)	6.32 ± 0.2 (5)	Paired t-test	0.090801	
	12 to 14	7.09 ± 0.19 (5)	6.3 ± 0.2 (5)	Paired t-test	0.10978	
**Fluorescent in-situ hybridization (FISH)**						
FISH type	Number	Disomic	Trisomic	Figure		
% FISH (chromosome 21)	1	1.6	1.4	Figure S1B		
	2	96.8	4.9			
	3	1.6	88.73			
	>3	0	4.9			

% FISH (chromosome 13)	1	0	2.83
	2	99.2	96.45
	3	0.8	0
	>3	0	0.7

**Whole-cell agonist-activated current densities (pA/ pF)**					
Agonist	Disomic	Trisomic	Test	P-value	Figure
GABA	30.5 ± 17.3 (5 cells)	36.8 ± 14.2 (8 cells)	Unpaired t-test	0.7856	Figure S4D
AMPA (+cyclothiazine)	0.1250 (5 cells)	0.8947 (7 cells)	Mann-Whitney test	0.7432	Figure S4K
NMDA (+glycine)	0.129 ± 0.13 (5 cells)	0.194 ± 0.12 (5 cells)	Unpaired t-test	0.7221	Figure S4K
**mRNA levels (qPCR)**					
Gene	Disomic	Trisomic	Test	P-value	Figure
KCND3/ β-actin (primer set 1)	13.12 ± 0.1 (3 samples)	11.45 ± 0.1 (3 samples)	Paired t-test	0.0005	Figure 7F
KCND3/ β-actin (primer set 2)	12.97 ± 0.1 (3 samples)	10.93 ± 0.1 (3 samples)	Paired t-test	0.0002	Not plotted
KCND3/ β-actin (primer set 3)	12.96 ± 0.1 (3 samples)	10.86 ± 0.1 (3 samples)	Paired t-test	0.0002	Not plotted
**Western blot**					
Protein	Disomic	Trisomic	Test	P-value	Figure
Kv4.3/ β-actin	0.98 ± 0.1 (3 samples)	0.57 ± 0.05 (3 samples)	Paired t-test	0.0468	Figure 7G
**Protein expression (Immunofluorescence)**					
Protein	Disomic	Trisomic	Test	P-value	Figure
Kv4.3	0.19 ± 0.01 (6 stacks)	0.14 ± 0.004 (5 stacks)	Unpaired t-test	0.0245	Figure 7H, S9B
Kv4.2	0.24 ± 0.03 (5 stacks)	0.24 ± 0.03 (5 stacks)	Unpaired t-test	0.8833	Figure S9C
**Pie chart – neuronal identity probed using electrophysiology**					
Parameter	Neuronal	Non-neuronal	Figure		
% neuronal cells	98.2% (228/ 216 cells)	1.8% (4/ 216 cells)	Figure S1F		
**Pie chart – spike type of cells**					
Spike type	Disomic	Trisomic	Figure		
Single spiker	5.1% (5/ 98 cells)	10.1% (10/ 99 cells)	Figure S1G		
Double spiker	2% (2/ 98 cells)	8.1% (8/ 99 cells)	Figure S1G		
Multi Spiker	92.9% (91/ 98 cells)	81.8% (81/ 99 cells)	Figure S1G		
**Pie chart – cells receiving glutamatergic inputs**					
Parameter	Disomic	Trisomic	Figure		
%Cells receiving excitatory postsynaptic inputs in Mg^{2+}	96% (48/ 50)	80% (33/ 41)	Figure 2A		
%Cells receiving excitatory postsynaptic inputs in 0 Mg^{2+}	100% (49/ 49)	90% (53/ 59)	Figure 2F		
%Cells that undergo bursting in 0 Mg^{2+}	100% (37/ 37)	63% (27/ 43)	Figure 3A		
%Cells receiving inhibitory postsynaptic inputs in Mg^{2+}	12% (4/ 34)	2% (1/ 47)	Figure S4A		

Supplementary data 2 – Expression of K^+ channel, HCN channel, Na^+ channel and glutamatergic synapse genes in human dorsolateral prefrontal cortex across development and adulthood

Group	Gene	ID_REF	Con_DFC_S	Con_DFC_S	Con_DFC_S	Con_DFC_S	Con_DFC_S	Con_DFC_S	Con_DFC_S	Con_DFC_S	Con_DFC_S	Con_DFC_S	Con_DFC_S	Con_DFC_S	DS_DFC_St	DS_DFC_St	DS_DFC_St	DS_DFC_St	DS_DFC_St	DS_DFC_St	DS_DFC_St	DS_DFC_St	DS_DFC_St	DS_DFC_St	DS_DFC_St	DS_DFC_St	Log2FC	pvalue	FDR
Potassium	KCNF1	2469627	6.39239	6.5778	7.13753	6.75006	7.49189	6.45035	7.58204	7.16916	6.74213	7.20943	7.28432	6.63193	5.61782	5.53562	6.02626	6.98758	5.93758	6.55342	7.24814	6.98819	5.58086	6.06083	6.36482	5.16142	-0.77971	0.000873	0.05716
Potassium	KCNC4	2351294	6.27012	6.44236	7.55261	6.80305	6.93213	7.18034	7.3342	6.64058	7.77345	6.86981	7.18699	7.0007	5.71625	5.95901	6.96811	6.67611	6.14736	6.76083	6.51775	6.68477	5.53909	6.43926	6.40295	6.45597	-0.64324	0.002234	0.077798
Potassium	KCNK3	2473936	5.89775	6.32896	7.69524	6.46529	6.99961	6.44722	7.25075	6.91869	6.74702	6.94704	6.45617	6.55479	6.03326	5.59506	6.01718	6.64304	6.93064	6.23675	6.30418	6.56561	5.76706	5.88345	5.77188	5.69554	-0.60541	0.003012	0.084342
Potassium	KCNH1	2454140	5.16101	4.84467	7.96398	8.33697	8.67737	9.19243	9.78577	9.63783	9.65335	9.59559	9.51188	9.79924	4.59501	5.0093	6.66936	8.96639	7.74935	8.75632	9.17215	9.17752	8.31263	8.37919	9.00603	8.7575	-0.63411	0.003431	0.088655
Potassium	KCNQ3	3154002	9.53081	10.035	10.7814	9.6019	10.3239	9.93733	10.0966	10.2831	9.71771	9.31478	9.1142	9.56213	9.48693	9.18744	10.2387	9.70064	10.0051	9.86856	8.54037	9.57086	8.54479	8.8483	8.73257	9.36948	-0.51709	0.003722	0.090748
Potassium	KCNK5	2952834	4.47159	5.45324	4.95615	5.55506	5.16784	4.93428	4.76816	5.18213	4.81433	5.14154	5.2192	4.9394	5.13816	5.64674	4.94418	5.26268	5.64688	5.75236	5.79016	5.14018	5.94944	5.61094	5.4494	5.62798	0.446348	0.005048	0.102608
Potassium	KCNC2	3462567	8.57515	8.03813	10.1676	9.30907	11.0614	8.83374	11.0401	11.0446	10.408	10.1779	9.76954	10.6529	6.53161	6.77728	8.93292	9.75532	9.84525	9.11858	10.0797	10.4501	8.2694	9.19981	10.2208	9.4255	-0.87266	0.005393	0.104738
Potassium	KCND3	2428119	7.33767	7.11952	8.07453	6.63761	7.14137	6.70106	8.16815	8.52788	8.56639	8.75992	7.28641	8.52539	5.97753	5.45133	6.91	6.87384	6.66378	6.99447	6.93166	7.5868	6.32823	6.76769	8.03281	7.44016	-0.9073	0.006331	0.110913
Potassium	KCNB2	3103062	7.29499	8.45801	8.03907	7.1275	6.91407	6.95024	8.25618	8.15796	7.25266	8.13209	7.16823	8.41306	7.72697	7.89809	8.0219	7.39271	6.00691	6.91937	6.7248	6.95143	6.19913	6.79339	6.70716	6.55303	-0.6891	0.008261	0.120192
Potassium	KCNA1	3401994	4.91811	5.32056	9.10055	6.34283	8.58671	7.22598	10.1692	9.75657	9.5546	10.2357	9.77793	9.63716	5.05551	4.61726	6.09208	8.19831	6.95175	7.82181	7.63647	7.62237	6.9652	7.14306	7.6498	7.40626	-1.4555	0.008499	0.120808
Potassium	KCNN2	2824581	5.76807	5.6336	6.65	7.41097	6.6624	6.29742	6.78941	6.33577	6.15071	6.5101	6.19584	6.10648	6.1827	5.40431	6.11585	6.33526	6.19469	6.08924	6.29291	6.21134	5.5021	6.12535	5.14661	6.26827	-0.38685	0.010592	0.12799
Potassium	KCNH3	3414029	5.99398	6.22568	8.88799	9.09199	8.78702	9.076	8.76493	8.538	9.00205	8.81209	8.58579	7.78912	6.20229	6.67408	7.99192	8.36271	8.18963	8.62902	8.16188	8.56407	8.41898	8.03332	8.02807	7.84853	-0.37085	0.01363	0.13863
Potassium	KCNJ5	3355860	4.03973	4.22356	5.4636	4.81094	4.76937	4.27727	4.31859	4.65457	4.12746	4.30713	4.607	4.53849	4.26957	4.41793	6.05625	4.75126	4.5783	4.66752	5.02338	4.54585	5.06309	4.89779	4.53983	4.94632	0.301615	0.01493	0.142999
Potassium	KCNQ4	2332091	5.37082	5.79189	6.08963	5.63986	5.74856	5.89283	5.60941	6.31125	6.21947	6.21783	6.01727	6.01096	5.7405	6.09462	6.14349	5.88629	6.20538	6.13072	6.29645	6.2543	6.4057	6.01136	5.91395	6.33895	0.208494	0.015795	0.145955
Potassium	KCNH6	3730698	4.66758	5.42541	4.89781	4.71567	4.8218	4.95472	4.8561	5.71466	4.91909	4.68073	5.06488	5.0801	5.50544	5.38668	5.019	5.10241	5.1832	5.13796	5.76063	5.28123	5.9622	5.21707	5.41572	5.07046	0.353621	0.015879	0.146272
Potassium	KCNG4	3702429	4.00174	4.26955	3.99932	4.39467	3.98626	4.00194	4.21675	4.22862	4.06527	4.38053	4.20104	4.30394	4.54873	4.17846	4.27021	4.31306	4.36501	4.04328	4.41263	4.33338	4.57235	4.58502	4.37449	4.21395	0.180078	0.016029	0.1469
Potassium	KCNB1	3908901	7.50216	7.67993	9.48992	7.80112	8.14683	9.41386	9.215	10.0115	10.0887	10.322	10.2006	9.67495	6.62173	7.06071	8.61611	9.32716	7.94211	9.76275	7.73146	8.65548	7.92891	8.19586	8.81625	9.11	-0.81484	0.020183	0.157982
Potassium	KCNK6	3832292	5.45846	5.69028	5.31998	5.83304	5.62915	4.98594	5.61223	5.66351	5.90187	5.33638	5.43658	6.07713	5.93049	5.74023	5.91585	5.57025	5.55037	5.79994	6.21538	5.83344	6.41827	5.78455	6.30043	5.71717	0.319318	0.020237	0.157982
Potassium	KCNC3	3868378	6.07765	5.99374	7.10386	6.15191	6.32686	6.80882	6.36701	6.92247	7.5648	6.94921	7.67163	6.32918	5.74989	5.88837	6.81824	6.67995	5.83066	6.65024	6.02637	6.62533	6.54087	6.10658	6.07792	6.27696	-0.41631	0.021258	0.160944
Potassium	KCNA6	3401953	8.74685	8.62606	8.51044	6.77563	7.6463	7.27269	8.06484	8.12117	7.8397	7.90997	8.07937	7.43402	7.71673	8.02049	8.21834	8.05834	7.49612	7.51554	6.12154	7.22786	6.79594	6.59949	7.05702	6.83531	-0.61369	0.025882	0.174481
Potassium	KCNJ18	3714779	4.21054	4.43643	4.69056	5.3093	5.21089	5.2281	4.3923	5.17609	4.72283	4.27831	3.99881	4.29507	4.59554	4.3879	5.03912	5.75468	5.75664	5.01665	5.59089	4.89223	4.42932	4.98049	5.71064	5.61619	0.485088	0.026798	0.17661
Potassium	KCNK1	2385873	4.81435	5.37774	6.78925	7.44149	7.48198	7.85939	8.78784	8.09363	8.42582	8.49997	8.55724	8.20549	5.24725	5.45566	5.11989	7.48914	6.80608	7.94348	8.21315	8.06119	6.11943	7.81154	7.60092	7.43009	-0.58636	0.026832	0.176772
Potassium	KCNV2	3160229	3.91152	4.08216	3.99078	4.35908	5.08136	3.90377	4.11573	4.35009	4.87205	4.26044	4.35375	5.29547	4.75655	4.78524	4.31046	4.16371	5.00528	4.38498	4.94975	4.44555	5.63912	5.43114	5.04294	4.52533	0.405321	0.027206	0.178363
Potassium	KCNV1	3148963	7.39177	8.80558	8.87307	7.58168	8.85703	7.80768	10.051	8.98062	7.35341	8.33424	7.03883	8.44388	7.13418	7.61	8.32239	8.20273	7.38585	7.59808	7.20752	8.12121	7.7732	6.6569	7.54993	6.57629	-0.78171	0.028081	0.18063
Potassium	KCNJ9	2362860	5.12646	5.11401	7.58456	6.89999	6.36893	7.54458	6.32764	7.33497	7.79055	7.66058	6.80631	6.641	5.3571	7.79284	6.5059	7.39839	6.43042	7.04988	5.72298	6.57318	6.74709	5.78239	6.58179	6.63116	-0.46887	0.031625	0.191594
Potassium	KCND2	3021009	9.22493	9.3322	9.82566	7.62101	8.67702	6.98905	9.54199	9.63165	8.41833	9.19921	9.35886	9.256	7.37911	8.39236	10.0118	9.59653	8.37318	7.33498	7.89388	8.67966	6.11802	7.06496	8.39534	6.85224	-0.91532	0.032769	0.194627
Potassium	KCNJ15	3920850	3.68414	3.87722	3.99445	4.00429	3.84277	4.52958	3.86595	4.00223	3.75265	3.58865	4.2327	3.96151	3.91852	4.04886	3.69457	4.26631	4.18566	4.41789	4.51089	4.29951	5.12239	4.51927	3.85726	4.51475	0.334978	0.039622	0.21206
Potassium	KCNJ3	2511153	8.02533	7.70903	10.0372	8.85414	9.52921	7.91527	10.6413	10.2092	10.3159	10.5105	10.3729	10.3455	8.1382	5.81645	8.84476	10.0384	9.152	8.88743	9.49355	9.81049	6.43083	8.68008	9.47915	8.6654	-0.91906	0.041369	0.216165
Potassium	KCNQ1	3317352	5.08106	5.29606	5.20588	5.95714	4.93459	5.34104	4.91977	5.32952	4.81648	4.95943	5.22423	5.2471	5.53904	5.5925	5.19115	5.34105	5.59371	5.38278	5.86164	5.10791	6.29366	5.34261	5.49316	5.90596	0.361073	0.043804	0.222032
Potassium	KCNK13	3548229	5.07252	5.15526	5.42475	5.50605	5.38362	5.48564	5.38184	5.77938	5.56926	5.7566	5.8277	5.31078	5.30397	5.22714	5.49829	5.59132	5.92495	5.81962	5.77901	5.62656	6.72684	5.76599	5.47324	5.91733	0.250072	0.052054	0.237072
Potassium	KCNN1	3824666	7.95465	8.07019	9.59698	8.94447	9.65952	9.52367	9.06503	9.58652	10.0254	9.75794	8.69914	8.97837	7.39042	7.45441	8.56644	8.96669	8.82045	9.70593	9.08411	9.62377	8.74239	9.68915	9.03647	8.91626	-0.32212	0.056705	0.24661
Potassium	KCNK2	2379974	9.93713	10.4298	8.1919	7.63705	8.30001	6.84135	8.64486	7.61173	6.59881	7.31042	7.29171	6.99251	9.51144	10.7823	8.84345	6.62306	7.36436	6.72901	8.59496	6.83516	6.80736	6.87983	6.47065	6.35931	-0.3322	0.057254	0.246868
Potassium	KCNA2	2427566	5.45086	6.01602	7.28337	5.66456	6.46068	6.63883	7.63418	8.2783	8.72928	8.40902	8.3813	7.915	5.49413	6.08414	6.13001	7.21847	5.74045	6.44258	7.30722	7.92207	6.31459	5.85265	7.74339	6.9398	-0.63933	0.069662	0.268335
Potassium	KCNH8	2613293	7.16894	5.86093	5.26796	5.47834	4.72965	6.46183	4.60787	7.08096	6.90232	7.85711	6.02584	7.78793	6.08527	5.31636	5.33379	4.86477	5.09767	5.80387	6.23679	4.56626	6.9987	6.28239	5.33998	5.69133	-0.63438	0.076688	0.28021
Potassium	KCNG1	3909442	6.94194	5.80693	8.19273	7.84907	7.69286	7.14005	8.08582	7.04396	6.29069	6.77017	6.49621	5.92367	6.81023	5.84328	7.70511	8.09855	7.32066	7.22339	6.59589	7.28317	5.19079	7.11008	5.81072	5.53251	-0.30914	0.08581	0.293109
Potassium	KCNC1	3322521	7.27216	7.49133	8.38474	6.87473	7.28234	8.08656	7.87467	8.41902	9.36669	8.93058	8.25456	8.27431	7.41094	7.35075	7.58966	7.47947	7.39062	7.47421	7.74612	8.11946	8.06528	7.73225	8.13911	8.38268	-0.3026	0.093522	0.303226
Potassium	KCNQ5	2913277	10.224	10.3649	10.1589	8.9503	9.60988	8.25552	10.4055	11.0994	10.7466	11.0803	10.3249	10.91	9.37794	10.3581	9.56251	9.93637	8.78101	9.60324	10.2151	10.0091	8.1235	9.18675	10.6343	9.36106	-0.58177	0.108821	0.325924
Potassium	KCNS3	2471384	3.92421	4.41761	4.75124	5.1423	4.55525	4.48064	5.24547	5.04232	5.05343	5.11389	5.12684	5.10946	4.22037	4.4172	4.27994	4.69568	4.90273	4.48084	5.3974	4.97878	4.65914	4.23726	4.78709	4.78104	-0.1771	0.116681	0.336077
Potassium	KCNH7	2584258	8.98665	8.92019	9.64137	9.15069	10.0022	8.47106	10.3444	9.18306	9.09686	9.20419	8.54454	9.41667	9.23791	9.83449	9.36684	9.393	9.07856	9.05503	8.50868	9.10434	7.49514	8.2388	8.49229	7.98833	-0.43071	0.12572	0.34844
Potassium	KCNJ14	3837825	4.13845	4.2178	3.91418	4.64762	4.51029	4.28371	4.39041	4.2458	3.9172	4.16134	4.57889	4.39878	4.14506	4.32056	4.29606	4.35304	4.36434	4.82078	4.50089	4.21962	5.03539	4.52447	4.39794	4.58332	0.17975	0.13374	0.359692
Potassium	KCNK9	3155901	7.12914	7.8981	6.72561	5.46404	5.83201	5.28677	6.56303	6.42167	5.83894	6.25052	5.14555	6.15384	6.60623	5.79365	6.32985	5.84008	6.20614	5.16572	5.44499	6.24731	5.64947	5.4879	6.09916	5.49614	-0.36188	0.139759	0.366834
Potassium	KCNK12	2552051	7.00697	8.18371	9.36392	8.19552	9.30788	8.9486	8.49905	9.08695	8.57604	8.90364	8.66785	8.73893	6.79342	6.9728	7.93785	8.82846	8.61517	9.0907	8.77222	9.05127	9.01571	8.28914	8.48783	8.24003	-0.28204	0.151499	0.380485
Potassium	KCNK18	3266253	4.12228	4.50657	4.59478	5.03741	4.23068	4.7772	4.20001	4.68026	4.41671	4.21705	4.84122	4.97686	4.66916	4.82623	4.62756	4.68346	4.74705	4.59618	4.66993	4.41782	5.79255	4.69773	4.6359	4.8783	0.22007	0.152909	0.381715
Potassium	KCNS2	3108782	5.6604	5.28876	8.07143	7.11395	7.14295	7.00335	7.93104	7.94734	8.21879	7.91787	8.32892	7.9647	5.39036	4.65973	7.11648	7.9347	7.38688	6.55658	8.45131	8.07771	6.96784	7.60424	7.8095	7.54889	-0.25711	0.163509	0.394711
Potassium	KCNT2	2449391	7.58355	7.54459	8.70879	9.74663	9.40544	8.15629	8.66043	7.33435	7.3252	7.27275	6.67297	6.99958	8.11984	7.87844	8.6777	8.88783	9.13652	7.72018	8.05137	7.32869	6.98999	6.47142	7.02531	6.83375	-0.19079	0.171042	0.4028
Potassium	KCNN3	2436826	6.28061	6.11699	7.98987	7.66328	7.37857	8.22161	6.85579	7.54032	8.44965	7.65581	7.3554	7.93081	6.90607	5.55518	7.27295	7.66071	7.32668	8.19526	7.43742	8.51811	8.83868	7.96599	7.96408	8.2604	0.205235	0.182032	0.416258
Potassium	KCNN4	3864646	5.308	5.62945	5.71828	5.85336	5.20437	5.5423	5.33774	5.44895	5.69317	5.15469	5.75169	6.10998	5.64317	5.48275	5.72352	5.7501	5.46724	5.87025	6.07628	5.20798	6.81977	5.29113	5.54083	5.87329	0.166194	0.202151	0.43996
Potassium	KCNK16	2952927	4.04994	5.26486	4.21601	4.50187	4.52817	4.25753	3.9137	4.4477	3.97819	4.25524	4.89707	4.87382	4.65345	4.29074	4.03536	4.75252	5.11462	5.01378	4.82991	4.28806	5.26124	4.23568	4.51142	4.8903	0.224415	0.243675	0.482563
Potassium	KCNJ13	2603960	3.42682	3.70203	4.51746	4.85921	4.05756	3.9816	4.17642	4.13128	3.93724	3.67246	4.33092	3.80724	4.02643	4.81678	5.34066	4.07137	3.67794	3.97862	4.9818	4.17167	4.06276	3.89299	3.8803	4.0724	0.19779	0.253918	0.491276
Potassium	KCNK17	2952893	4.12397	4.34401	4.50707	4.64418	4.2197	5.01979	4.78411	4.6138	4.44356	4.42253	5.36686	4.68073	4.04976	4.38079	4.33335	4.6783	4.69548	5.02816	5.23285	4.74405	5.30045	4.84161	4.61489	4.88903	0.134868	0.270495	0.506714
Potassium	KCNJ11	3364869	5.68011	5.68726	5.92006	4.80607	4.92097	5.12845	4.94342	6.07298	6.34156	5.5848	5.10404	5.66828	5.35826	4.89162	5.64496	5.9688	5.7832	5.90256	5.40329	5.62032	6.55351	5.58065	5.82137	5.73742	0.200663	0.273709	0.509922
Potassium	KCNJ2	3733275	5.55384	5.41147	6.19952	5.36742	7.61937	6.71006	7.53679	8.02807	6.77026	7.53433	7.57594	8.67725	5.25258	5.43859	5.79791	6.03325	6.0819	7.68515	6.05618	7.27488	6.60793	8.26601	7.84599	7.42457	-0.26828	0.302264	0.536533
Potassium	KCNJ16	3733238	4.41125	5.00511	6.02386	6.85858	6.60781	7.95467	7.68663	6.01855	6.36557	5.22795	6.10097	6.20717	4.28375	4.42081	7.25903	7.0483	8.13022	7.16805	6.76791	7.18488	7.4259	7.44872	5.37018	5.90074	0.328364	0.307889	0.542149
Potassium	KCNMA1	3296046	8.16462	7.88639	10.1635	9.10165	9.98164	9.4228	10.0542	10.3558	10.2507	9.96659	9.71639	10.2211	8.71962	8.849	9.79875	9.85936	9.9269	9.3097	8.80221	9.79315	8.76601	9.48748	9.76023	9.42501	-0.23233	0.30902	0.543199
Potassium	KCNJ6	3931495	8.60994	8.15914	8.52092	7.95777	8.6631	7.57383	8.21832	7.98189	8.01606	7.73394	7.69295	7.78514	8.42394	7.18535	9.1351	9.60393	8.58348	9.32131	7.74755	8.61696	7.2633	7.75599	7.96334	8.32052	0.250648	0.32839	0.560414
Potassium	KCNJ10	2439960	4.82944	5.71396	9.19146	8.92869	9.38302	11.0179	10.0989	10.0037	10.9005	9.97683	10.1344	10.081	5.94338	4.41504	8.01037	9.14225	9.35167	9.57627	9.93416	9.84441	11.0222	10.0033	9.39342	10.8207	-0.23355	0.330872	0.56253
Potassium	KCNJ8	3446910	4.78114	5.8071	5.98396	4.86585	5.73697	5.92432	7.24119	5.93599	6.42251	6.71096	5.49566	6.43143	6.30362	5.33661	5.28163	5.15085	6.03211	5.72343	6.35318	6.258	5.43898	5.34793	6.07279	4.98582	-0.25434	0.340584	0.572311
Potassium	KCNS1	3907151	3.97747	4.10733	4.94269	4.33896	4.72034	4.23722	5.24402	5.35415	6.66194	6.64252	6.37294	6.8646	4.22006	4.17889	4.81379	4.54927	4.47247	4.54933	5.57986	5.03894	6.69842	5.16788	6.24035	6.49951	-0.12128	0.409925	0.632506
Potassium	KCNK10	3575241	6.45021	6.83229	5.74287	5.52924	4.9334	5.51471	5.48553	5.81567	5.52377	5.82328	5.71848	5.58214	6.9019	6.44713	6.87793	5.74306	5.67042	5.40121	5.40482	5.42759	5.88242	5.63453	5.30505	5.61243	0.113075	0.435972	0.653493
Potassium	KCNK4	3334446	4.92739	5.17517	5.39327	5.80294	5.62049	4.90421	4.64118	5.30164	5.47755	5.84208	5.55778	5.34939	4.85817	5.1295	5.42706	5.32773	5.02876	5.1687	5.4564	5.14703	5.67733	5.37174	5.07566	5.20807	-0.09308	0.436261	0.653696
Potassium	KCNG2	3795403	5.11693	5.11815	5.41198	5.57113	6.12872	5.26668	6.41847	6.49114	5.22797	5.99993	5.52449	5.17395	5.44458	5.02023	5.18816	5.81707	5.38544	5.67069	6.12684	5.70912	5.7183	5.83047	5.25439	5.17604	-0.09235	0.455287	0.668631
Potassium	KCNA10	2427538	4.18057	4.58135	4.32283	4.9484	4.28118	4.17828	4.47776	4.01498	3.87894	4.19156	3.88905	4.3265	4.16911	4.3066	4.27097	4.24533	4.38783	3.69812	4.5912	4.0307	4.26	5.04622	4.30795	5.24753	0.09918	0.494165	0.698949
Potassium	KCND1	4007689	3.81142	4.33405	5.85199	4.90604	4.88687	5.17135	5.24294	5.16559	5.24344	5.57032	5.43819	4.96415	4.34695	4.46821	6.33418	5.19458	4.96156	5.29119	5.63989	4.91089	5.62315	4.88134	4.71627	5.0362	0.068172	0.586347	0.762894
Potassium	KCNH4	3757690	4.81882	5.27858	7.2426	6.66747	6.6492	5.51466	5.52924	5.74291	6.56447	5.53293	6.09705	5.34835	4.97008	5.15665	6.55018	6.98776	6.2139	5.58318	6.08996	6.28456	5.75296	5.45046	5.53644	5.56727	-0.07024	0.612026	0.781096
Potassium	KCNJ1	3397774	3.9399	3.9078	4.02794	4.18432	4.15172	4.12751	3.82735	4.32716	3.71964	4.02026	3.81346	4.13505	4.08936	4.05293	4.15716	4.02494	3.99654	3.95871	4.22961	3.89354	4.65682	3.92961	3.48534	4.35567	0.05401	0.62191	0.788138
Potassium	KCNK7	3377737	4.19255	4.18413	4.61584	4.43997	4.53127	4.80848	4.54831	4.89469	4.24982	4.62526	5.36013	4.58619	4.35918	4.51311	4.39638	4.6177	5.07343	4.84761	4.73819	4.48334	4.98408	4.40907	4.58267	4.75106	0.059932	0.626422	0.791546
Potassium	KCNK15	3886598	5.66432	5.91218	5.61889	5.7174	6.55541	5.28463	5.97666	5.91039	5.53602	5.94351	5.5197	5.46903	5.83705	5.70463	5.79281	5.96808	5.75388	6.23388	5.78394	5.95478	5.55807	5.60014	5.76579	5.8966	0.061793	0.63096	0.794561
Potassium	KCNH5	3567873	6.56128	7.67427	8.29534	7.68839	8.15988	7.69938	9.48657	8.50003	8.27797	7.91353	8.21216	8.33514	7.31053	7.20493	9.69289	8.59712	8.04014	8.65916	7.6094	8.68509	6.83443	7.03119	7.7448	7.88708	-0.1256	0.673933	0.821987
Potassium	KCNQ2	3913821	7.59951	7.41043	8.25072	8.24573	7.7253	8.77523	7.25349	7.95952	8.60134	8.22067	7.84397	7.56773	7.56828	7.67077	7.86513	8.11404	7.77408	8.89377	7.20637	8.25534	8.20624	7.661	7.66817	8.17205	-0.0332	0.73353	0.858283
Potassium	KCNG3	2550175	4.1097	5.02003	6.61288	7.0687	7.33079	6.29449	6.93273	6.65626	6.46312	6.73748	7.2661	7.31885	4.29642	4.73461	5.79428	7.31671	7.17836	7.27946	6.57494	7.08135	6.09383	8.17881	6.46878	7.37601	0.046869	0.813696	0.902686
Potassium	KCNA7	3867669	4.12122	4.40257	3.9156	4.18012	4.33418	4.14123	4.20237	4.49281	4.00303	4.19016	4.51882	4.31663	4.21697	4.28844	4.17716	4.31586	4.57628	4.29427	4.24327	4.08175	4.53213	3.65506	4.17374	4.5255	0.021808	0.814339	0.903016
Potassium	KCNU1	3093781	3.96221	4.48437	4.6849	4.52991	4.2731	4.60671	3.91104	3.75156	3.87086	4.84154	3.93906	4.45915	4.22278	4.11971	3.93317	4.02654	4.15154	4.37905	3.999	3.70277	4.79809	3.97698	4.75508	4.89961	-0.02917	0.862094	0.928747
Potassium	KCNH2	3079202	7.0702	6.84784	7.18179	6.45699	6.73137	7.08137	6.92838	7.16262	7.20784	7.50171	7.3332	6.78653	7.67895	8.02534	6.44851	6.76581	6.67236	6.89796	7.47763	7.05435	6.55498	6.45217	7.03694	6.84091	-0.03199	0.863257	0.929258
Potassium	KCNA5	3402039	6.22138	5.89131	5.14755	5.03491	4.43532	5.04307	5.1916	4.90997	4.69501	5.61114	5.45913	5.43319	5.16045	4.90621	5.08056	5.12618	5.49717	5.65863	5.06851	4.54688	5.66738	5.793	5.14151	5.10129	-0.02715	0.890955	0.944025
Potassium	KCNJ4	3960605	6.28048	6.97726	8.61798	9.18924	7.72485	9.45809	8.6207	8.59531	9.23999	8.85277	8.91156	8.99219	6.63295	7.22251	7.8479	8.78211	8.69217	9.23124	8.49853	8.87404	9.71185	8.08357	8.88954	8.91986	-0.00618	0.967167	0.984608
Potassium	KCNA4	3367580	5.744	5.72085	6.72697	7.0446	6.73948	7.17291	7.14566	6.84036	7.21521	6.93005	7.17905	7.44638	6.29289	6.12099	7.102	7.78871	7.02267	7.34167	6.29822	6.8171	6.96387	6.98333	6.75283	6.34564	-0.0063	0.969578	0.985883
Potassium	KCNA3	2427619	8.62049	7.85939	7.62314	6.24776	8.45337	6.1157	7.47918	6.66681	7.1789	7.59004	7.43666	6.99102	8.20759	8.17151	9.36025	7.94491	7.62916	6.89983	6.74768	7.5466	5.9951	6.57026	6.97641	6.23914	0.002165	0.994396	0.997521
Potassium	HCN1	2855963	9.35342	9.95411	10.6952	8.86805	9.7325	8.38923	11.3784	11.6025	10.8457	11.3541	10.8245	11.1711	9.69588	9.75675	10.0316	10.3722	9.46156	9.66324	10.6212	10.728	7.13209	9.84289	10.7625	9.54928	-0.54597	0.198612	0.435498
Potassium	HCN2	3815045	6.10539	6.10859	6.90443	6.80081	6.74483	6.96328	5.72053	7.2461	7.37921	7.40154	7.74246	6.79794	5.96435	5.97503	6.51705	6.6181	6.44128	6.81955	6.92065	7.2954	7.04154	6.96355	7.33906	7.13631	-0.0736	0.587868	0.763784
Potassium	HCN3	2360818	7.99023	8.39632	6.38717	6.29303	6.88625	6.47457	5.99824	6.82223	6.3067	6.54177	6.38187	6.77441	8.53851	8.07536	7.36543	7.65333	6.62805	7.13087	6.81579	5.69411	7.24314	7.06579	6.66287	6.83725	0.371476	0.088822	0.29732
Potassium	HCN4	3632424	7.19238	6.924	5.92973	5.13131	5.77943	4.66591	5.25882	5.645	5.31128	5.37936	5.89767	5.6886	6.8134	6.94898	6.49272	6.12994	5.63555	5.82766	5.83521	5.65354	5.61394	5.34715	5.20335	6.0058	0.225313	0.177448	0.41036
Sodium	SCN1A	2585322	5.09245	4.34262	7.22245	7.97553	8.14429	7.48974	9.05867	8.38514	8.91581	9.03052	8.71196	9.42909	4.58631	4.46886	7.43465	7.50653	7.95859	7.76599	7.43904	7.53261	6.69966	6.11241	8.62566	8.04418	-0.80198	0.020597	0.158883
Sodium	SCN2A	2513471	10.5228	10.6503	11.3036	11.3463	11.6448	10.7064	11.3588	11.0596	10.6798	10.8556	10.818	11.2585	10.4703	10.7531	11.8115	11.421	11.2172	10.7066	10.6072	10.7416	8.20967	10.5773	10.8178	10.1496	-0.39347	0.108007	0.324602
Sodium	SCN3A	2584957	10.3917	10.3573	8.96297	9.02547	9.44213	8.13385	8.6215	7.34056	7.54933	7.42681	6.72451	7.59467	9.79416	9.97771	10.4348	8.85393	8.58329	7.89077	7.43699	7.92156	7.05312	6.78069	7.66669	6.95718	-0.18499	0.431718	0.649536
Sodium	SCN4A	3766549	4.38249	4.37222	4.39594	4.94557	4.85396	4.56573	4.35467	4.70471	4.4002	4.5038	4.52845	4.44022	4.75017	4.53033	4.42415	4.71306	4.88178	4.75601	4.99946	4.71937	4.98446	4.66119	4.70653	4.98483	0.221948	0.014354	0.140714
Sodium	SCN5A	2669641	4.34245	5.18348	5.85487	5.62962	5.19862	5.14616	5.17836	5.10002	4.95621	4.68651	5.00701	4.96833	5.03811	5.20328	5.74858	5.33378	5.16902	5.44621	5.54665	5.58481	5.76652	5.0328	5.10281	5.50342	0.268696	0.018531	0.153765
Sodium	SCN8A	3414969	8.30856	7.68418	9.77903	8.19316	8.49161	8.80149	9.96461	9.9567	9.9028	9.91984	9.11127	9.86996	8.10741	7.95535	9.44835	9.97284	8.10599	8.92645	8.18823	8.97711	8.14702	8.76793	9.18254	8.6338	-0.46418	0.136579	0.362781
Sodium	SCN9A	2585400	8.38801	8.07937	6.11842	5.78658	5.60142	5.36795	5.15894	4.56253	4.49688	4.85259	4.51106	4.51174	7.0851	8.1886	9.00124	6.20812	6.18104	5.89314	5.40822	4.83281	5.84306	5.30188	4.83964	5.39252	0.561657	0.066699	0.262857
Sodium	SCN10A	2669732	3.71665	4.45259	4.16798	4.46467	4.18817	4.39718	4.0321	4.3077	3.90551	4.13684	4.40294	4.31557	4.02351	4.16365	3.89301	3.99864	4.311	4.52961	4.28139	4.19107	4.95046	4.22058	4.17448	4.84307	0.091048	0.464868	0.676284
Sodium	SCN11A	2669803	3.78279	4.19691	4.11764	4.01916	4.05607	4.2704	4.09979	4.24076	4.07304	4.098	4.32245	4.3907	4.07729	4.41507	3.83416	4.37045	4.24818	4.37581	4.59992	4.02466	5.12993	4.14915	4.17493	4.70438	0.203018	0.07688	0.280486
Sodium	SCN1B	3830051	4.64145	5.25027	7.24494	7.52404	6.9956	7.87476	7.52753	8.78336	9.61386	9.5001	9.18483	9.00659	5.94773	6.23397	6.14325	6.7974	7.46953	7.27454	8.44075	7.93349	8.52309	7.94254	8.95263	9.218	-0.1892	0.502735	0.705162
Sodium	SCN2B	3393652	4.98951	4.96797	8.09231	7.49153	9.10493	7.48493	8.13989	9.12652	9.07657	9.02433	9.80972	8.54692	4.88082	5.27161	7.05086	8.17448	8.54269	8.70765	7.72232	8.89647	6.73839	8.61786	9.16426	8.79084	-0.27474	0.312606	0.54619
Sodium	SCN3B	3395691	10.9775	10.4077	11.4502	10.4856	11.3461	10.2333	11.3363	11.1744	10.6977	10.9056	10.7745	10.6679	10.4305	10.5432	11.2048	11.0493	10.0693	10.458	10.669	11.105	8.04298	10.5616	10.2061	9.20152	-0.57629	0.044024	0.222553
Sodium	SCN4B	3393622	6.35224	6.13634	7.22367	7.20383	7.09261	6.69986	7.50278	7.58036	8.48693	8.26724	7.94237	7.86816	5.81188	5.77145	6.93853	7.30083	6.89573	6.69555	7.50358	7.99323	7.46216	7.34274	7.92963	8.41324	-0.19149	0.190548	0.426437
Glutamate	GRM1	2929571	6.9646	7.66599	7.88528	6.06117	6.91248	5.92181	6.96374	6.58456	6.17401	6.46173	6.2542	6.82959	6.31541	6.15232	8.00567	7.15371	6.63151	6.51451	7.01186	7.02115	5.8387	6.6571	6.58342	6.04399	-0.06248	0.762578	0.874728
Glutamate	GRM2	2623308	7.78965	7.46343	7.30547	6.8592	7.46776	7.09161	7.82568	8.21332	6.81237	7.5979	7.44641	7.03506	8.14434	7.97804	7.1066	7.65768	5.47051	7.12179	6.88763	7.32461	5.3747	6.0755	6.45724	5.47796	-0.65261	0.032535	0.193731
Glutamate	GRM3	3011180	9.74173	10.1592	9.9968	10.1394	10.1856	11.2244	10.5459	10.2972	10.3916	10.0238	9.78729	10.1139	10.2006	10.2335	10.1702	9.79147	10.4848	10.2267	9.98797	9.86444	9.39486	9.75358	9.33279	9.38808	-0.31482	0.045087	0.224119
Glutamate	GRM4	2950970	6.54535	6.50557	6.94835	5.9122	6.51156	6.07749	6.16747	6.90883	6.04156	6.61541	7.14051	6.68577	6.82759	6.5307	6.62747	7.10177	6.47441	6.77802	6.89038	6.51252	7.04858	6.47718	6.39521	6.76149	0.197104	0.275738	0.51165
Glutamate	GRM5	3385834	10.0207	9.84068	10.726	8.85442	9.54661	9.14863	10.4384	9.77156	9.8191	9.70718	9.68514	10.0075	9.7842	9.51233	10.9225	10.4948	9.99087	9.77466	9.16089	9.85892	8.80281	9.35967	9.91232	9.78563	-0.01719	0.939036	0.96939
Glutamate	GRM6	2889848	4.30041	4.85992	4.38101	4.59965	4.46797	4.39557	4.39672	4.51018	4.12381	4.78546	4.49877	4.4059	4.33079	4.90699	4.31605	4.60515	4.28005	4.7664	5.11363	4.17771	4.89822	4.50723	4.51261	4.99747	0.140578	0.227246	0.46511
Glutamate	GRM7	2609061	9.84873	9.67059	8.89439	8.8615	8.23802	7.96431	8.2186	7.24969	7.6388	7.46705	7.68894	6.78426	7.42308	8.63918	9.6062	8.86337	8.49004	7.86963	7.0413	7.80061	6.61144	6.85507	6.24304	6.36922	-0.55939	0.057153	0.246761
Glutamate	GRM8	3071063	3.96692	4.30785	5.53899	6.11841	5.45035	5.22925	6.00485	6.06567	5.92758	6.16355	5.90578	5.90832	4.16212	4.99274	5.08245	5.86418	5.13825	6.08603	4.84952	5.35511	4.61438	5.17809	5.38301	5.42743	-0.37118	0.082889	0.288985
Glutamate	GRIK1	3928211	4.31625	4.64284	8.08866	8.2507	9.07786	6.97122	7.53415	7.66934	7.41725	7.49234	7.58118	7.21589	5.51478	4.71849	7.47732	9.36772	9.26277	7.88216	8.16258	8.78608	7.1632	7.3485	7.0451	6.99354	0.288713	0.165706	0.39739
Glutamate	GRIK2	2918982	10.1621	9.61731	8.7164	9.94105	9.44504	9.07282	8.27331	8.56561	9.02951	8.71417	8.28433	8.58209	9.11004	8.43402	9.53687	9.30805	9.53007	8.78975	8.71052	8.55479	7.82582	8.68958	8.32998	8.54137	-0.25357	0.198872	0.435579
Glutamate	GRIK3	2406926	10.1052	9.15836	9.97605	8.40295	7.96809	8.49264	8.67691	8.67644	8.65685	9.00167	8.81143	8.08814	10.411	9.42466	8.97416	8.15813	8.19844	8.14684	7.22883	7.67089	6.76241	6.76452	7.45928	6.50194	-0.88196	0.004334	0.096319
Glutamate	GRIK4	3352618	7.27962	7.12795	8.43943	7.80409	8.16993	8.44398	8.82329	7.98322	8.23127	7.97187	8.18898	8.36823	6.13846	6.35946	7.83931	7.56769	8.27987	8.23591	7.82709	7.78749	6.65364	8.3401	7.58998	7.76225	-0.53755	0.005813	0.107475
Glutamate	GRIK5	3863380	9.11596	8.51005	9.25841	8.51467	8.22999	9.15891	7.92031	8.00354	8.72673	7.90862	8.06568	7.63386	8.90341	8.83718	8.98477	8.55566	8.00198	8.4132	7.5379	8.35606	8.11802	8.33519	8.04697	8.10729	-0.07076	0.559441	0.74432
Glutamate	GRIA1	2836242	11.0211	10.8211	10.6809	10.3538	10.6996	9.35001	9.94894	10.3305	9.95932	10.097	9.82156	9.74576	10.2026	10.4579	11.2085	10.8007	10.8006	9.97197	9.34459	9.9673	7.02357	9.16141	9.44774	8.84882	-0.46616	0.118504	0.338724
Glutamate	GRIA2	2749222	10.9234	10.2847	11.2441	11.8872	11.378	11.352	11.1845	11.1646	11.1604	11.1467	11.0234	11.1356	11.1458	10.8499	11.2804	11.4459	11.6987	11.3425	10.4819	10.1909	9.94699	10.7385	10.9067	10.6878	-0.21518	0.170023	0.401546
Glutamate	GRIA3	3989448	9.56881	9.82365	10.1694	9.14236	9.84165	9.24381	9.81427	10.9293	10.1323	9.88356	9.5027	9.84392	10.5841	9.59795	10.4513	9.65947	9.19341	9.26615	8.70077	9.64087	7.73303	9.16492	9.68172	9.25148	-0.41421	0.14784	0.376779
Glutamate	GRIA4	3347118	7.48708	7.01593	8.7413	8.78399	9.06976	7.64798	8.69252	8.65511	8.44519	7.94484	7.69603	8.19854	7.02921	7.06859	8.80103	9.27678	8.51563	7.83438	7.93177	8.11026	6.39428	7.82935	7.22171	7.35107	-0.41785	0.049723	0.232894
Glutamate	GRIN1	3195238	7.33534	7.40386	8.53439	7.89631	7.79487	7.47043	7.86017	8.25287	8.6011	8.43387	8.46537	7.68949	7.05255	7.14304	8.10018	7.91445	7.69116	7.71437	7.29537	8.02881	7.94499	7.62215	8.02448	7.5334	-0.30609	0.004251	0.095684
Glutamate	GRIN2A	3679812	6.10714	6.77851	10.3608	9.36816	9.13758	9.69644	10.5161	9.75684	9.42188	9.723	8.63459	9.76603	4.92043	5.93043	8.92859	9.70293	8.46645	9.14424	8.69894	9.20708	8.11799	8.37273	8.64369	8.54121	-0.8827	0.000489	0.049618
Glutamate	GRIN2B	3445326	11.0904	11.4254	11.4836	9.66821	11.0694	10.205	10.9001	11.3196	10.1423	10.2118	9.53379	10.7597	11.7454	11.5557	11.6166	10.9755	9.84527	10.1913	8.36598	10.2654	7.6352	8.42587	9.2817	9.14249	-0.73024	0.066416	0.262333
Glutamate	GRIN2C	3770422	5.28043	5.20397	6.03956	5.82734	6.65948	7.03079	5.78644	6.11076	7.01796	5.84011	6.52767	6.09869	5.30061	5.31528	5.80155	5.98512	6.01609	6.28033	6.06948	6.84161	6.79688	6.1762	6.50968	6.57663	0.020522	0.87284	0.934076
Glutamate	GRIN2D	3837759	5.74182	6.009	5.66749	5.42231	5.59601	5.2319	5.22584	5.69406	5.52036	5.76351	5.4437	5.53933	5.92869	5.94012	6.44239	5.78772	5.58031	5.78348	6.00137	5.58462	5.80856	5.6282	5.80355	6.29156	0.310437	0.010276	0.127165
Glutamate	GRIN3A	3218151	5.88717	5.21065	9.35098	8.45349	7.51908	7.41287	7.8673	7.18527	7.14011	6.82792	6.75276	5.95	5.60416	5.3358	9.94109	9.80521	8.88631	8.52325	7.18901	7.44969	5.72729	6.94437	5.69899	7.18136	0.227411	0.42441	0.643913
Glutamate	GRID1	3298557	7.53386	7.47144	7.99477	7.52231	6.5954	7.91308	7.65355	7.72807	7.61686	7.92873	7.54701	7.90629	7.8604	7.37144	8.01985	7.57939	7.28884	7.35694	6.48053	6.9917	6.99688	6.86205	7.14014	7.54162	-0.3268	0.065438	0.261523
Glutamate	GRID2	2736060	7.99446	8.31885	8.14918	8.74224	7.58931	7.884	7.78355	7.25176	6.85949	7.29675	6.56081	6.61633	7.68551	6.54832	8.45185	7.96404	8.86142	7.6525	7.47567	7.19915	6.23597	7.83941	6.46046	7.01234	-0.13834	0.540068	0.730675
Glutamate	SYP	4007899	9.05091	8.23396	11.5858	11.2078	11.8251	11.4516	10.3238	11.6055	11.3452	11.5414	11.9851	10.5678	8.24211	8.55675	11.0838	11.8957	11.9674	12.012	11.3855	11.2921	11.6839	11.399	10.8795	12.0837	0.146458	0.52098	0.718941
Glutamate	SYN1	4007126	8.82551	9.31361	11.0638	10.6514	9.95353	9.99878	10.4523	10.6464	10.83	11.0132	10.7208	10.4692	8.45257	8.6494	10.1122	10.3523	9.69248	10.2778	10.5806	10.6166	8.721	11.0812	10.539	10.4761	-0.36561	0.076567	0.279942
Glutamate	DLG4	3743393	9.83209	10.0544	11.1888	10.6702	11.3336	10.9283	10.1639	11.5077	10.7324	10.9642	11.0162	10.5422	9.8524	9.66243	10.3498	10.6474	10.2864	10.6969	11.2084	11.0234	10.0988	10.9105	10.5785	10.2345	-0.28205	0.091433	0.300729
Glutamate	NLGN1	2652801	9.91758	9.80807	10.0697	8.70537	10.0502	8.1761	10.55	9.6438	8.73988	9.16096	8.31006	9.74758	11.1957	10.0118	10.4631	9.81776	9.88244	8.52626	9.00654	9.20337	6.94752	8.11668	8.28663	7.46698	-0.32954	0.33326	0.565046
Glutamate	SLC17A7	3867842	7.69406	8.51846	12.3159	11.8199	12.3213	12.0996	11.9806	12.3116	12.303	12.2752	11.7531	12.0439	8.40037	8.03974	11.0339	11.813	11.752	12.0008	11.8462	11.9719	11.6003	11.9919	11.7127	11.3328	-0.32842	0.039978	0.212425
Glutamate	SHANK1	3868587	7.65944	7.84441	9.59671	9.09994	9.21904	10.0467	8.60386	9.32814	9.53698	8.75027	8.865	8.5757	7.42654	7.95086	8.53623	9.41365	8.50856	9.54224	8.37642	9.42078	9.06288	9.17782	9.04077	8.7346	-0.16124	0.243541	0.482492
Glutamate	SHANK2	3380365	8.35048	7.95664	9.65794	8.56271	8.84246	9.14096	8.60743	8.34782	8.84867	8.32895	8.82146	7.71966	8.20242	7.75536	9.24505	8.8448	9.13434	8.879	7.48901	8.54022	8.00916	7.81353	7.81235	8.15686	-0.27526	0.096321	0.307035

Supplementary data 3 – Expression of K$^+$ channel, HCN channel, Na$^+$ channel and glutamatergic synapse genes in human cerebellar cortex across development and adulthood

Group	Gene	ID_REF	Con_CBC_	Con_CBC_	Con_CBC_	Con_CBC_	Con_CBC_	Con_CBC_	Con_CBC_	Con_CBC_	Con_CBC_	Con_CBC_	DS_CBC_S	DS_CBC_S	DS_CBC_S	DS_CBC_S	DS_CBC_S	DS_CBC_S	DS_CBC_S	DS_CBC_S	DS_CBC_S	DS_CBC_S	Log2FC	pvalue	FDR
Potassium	KCNF1	2469627	4.90699	4.68351	4.76334	4.45581	4.31699	4.55371	4.78726	4.68047	4.58972	4.64983	4.98839	5.1735	4.38568	4.94143	4.59595	4.55785	4.75497	4.51958	5.11389	4.67684	0.132045	0.20265	0.496337
Potassium	KCNC4	2351294	6.58257	6.23309	5.87348	6.19427	6.4605	5.95529	6.49353	6.58516	7.06777	6.79616	5.68436	5.56505	5.74179	6.05695	6.12598	5.93098	6.1393	6.16104	6.04274	6.5079	-0.42857	0.002853	0.096143
Potassium	KCNK3	2473936	8.82651	8.3614	7.18957	8.23342	7.11408	7.62961	7.3803	7.35489	7.29037	7.60356	6.54458	6.61848	7.58922	6.69164	7.2369	6.72259	6.15597	6.45412	5.71981	6.26681	-1.09836	0.002266	0.090195
Potassium	KCNH1	2454140	8.69404	9.01562	9.8337	9.11538	9.24057	9.33208	9.18692	9.09908	9.1387	8.49883	6.64692	8.97448	8.80529	8.6456	9.22643	8.50499	8.67706	9.09727	8.83685	8.56867	-0.51714	0.033603	0.229357
Potassium	KCNQ3	3154002	6.77415	6.22844	6.95772	7.32634	7.13384	6.9323	7.65367	6.46727	6.81283	6.39623	7.8075	6.88864	7.68408	6.47972	6.67226	7.56337	7.20317	5.84552	6.03698	6.40974	-0.00918	0.968321	0.986451
Potassium	KCNK5	2952834	5.11625	5.38704	4.91242	5.10537	5.07843	5.05877	4.6204	5.17046	5.30304	4.97748	5.20886	5.26494	4.91993	5.44468	5.24648	5.56482	5.11403	5.30597	5.51122	5.76461	0.261588	0.013877	0.162229
Potassium	KCNC2	3462567	7.12577	6.82069	6.8185	5.59837	4.9048	4.96953	5.67244	5.31924	6.30229	6.02853	7.78324	7.78416	7.2656	5.41054	5.21841	5.21276	4.92354	5.29715	5.34779	5.09516	-0.02218	0.91943	0.966288
Potassium	KCND3	2428119	9.36973	8.89971	9.46858	9.78813	9.31737	10.2561	10.0912	9.73963	9.90285	8.49922	8.61195	8.82663	9.89914	8.53808	9.38155	9.03038	8.93472	8.68182	7.81024	8.4567	-0.71613	0.018011	0.179246
Potassium	KCNB2	3103062	7.01051	6.35234	6.04279	4.36131	4.07367	4.11924	3.88931	4.34188	4.14126	4.88344	8.49581	6.79366	7.68132	4.23215	4.43167	4.63399	4.53009	4.93099	4.97731	4.75709	0.624833	0.008044	0.13204
Potassium	KCNA1	3401994	10.2306	7.5291	10.4117	11.2542	10.7481	10.8527	11.4883	10.9711	11.2626	10.5149	8.56903	9.83936	10.0285	9.84118	10.5364	9.6221	10.5831	10.3777	7.91669	9.4864	-0.84628	0.091477	0.347851
Potassium	KCNN2	2824581	7.271	7.88147	7.52272	7.31379	7.84806	7.4026	7.51864	6.56174	7.11497	6.47708	6.94256	6.44432	6.96507	6.63482	7.00958	5.82912	6.49429	6.58274	6.84685	6.39164	-0.67711	0.003445	0.101599
Potassium	KCNH3	3414029	5.90983	6.05209	6.23359	6.43514	6.44227	6.10283	5.64813	6.11443	6.01493	6.01623	6.15323	5.99355	5.98238	6.11922	6.03475	5.98682	6.3344	6.44276	6.89176	6.24168	0.121108	0.395317	0.668142
Potassium	KCNJ5	3355860	4.29597	4.63337	4.73035	4.50473	4.36518	4.28379	4.20929	4.34076	4.07487	4.54462	4.43553	4.42762	4.37396	4.72616	4.63121	4.58073	4.61983	4.6946	4.98678	4.53629	0.202978	0.100886	0.363038
Potassium	KCNQ4	2332091	5.21985	6.05541	5.72353	5.46733	5.29777	5.847	5.26759	5.89373	5.65184	6.2311	5.58251	5.67312	5.53862	5.58237	5.54287	5.68993	5.86123	5.75812	6.0058	6.21646	0.079588	0.432073	0.698422
Potassium	KCNH6	3730698	5.10102	5.36072	4.94837	5.27615	5.22589	5.03587	4.86999	4.9624	4.75789	5.16073	5.2022	5.26828	4.89058	5.19373	5.00475	5.20478	5.18524	5.24743	5.43982	5.35761	0.129539	0.153894	0.436814
Potassium	KCNG4	3702429	3.70588	4.55488	4.42254	4.30784	4.48108	4.45627	4.16421	4.30435	4.23454	4.62387	4.38707	4.28526	4.39545	4.49393	4.36609	4.07595	4.50121	4.63337	5.01423	4.70526	0.160236	0.216752	0.511701
Potassium	KCNB1	3908901	5.8013	5.22304	5.32394	6.9119	6.72357	5.92272	6.41202	6.68384	7.65395	7.55412	6.35809	5.85952	5.85406	6.57264	6.94951	7.27702	6.74788	6.83172	6.21061	6.89218	0.134283	0.599273	0.802857
Potassium	KCNK6	3832292	5.16115	5.80347	5.83381	5.63054	5.93761	5.76997	5.63154	5.57567	5.17054	5.86595	6.30971	5.85284	5.1909	5.95933	5.70971	5.61328	5.75895	5.69841	5.83833	5.6737	0.122491	0.461618	0.718432
Potassium	KCNC3	3868378	8.74718	7.89029	8.22209	8.55341	8.53724	8.68139	7.64179	7.95606	8.13463	8.99285	8.00009	8.58459	8.33821	8.22052	8.14992	8.65538	8.12757	7.26869	7.79393	7.5474	-0.26706	0.209614	0.503532
Potassium	KCNA6	3401953	6.92032	5.79929	5.76354	6.50446	5.77751	5.84432	6.10953	5.57074	5.42583	5.88637	6.98644	6.50264	6.35945	5.66256	5.70853	5.72894	5.7069	5.46216	6.01953	5.88791	0.042315	0.787509	0.905918
Potassium	KCNJ18	3714779	4.95519	5.00077	5.55956	6.03398	5.89271	5.97148	6.36389	5.88768	6.01137	5.09919	4.0593	5.20241	5.87554	6.0493	6.09068	6.27221	5.73119	5.316	5.81775	6.00399	-0.03575	0.839429	0.92957
Potassium	KCNK1	2385873	10.4477	10.1768	10.1101	10.3512	10.3745	10.2397	9.93577	9.94519	10.2379	10.0195	9.01659	10.0393	10.1664	9.86272	10.3065	9.82751	9.97008	10.1262	8.87148	9.7809	-0.38707	0.060974	0.291904
Potassium	KCNV2	3160229	4.65126	4.88627	5.07678	4.91488	4.74609	4.83619	5.14507	5.00346	4.62417	5.42296	4.80189	5.59304	5.02223	4.9575	5.24261	5.72823	5.35599	5.17854	5.28362	5.31246	0.316898	0.018083	0.179576
Potassium	KCNV1	3148963	4.79117	4.89958	4.95944	5.15353	4.40568	3.95645	4.63817	4.82442	4.34503	5.2843	5.15731	4.95349	4.76008	5.13778	5.05776	5.42648	4.99231	5.27759	4.87321	5.18026	0.35585	0.045488	0.261417
Potassium	KCNJ9	2362860	6.94482	7.09372	7.62151	8.35532	8.40587	8.39836	8.22466	7.78992	8.01496	8.00421	5.80252	7.06118	8.28723	6.99794	7.32052	6.78676	7.46174	7.35026	7.10099	6.79148	-0.78927	0.005355	0.117369
Potassium	KCND2	3021009	10.3899	9.4635	10.4935	11.356	11.0402	11.5025	11.4547	11.7113	11.7939	10.7584	10.4762	10.3482	10.7567	10.1226	10.8301	10.2913	11.0589	11.0512	9.40683	11.0634	-0.45585	0.165215	0.451468
Potassium	KCNJ15	3920850	3.77201	3.58761	4.64818	3.75971	4.43808	3.81245	3.75365	3.86018	3.78785	3.92822	3.98351	3.76332	3.80535	4.07197	3.55816	3.96678	4.07055	4.38249	4.75485	4.39584	0.140488	0.46213	0.718726
Potassium	KCNJ3	2511153	10.1765	9.88677	10.4083	10.944	11.0964	11.1609	11.2276	10.757	11.3165	10.7013	10.3544	10.4667	11.3343	10.7483	11.103	10.5802	11.2374	10.937	10.0445	11.2229	0.035343	0.861949	0.940209
Potassium	KCNQ1	3317352	5.01493	5.86213	5.20705	5.35676	5.1472	5.17981	5.39666	5.2877	4.89443	5.29896	5.39653	5.1169	4.98213	5.50022	5.20069	5.56636	5.24031	5.38757	5.62078	5.33436	0.070022	0.593187	0.799154
Potassium	KCNK13	3548229	5.13903	5.70269	5	5.41619	5.33079	4.85506	5.44128	5.46855	5.04072	5.24355	5.0724	5.09312	4.84025	5.47503	5.33819	5.19989	5.27654	5.39525	5.64342	5.34031	0.003654	0.972094	0.987373
Potassium	KCNN1	3824666	8.88585	7.69987	6.6167	8.04688	7.65737	7.46506	6.9829	7.72354	7.77285	7.69159	6.98895	8.20305	7.01985	7.78271	7.33467	8.10299	7.10313	6.06184	6.09192	6.43489	-0.54186	0.117865	0.390001
Potassium	KCNK2	2379974	8.15953	6.25953	6.72625	5.78569	5.29556	4.46186	5.22061	4.87438	4.37771	4.43023	8.35656	7.58495	7.13212	5.76542	5.76068	4.90473	5.74052	4.65511	5.16483	4.92129	0.439486	0.009606	0.141374
Potassium	KCNA2	2427566	7.49631	6.98088	8.14016	8.26107	7.78075	8.29649	8.66575	8.45794	8.18148	8.51476	5.69662	6.79755	8.18888	7.31485	7.53309	7.87885	8.275	7.60658	6.76796	7.83749	-0.68787	0.004469	0.109575
Potassium	KCNH8	2613293	7.33053	8.71181	8.6156	8.83085	8.40745	7.73076	6.50315	5.79118	5.53474	5.40732	6.0225	6.21343	8.07938	8.12167	5.71262	7.06737	5.6271	4.50529	5.44919	4.63552	-1.14293	0.002039	0.087647
Potassium	KCNG1	3909442	4.53297	5.25463	5.08093	4.83195	5.05237	5.3902	5.16614	5.14315	5.47711	5.5429	4.24762	5.01342	4.77015	5.38849	5.93445	5.58699	5.7189	4.99743	6.04498	4.97072	0.12008	0.461665	0.718441
Potassium	KCNC1	3322521	10.0325	10.1697	9.54555	10.3554	10.5422	10.027	10.0905	10.0691	10.2304	10.0685	8.08024	9.35767	9.99282	9.26733	9.59157	9.6757	9.7275	9.11449	9.26264	9.40313	-0.76578	0.003527	0.102197
Potassium	KCNQ5	2913277	5.43038	5.38244	5.50082	5.68998	4.72395	5.00697	5.36986	5.13615	5.55669	6.03289	4.78442	5.51985	6.48645	5.08775	5.38904	5.47767	4.7214	5.10522	4.75479	6.08347	-0.04201	0.835684	0.928144
Potassium	KCNS3	2471384	3.9009	4.45906	4.14981	4.23639	4.15279	4.02782	4.19614	4.22358	3.85845	4.35815	4.1408	4.49052	3.88551	4.13685	4.12716	4.53139	4.08163	4.20692	4.27263	4.39528	0.07056	0.381428	0.657703
Potassium	KCNH7	2584258	8.62619	8.7461	9.55498	9.22592	8.93802	9.24783	9.30523	7.2067	7.8273	7.51668	8.88198	8.84014	10.1779	8.66399	8.83172	8.98071	7.90998	7.59052	6.25105	8.14404	-0.19229	0.455145	0.7138
Potassium	KCNJ14	3837825	4.21324	4.40763	4.5305	3.86522	4.33415	4.34019	4.09616	4.19418	3.9615	4.25894	4.28576	4.42954	4.2067	4.4785	4.55408	4.6624	4.43115	4.70076	4.37211	4.19808	0.211737	0.043427	0.255823
Potassium	KCNK9	3155901	8.35154	7.29319	8.30851	8.7153	7.66299	8.32366	8.39594	9.27022	8.29297	6.89632	8.23211	8.242	8.09443	8.09895	8.377	7.56847	8.55653	8.1774	7.02978	8.28347	-0.08505	0.768728	0.896397
Potassium	KCNK12	2552051	8.84235	8.56956	9.16427	9.44552	9.08092	9.2354	8.88416	9.49925	9.11341	9.36094	8.37838	8.80975	8.89079	9.0942	8.94534	9.28748	8.76726	8.91158	8.87812	9.42184	-0.1811	0.050889	0.27195
Potassium	KCNK18	3266253	4.33634	4.94408	4.29019	4.53553	4.59318	4.40513	4.41322	4.49638	4.19875	4.50565	4.60922	4.19369	4.30619	4.84279	4.37111	4.50506	4.6089	4.66699	4.71464	4.64563	0.074577	0.514845	0.753045
Potassium	KCNS2	3108782	5.09042	5.95011	4.54898	5.32391	5.488	5.11704	4.55152	4.81142	5.71012	6.46735	5.4618	5.20058	4.5829	6.13008	5.31346	5.01233	5.08981	5.13201	3.99314	5.18462	-0.19581	0.467425	0.722567
Potassium	KCNT2	2449391	5.38719	5.12875	4.68191	4.15352	4.36268	4.45244	4.09174	4.04412	4.14922	4.79613	6.45568	4.85229	6.02249	4.34702	4.49463	4.58273	4.24308	4.49146	5.2062	4.66545	0.411333	0.043156	0.25546
Potassium	KCNN3	2436826	6.45831	6.55337	5.93919	6.59174	6.38264	6.62443	6.46782	6.56955	6.09548	5.66287	6.87143	6.23386	6.58154	6.6826	7.26659	7.48682	7.02498	7.64685	7.1942	7.47862	0.712209	0.004063	0.10541
Potassium	KCNN4	3864646	5.29989	5.82525	5.69905	5.50552	5.51174	5.2962	5.6686	5.43014	5.40011	6.07838	5.62789	5.59932	5.53519	5.66774	5.46029	6.00176	5.70555	5.87058	5.64219	5.53846	0.093409	0.43389	0.699666
Potassium	KCNK16	2952927	3.8396	4.48999	4.9044	4.59791	3.88463	3.90748	4.02171	4.54492	3.75779	4.32526	4.81064	4.07497	3.97206	4.47512	4.44073	4.38007	4.60355	4.9325	4.5427	4.8923	0.285095	0.161611	0.446333
Potassium	KCNJ13	2603960	4.26312	3.97099	3.56598	3.57095	3.70371	3.74596	3.84618	3.89794	3.55861	4.22612	3.9648	3.40884	3.55983	4.08886	3.96862	4.21288	4.67758	3.89781	4.02043	4.15676	0.160685	0.260616	0.55408
Potassium	KCNK17	2952893	5.35736	5.13311	4.42791	4.66587	4.92498	4.13709	4.76484	4.87401	4.11832	4.8349	4.86812	4.36762	4.27336	4.89388	4.65439	4.43713	4.83161	4.34873	4.70603	4.83449	-0.1023	0.460605	0.717683
Potassium	KCNJ11	3364869	6.7329	4.95488	6.83085	7.57191	7.32391	7.26736	7.82772	7.58469	7.64472	6.3427	5.74312	6.64962	7.44138	6.46663	6.99152	7.4653	6.57751	6.17977	6.73408	6.30018	-0.35325	0.28564	0.576634
Potassium	KCNJ2	3733275	6.48134	5.89707	6.60178	5.92403	4.99916	4.7851	5.33375	6.37021	5.47509	5.32216	7.75995	5.46953	5.4019	5.94447	5.30594	6.75509	5.05093	5.10593	6.2985	5.53664	0.143919	0.667065	0.843055
Potassium	KCNJ16	3733238	5.28062	5.38124	5.80792	6.6992	5.66657	7.7337	7.86875	7.22208	7.98491	6.23974	6.17835	6.57946	5.75568	7.17374	8.42747	7.08716	5.68832	7.74801	8.51654	7.85583	0.512583	0.252366	0.546673
Potassium	KCNMA1	3296046	9.10534	8.43896	7.59709	9.56487	8.27073	8.98848	9.2373	8.9269	9.01469	9.27757	7.92915	9.23202	7.71052	9.30837	9.1548	9.83808	9.2059	8.48807	8.12079	8.87325	-0.0561	0.811962	0.916537
Potassium	KCNJ6	3931495	7.76906	7.04484	8.56799	8.37765	7.72983	8.1939	8.13105	7.23715	7.13794	6.28591	9.34668	8.70086	9.41497	8.23734	8.72416	8.86945	8.17973	8.45803	7.45558	8.25011	0.916159	0.002637	0.095186
Potassium	KCNJ10	2439960	8.38991	7.85549	7.49367	9.26231	8.99043	9.472	9.30443	9.671	8.98284	8.98406	7.95514	7.43002	7.37636	9.18194	9.38882	9.48592	8.67476	8.91469	9.84063	8.95892	-0.11989	0.453758	0.712973
Potassium	KCNJ8	3446910	5.5321	4.27017	4.93848	5.0103	5.16901	5.03188	5.47133	5.38032	5.05423	4.6676	5.18265	5.52353	5.00453	4.91584	5.14809	5.41058	4.98781	5.12093	5.61264	5.20365	0.158483	0.366423	0.645881
Potassium	KCNS1	3907151	3.61529	3.96489	3.69995	3.84943	3.86131	3.6382	3.8854	3.83822	3.70669	3.67251	4.39619	3.82554	3.74683	4.11157	3.656	3.90173	3.83026	3.88744	3.98989	3.96624	0.15798	0.114184	0.38412
Potassium	KCNK10	3575241	5.83858	6.66131	7.34493	6.98894	6.59831	7.38099	7.16669	7.08854	6.21241	5.3792	6.16344	5.44726	6.33443	6.13897	6.75811	6.11905	6.24098	6.75631	5.94345	6.30919	-0.44487	0.088017	0.342097
Potassium	KCNK4	3334446	4.57982	4.86738	5.86819	5.16789	4.95839	4.97013	5.11842	5.21006	4.86675	5.06325	4.98791	4.68107	5.06233	5.05243	5.13619	5.13379	5.37628	5.37353	5.31098	5.38337	0.08276	0.499772	0.743349
Potassium	KCNG2	3795403	4.93737	5.1448	5.36854	5.00956	5.56561	5.56452	5.07254	5.17626	5.35592	5.53184	5.78612	5.03884	5.52388	5.26437	5.36678	5.82656	5.02384	5.07185	5.29109	5.49164	0.095801	0.351655	0.635264
Potassium	KCNA10	2427538	4.06378	5.33271	4.59406	4.3103	4.5916	4.37836	4.32968	4.3136	4.53848	4.39775	4.52892	4.26727	4.17543	4.81831	4.25156	4.69976	4.45831	5.03574	4.8948	4.92023	0.120001	0.513242	0.752599
Potassium	KCND1	4007689	6.8094	5.96141	7.51795	7.42529	7.42526	7.37759	7.0135	7.13653	7.24316	7.82272	5.49344	6.76092	7.87067	6.08533	6.94389	6.27478	7.22946	6.06182	6.61593	5.85143	-0.65451	0.042763	0.254244
Potassium	KCNH4	3757690	4.37548	4.88552	4.86829	4.76711	4.90906	4.53689	4.79828	4.80636	4.45266	4.79664	4.85875	4.91468	4.86343	4.83089	4.78499	4.7551	4.80826	4.80559	5.20224	4.94689	0.157453	0.094314	0.352465
Potassium	KCNJ1	3397774	3.8211	4.09562	4.16417	4.24877	4.16877	4.07251	3.69409	4.12107	3.98377	3.95114	4.09468	3.72122	4.05839	4.01876	4.07774	4.22714	4.32678	4.09587	4.09788	4.04418	0.044163	0.631996	0.822263
Potassium	KCNK7	3377737	3.95928	4.3349	4.88395	4.26148	5.03334	4.73307	4.50169	4.10348	4.60989	4.95515	4.11767	4.29282	4.52898	4.26521	4.13521	4.39512	4.26214	4.73283	4.50898	4.20602	-0.19313	0.196561	0.489436
Potassium	KCNK15	3886598	5.2519	4.87994	5.58809	5.61194	4.75902	5.17434	5.58475	5.97667	5.19506	4.69968	5.94815	6.18041	5.50379	5.81827	5.64943	5.83913	6.07618	5.94743	5.60165	5.79623	0.563928	0.003669	0.104194
Potassium	KCNH5	3567873	3.69901	4.05123	3.82235	3.81558	3.65466	3.68948	3.78913	3.73795	3.69964	4.2849	3.84008	3.75557	3.56301	3.81633	3.95997	4.08756	3.93446	3.86458	3.87251	3.84091	0.029105	0.745769	0.88448
Potassium	KCNQ2	3913821	7.54558	7.75548	8.12485	7.42943	7.92675	8.20139	7.69432	7.41344	7.62545	7.72078	7.28994	7.47531	7.97143	7.06634	7.36354	8.05716	8.0314	7.20725	8.06937	7.23169	-0.1674	0.13756	0.41575
Potassium	KCNG3	2550175	3.83126	4.77727	4.67847	4.23353	4.25285	4.05354	3.89117	5.4027	4.08388	4.50593	4.41822	4.26617	3.93443	4.12722	3.93031	4.46562	3.98717	4.29119	4.14921	4.60969	-0.15314	0.379374	0.656094
Potassium	KCNA7	3867669	4.32459	4.22531	3.99339	4.5388	3.99628	3.95966	4.19874	4.51414	4.08552	4.23674	4.48056	4.30949	4.28086	4.32497	4.11116	4.39638	4.0944	4.25537	4.33289	4.51555	0.102846	0.190406	0.481338
Potassium	KCNU1	3093781	3.8819	4.67182	4.4511	3.84397	4.38926	3.96386	3.97613	3.96952	4.20224	4.94089	4.46948	4.17658	4.00761	3.90001	3.72811	4.85247	4.37484	4.17254	4.56311	4.22271	0.017677	0.923185	0.967086
Potassium	KCNH2	3079202	7.82256	7.7224	7.6466	7.63191	7.8841	7.77671	8.07502	8.15249	8.50425	8.42969	7.17825	7.43391	7.60597	7.56608	7.38569	7.34987	7.56087	7.49621	7.21604	7.66128	-0.51916	0.001437	0.080361
Potassium	KCNA5	3402039	4.53105	4.73508	5.25132	4.60999	5.12644	4.74853	4.86233	4.65645	4.51413	5.09219	4.48786	4.88729	4.89195	4.77545	4.54937	4.71002	4.79949	5.25864	5.09308	4.99537	0.032101	0.788731	0.906503
Potassium	KCNJ4	3960605	6.34042	6.07768	5.47025	5.85372	6.10288	4.72294	5.44935	5.75547	5.04853	5.67039	6.40401	6.085	5.6838	5.81055	6.06094	5.69453	5.85239	5.99497	6.03495	6.67635	0.380586	0.023358	0.20044
Potassium	KCNA4	3367580	4.07692	4.8185	4.96259	4.97561	4.46972	4.08661	4.27735	5.21973	4.24957	4.7847	5.70467	5.03771	5.53606	5.72441	5.34863	4.96118	4.9182	5.48468	4.8455	5.41836	0.70581	0.000295	0.052205
Potassium	KCNA3	2427619	5.41066	6.35233	8.40448	6.51498	7.30303	7.03199	7.25972	6.18337	7.30503	6.48871	6.64946	6.56955	7.80695	6.39401	7.0904	7.41532	7.27975	7.37409	5.74487	6.56931	0.063941	0.809402	0.915341
Potassium	HCN1	2855963	10.4408	9.34681	10.5036	11.0155	10.2846	10.9845	11.0231	11.147	11.0951	10.4586	9.98733	10.3427	10.4761	10.4789	10.6607	10.5053	10.5534	9.93731	8.48018	10.2838	-0.45939	0.16462	0.450685
Potassium	HCN2	3815045	5.8553	6.02531	5.39004	6.38358	6.679	6.07162	6.40731	6.71448	6.98489	7.1997	5.88604	6.24562	6.05048	6.2198	6.32317	6.95046	6.63548	6.38755	6.16831	6.62685	-0.02175	0.90025	0.957094
Potassium	HCN3	2360818	7.19556	6.76939	6.55855	6.26345	5.83428	6.39777	5.38168	6.58177	6.71774	6.5309	6.57959	6.72342	7.16443	6.13977	7.21808	6.9683	7.21691	7.02867	6.93839	7.66712	0.541359	0.046911	0.263487
Potassium	HCN4	3632424	6.11822	5.86743	5.76432	6.12182	5.66634	5.22677	5.56308	5.72685	5.89348	6.62743	5.78577	5.54577	5.87423	5.27856	6.08808	5.76446	5.22017	5.6543	4.67938	6.07402	-0.2611	0.160694	0.445494
Sodium	SCN1A	2585322	6.18733	6.25488	6.27489	6.02553	5.98809	7.06024	7.41775	7.04089	7.1269	6.87379	6.62607	6.61135	6.69027	6.48984	6.99184	6.67896	6.59748	6.99316	6.53531	8.01483	0.197882	0.36046	0.642041
Sodium	SCN2A	2513471	10.4258	11.4027	11.7509	11.7868	12.0013	11.9413	11.9629	11.7439	11.5927	10.7427	9.89516	11.0861	11.769	11.1661	11.5723	11.3797	11.6029	11.8779	11.2524	11.4379	-0.23115	0.104881	0.369429
Sodium	SCN3A	2584957	6.11343	6.84199	7.76991	7.49557	7.09507	7.94217	7.98857	7.35174	7.33358	6.60368	7.63316	7.51278	8.2843	6.89309	8.18647	7.73262	7.7722	8.42362	7.30464	7.98469	0.519186	0.055698	0.282453
Sodium	SCN4A	3766549	4.55726	4.74858	4.48518	4.78357	4.66389	5.1416	4.71984	4.80439	4.42543	4.77068	4.30486	4.6303	4.33094	5.12163	4.67435	4.62732	4.44139	4.73745	4.95304	4.87741	-0.04017	0.686574	0.853656
Sodium	SCN5A	2669641	4.49117	5.87622	4.79089	4.91645	4.50205	4.68723	4.37649	4.90749	4.31603	4.9067	5.22917	4.80429	4.62255	5.032	4.55129	4.88299	5.13897	5.23141	5.44849	5.43001	0.260045	0.210178	0.50418
Sodium	SCN8A	3414969	7.82391	7.60184	9.44439	9.13482	9.20652	9.32929	9.41904	9.14548	9.16614	7.97662	8.44901	8.16303	9.13115	8.21342	8.70229	9.45341	9.4096	9.12777	8.79182	9.31905	0.05125	0.809586	0.915432
Sodium	SCN9A	2585400	4.17683	4.75811	4.16816	4.06942	4.02056	4.21085	4.04048	4.28975	3.95755	4.18879	4.35468	4.30224	4.09323	4.15819	4.06269	4.18213	4.3075	4.42345	4.5057	4.4968	0.100611	0.262927	0.556169
Sodium	SCN10A	2669732	3.94407	4.75744	4.22199	3.98749	4.12511	4.0148	3.86966	4.16434	4.06804	4.36741	4.09855	3.91523	3.72459	4.103	4.18437	4.21799	4.10053	4.23746	4.29274	4.513	-0.01329	0.90967	0.961766
Sodium	SCN11A	2669803	3.94453	4.42812	4.30263	3.98506	4.01577	4.16788	3.85281	4.0651	3.85936	4.30126	4.25527	4.02139	3.92742	4.37578	4.04098	4.30951	4.24705	4.27551	4.46015	4.47282	0.146336	0.188522	0.480249
Sodium	SCN1B	3830051	9.95822	9.60907	9.09504	9.42627	9.90435	9.59526	9.60445	9.72084	10.1624	10.0775	8.23507	9.24148	8.85725	9.2538	9.39817	8.94937	9.61184	9.17547	9.07331	9.18642	-0.61712	0.004011	0.104946
Sodium	SCN2B	3393652	7.79876	6.59991	7.37471	8.0079	8.81494	9.15326	9.01634	8.68184	9.31452	9.64719	6.1738	7.50504	8.15182	7.45866	8.6186	8.77526	8.59404	8.93772	8.29742	9.78911	-0.21079	0.411862	0.68089
Sodium	SCN3B	3395691	8.58969	8.29535	8.16496	8.45406	6.43153	6.585	7.73433	7.92237	6.15473	6.2976	9.00091	8.51624	9.11373	6.87825	7.43052	7.34609	7.16863	8.15435	5.18672	5.33779	-0.04964	0.866268	0.942438
Sodium	SCN4B	3393622	7.45468	6.92573	6.68475	7.8805	7.80714	7.66308	8.09904	8.10452	8.69453	8.20546	5.86161	6.95466	7.27591	7.26443	7.09739	7.55968	8.18445	7.1975	8.4193	8.36054	-0.3344	0.128568	0.404565
Glutamate	GRM1	2929571	9.4005	8.93756	9.33096	9.87273	9.33907	9.45584	9.45505	9.39024	9.31717	8.75371	8.54255	8.57368	9.4127	8.7766	9.15061	8.91006	8.83241	8.60576	7.6943	8.44508	-0.63091	0.002796	0.095869
Glutamate	GRM2	2623308	7.0303	6.64405	4.77514	5.99169	4.76192	5.94841	5.16727	6.20791	6.57896	6.84066	6.05116	5.55185	5.4625	5.53487	4.88681	5.01223	5.23427	5.21363	5.085	5.23653	-0.66775	0.020211	0.188129
Glutamate	GRM3	3011180	8.77526	8.3347	7.15041	9.08949	7.93624	9.0802	9.38841	9.1612	8.70451	8.71776	8.05065	8.20293	7.06206	8.5521	8.81003	9.03714	7.6739	8.62103	8.50397	8.62977	-0.31946	0.157478	0.441728
Glutamate	GRM4	2950970	8.85547	8.34437	9.80059	9.61621	9.83761	10.4625	9.18205	9.60193	9.80978	9.99181	8.5283	9.53667	9.52133	9.32322	9.72678	9.40532	9.29478	9.36504	9.24442	8.85229	-0.27042	0.219473	0.513798
Glutamate	GRM5	3385834	7.9371	7.15736	7.10817	7.58622	7.17376	7.62756	8.1265	6.82273	6.27834	6.68866	8.50303	7.40929	7.6332	7.37907	8.16145	7.79801	6.92066	7.49412	7.96973	7.09321	0.385537	0.14148	0.419506
Glutamate	GRM6	2889848	4.69617	5.12094	4.20384	4.7607	4.29923	4.44326	4.60675	4.84085	4.7442	4.82147	4.33468	4.734	4.4275	4.60406	4.41108	4.44034	4.31346	4.47293	4.56403	4.68893	-0.15464	0.045462	0.261417
Glutamate	GRM7	2609061	5.87762	5.29747	4.5719	5.49864	4.83301	5.06465	5.24669	5.97306	5.48247	6.92368	5.04877	5.90606	4.49084	6.0485	6.05823	5.94469	5.57719	5.33169	4.94253	5.45997	0.003928	0.98874	0.994404
Glutamate	GRM8	3071063	4.12905	4.86893	4.91509	4.19557	4.06021	4.181	4.90716	4.84036	4.13665	5.31784	5.08134	4.13638	4.85554	4.57169	4.16933	3.99599	4.77746	4.37298	4.30938	4.88226	-0.03995	0.798318	0.91043
Glutamate	GRIK1	3928211	9.48318	7.76307	7.12669	8.3454	8.18392	7.36361	7.60703	8.50561	7.81434	8.24859	9.59039	9.71032	8.43763	9.46746	8.90093	9.51595	8.79462	7.77535	6.69438	8.44762	0.689321	0.073349	0.317501
Glutamate	GRIK2	2918982	10.7422	11.4999	11.2632	11.0696	11.098	11.2724	11.0105	11.1744	10.8702	10.232	10.6209	11.1837	11.5905	11.1116	10.8433	10.2991	10.7809	11.1411	10.5732	10.3623	-0.17258	0.152438	0.434968
Glutamate	GRIK3	2406926	7.51475	6.66044	6.59741	7.49802	7.02636	7.74512	8.24994	7.67963	8.19896	8.13402	6.94572	7.40932	6.33968	7.45126	7.21151	7.51413	8.00695	7.01443	6.4029	7.59512	-0.34136	0.135163	0.412132
Glutamate	GRIK4	3352618	8.16836	7.08804	7.09744	8.03031	7.63305	7.91488	8.27741	8.08175	7.73961	8.06134	7.16022	7.95535	7.17484	7.76985	7.88755	8.32961	7.25483	6.94079	7.62465	7.38907	-0.26054	0.259217	0.552986
Glutamate	GRIK5	3863380	8.42683	8.05099	8.45636	8.35972	8.4496	8.09419	7.94203	7.79887	8.39858	8.06708	8.4193	8.56588	8.90035	8.09547	7.94424	7.78733	8.22033	7.67775	7.83823	7.34225	-0.12531	0.379704	0.656278
Glutamate	GRIA1	2836242	10.7258	10.6949	10.4752	10.9146	10.8447	10.865	11.2872	10.5706	10.0366	10.1124	11.0417	10.4926	10.5107	10.5995	11.2595	11.2522	9.90599	10.8789	11	10.928	0.134209	0.5392	0.765735
Glutamate	GRIA2	2749222	9.92934	10.1394	10.8087	10.2485	10.5659	10.8226	10.3318	9.92246	10.1803	9.64577	10.6849	10.2545	11.3583	10.1017	10.2309	10.2836	10.3531	10.4572	10.4155	10.1036	0.164853	0.246237	0.541346
Glutamate	GRIA3	3989448	7.60344	6.89258	6.9687	8.68555	7.22262	7.42346	8.34409	7.84798	7.77692	7.5379	7.47549	7.1054	7.11401	7.744	7.31734	8.02739	7.44099	6.53873	6.13471	6.87106	-0.45341	0.085438	0.338378
Glutamate	GRIA4	3347118	9.62383	9.32346	9.60623	9.84958	9.38352	9.92919	10.2047	9.78255	9.37871	9.27365	9.99234	9.90186	10.268	9.64479	10.0325	9.96407	9.85651	9.94583	9.5366	9.79295	0.258003	0.048328	0.265899
Glutamate	GRIN1	3195238	7.83587	7.28308	7.79107	7.2722	7.62376	7.99729	7.20801	7.19543	7.96648	7.95484	7.18485	7.58984	7.71079	6.9536	6.845	7.14136	7.4056	6.58934	7.10867	6.86995	-0.4729	0.012023	0.15411
Glutamate	GRIN2A	3679812	7.36373	7.12196	9.36197	9.20891	8.25839	9.00261	8.89762	8.59891	8.39299	6.97396	7.01845	8.0213	8.68074	7.8778	8.66673	8.16746	8.124	8.12714	6.98749	8.22496	-0.3285	0.278768	0.571152
Glutamate	GRIN2B	3445326	7.15861	5.69366	8.15981	6.72227	5.52149	6.43217	7.57654	6.65252	7.24711	6.11883	9.56274	7.49662	7.91067	5.81927	6.32161	5.6113	6.01889	6.17724	4.52846	5.80329	-0.20329	0.68339	0.85216
Glutamate	GRIN2C	3770422	8.4899	7.9469	8.24562	8.66415	8.63937	8.7965	7.96634	8.66512	8.38281	8.45265	6.41348	7.57825	8.3475	7.66116	7.75627	7.78053	8.07061	7.70628	8.13961	7.36402	-0.74317	0.006143	0.121193
Glutamate	GRIN2D	3837759	5.83275	5.44855	5.36826	5.70068	5.28806	5.27184	5.57643	5.46706	5.58157	5.71445	6.13382	6.14335	5.77141	5.41607	5.45691	5.85607	5.2327	5.0873	5.18372	5.40185	0.043355	0.758672	0.891537
Glutamate	GRIN3A	3218151	4.12668	4.72033	3.87035	3.77858	3.69653	3.60486	3.81998	3.98959	3.44673	3.8911	4.79754	4.04153	3.91118	3.77341	3.89346	3.7912	3.59408	3.76732	3.97585	3.95655	0.055739	0.658596	0.837737
Glutamate	GRID1	3298557	6.91956	6.90694	5.746	6.75825	6.57	6.5097	6.73775	6.45169	6.50906	7.1925	6.57643	6.65243	6.82386	6.21405	6.38858	5.95271	6.06659	6.69533	5.93382	6.51643	-0.24812	0.183261	0.47414
Glutamate	GRID2	2736060	11.5224	11.2944	10.6291	11.2454	10.9324	11.2327	11.3397	11.3302	11.2938	11.2014	11.1562	11.2469	11.0173	11.3334	11.3315	11.448	10.9354	11.0201	11.4558	11.0383	-0.00386	0.968486	0.986466
Glutamate	SYP	4007899	10.5499	9.45981	9.57019	10.6273	10.5731	10.3881	9.54535	10.4626	10.6917	11.6725	10.3717	9.84601	10.1372	9.72189	10.1942	10.8408	10.5247	10.1276	10.396	10.1266	-0.12539	0.609807	0.80876
Glutamate	SYN1	4007126	10.131	10.1355	8.7335	10.1572	9.64393	9.74819	9.79106	10.3338	10.508	10.3286	8.63097	9.61449	8.90125	9.32292	9.38671	9.5356	9.58745	9.70629	8.73912	9.17232	-0.69137	0.006549	0.12338
Glutamate	DLG4	3743393	10.2692	9.71334	9.38457	10.8315	9.9982	10.1717	10.2689	11.1726	10.6911	11.0295	9.50624	10.362	9.82187	9.96568	9.84972	9.53961	10.0535	9.70751	8.92606	9.86433	-0.59341	0.040009	0.246509
Glutamate	NLGN1	2652801	9.66134	8.95397	10.5488	10.4268	9.55232	10.1362	10.5493	9.90002	9.82081	8.34852	10.0011	10.0078	10.8184	9.43144	10.1293	8.85915	9.42993	9.90314	8.3817	9.30327	-0.16329	0.602151	0.804136
Glutamate	SLC17A7	3867842	12.034	11.5777	11.1303	12.2157	11.6409	11.3614	11.5476	12.0447	11.803	11.3312	9.71332	11.1208	11.212	10.9152	11.1125	10.8904	11.0031	10.7686	9.53144	10.2316	-1.01875	0.002907	0.097261
Glutamate	SHANK1	3868587	9.17993	8.40669	8.77442	8.9168	9.08431	9.1486	9.10367	8.69062	8.82309	8.60207	8.48808	8.69559	8.78498	8.68967	8.61499	9.29408	8.75907	8.44891	8.4761	8.64436	-0.18344	0.086503	0.340066
Glutamate	SHANK2	3380365	8.68045	7.77537	8.29598	8.3461	7.92538	8.58798	7.91412	7.30505	7.95506	8.38975	7.98398	8.1431	8.52089	8.0068	7.81707	8.33968	8.10958	7.25479	6.86622	7.25593	-0.28772	0.121144	0.394624

Supplementary data 4 - List of differentially expressed in the dorsolateral prefrontal cortex (DFC)

Altered expression of genes with a fold-change (FC) >=1.3 and p<0.01

GENE LIST	p<0.01	|FC|>1.3 & p<0.01
GRIN2A	GRIN2A	GRIN2A
KCNF1	KCNF1	KCNF1
KCNC4	KCNC4	KCNC4
KCNK3	KCNK3	KCNK3
KCNH1	KCNH1	KCNH1
KCNQ3	KCNQ3	KCNQ3
GRIN1	GRIN1	GRIK3
GRIK3	GRIK3	KCNK5
KCNK5	KCNK5	KCNC2
KCNC2	KCNC2	GRIK4
GRIK4	GRIK4	KCND3
KCND3	KCND3	KCNB2
KCNB2	KCNB2	KCNA1
KCNA1	KCNA1	
GRIN2D		
KCNN2		
KCNH3		
SCN4A		
KCNJ5		
KCNQ4		
KCNH6		
KCNG4		
SCN5A		
KCNB1		
KCNK6		
SCN1A		
KCNC3		
KCNA6		
KCNJ18		
KCNK1		
KCNV2		
KCNV1		
KCNJ9		
GRM2		
KCND2		
KCNJ15		
SLC17A7		
KCNJ3		
KCNQ1		
SCN3B		
GRM3		
GRIA4		
KCNK13		
KCNN1		
GRM7		
KCNK2		
GRID1		
GRIN2B		
SCN9A		
KCNA2		
SYN1		
KCNH8		
SCN11A		
GRM8		
KCNG1		
HCN3		
DLG4		
KCNC1		
SHANK2		
SCN2A		
KCNQ5		
KCNS3		
GRIA1		
KCNH7		
KCNJ14		
SCN8A		
KCNK9		
GRIA3		
KCNK12		
KCNK18		
KCNS2		
GRIK1		
GRIA2		
KCNT2		
HCN4		
KCNN3		
SCN4B		
HCN1		
GRIK2		
KCNN4		
GRM6		
SHANK1		
KCNK16		
KCNJ13		
KCNK17		
KCNJ11		
GRM4		
KCNJ2		
KCNJ16		
KCNMA1		
SCN2B		
KCNJ6		
KCNJ10		
NLGN1		
KCNJ8		
KCNS1		
GRIN3A		
SCN3A		
KCNK10		
KCNK4		
KCNG2		
SCN10A		
KCNA10		
SCN1B		
SYP		
GRID2		
GRIK5		
KCND1		
HCN2		
KCNH4		
KCNJ1		
KCNK7		
KCNK15		
KCNH5		
KCNQ2		
GRM1		
KCNG3		
KCNA7		
KCNU1		
KCNH2		
GRIN2C		
KCNA5		
GRM5		
KCNJ4		
KCNA4		
KCNA3		

Supplementary data 5 - Heatmap showing expression fold Change >= 1.3 and p<0.01

Group	Gene	ID_REF	Con_DFC_	Con_DFC_	Con_DFC_	Con_DFC_	Con_DFC_	Con_DFC_	Con_DFC_	Con_DFC_	Con_DFC_	Con_DFC_	Con_DFC_	Con_DFC_	DS_DFC_St	DS_DFC_St	DS_DFC_St	DS_DFC_St	DS_DFC_St	DS_DFC_St	DS_DFC_St	DS_DFC_St	DS_DFC_St	DS_DFC_St	DS_DFC_St	DS_DFC_St	Log2FC	pvalue	FDR
Glutamate	GRIN2A	3679812	6.10714	6.77851	10.3608	9.36816	9.13758	9.69644	10.5161	9.75684	9.42188	9.723	8.63459	9.76603	4.92043	5.93043	8.92859	9.70293	8.46645	9.14424	8.69894	9.20708	8.11799	8.37273	8.64369	8.54121	-0.8827	0.000489	0.049618
Potassium	KCNF1	2469627	6.39239	6.5778	7.13753	6.75006	7.49189	6.45035	7.58204	7.16916	6.74213	7.20943	7.28432	6.63193	5.61782	5.53562	6.02626	6.98758	5.93758	6.55342	7.24814	6.98819	5.58086	6.06083	6.36482	5.16142	-0.77971	0.000873	0.05716
Potassium	KCNC4	2351294	6.27012	6.44236	7.55261	6.80305	6.93213	7.18034	7.3342	6.64058	7.77345	6.86981	7.18699	7.0007	5.71625	5.95901	6.96811	6.67611	6.14736	6.76083	6.51775	6.68477	5.53909	6.43926	6.40295	6.45597	-0.64324	0.002234	0.077798
Potassium	KCNK3	2473936	5.89775	6.32896	7.69524	6.46529	6.99961	6.44722	7.25075	6.91869	6.74702	6.94704	6.45617	6.55479	6.03326	5.59506	6.01718	6.64304	6.93064	6.23675	6.30418	6.56561	5.76706	5.88345	5.77188	5.69554	-0.60541	0.003012	0.084342
Potassium	KCNH1	2454140	5.16101	4.84467	7.96398	8.33697	8.67737	9.19243	9.78577	9.63783	9.65335	9.59559	9.51188	9.79924	4.59501	5.0093	6.66936	8.96639	7.74935	8.75632	9.17215	9.17752	8.31263	8.37919	9.00603	8.7575	-0.63411	0.003431	0.088655
Potassium	KCNQ3	3154002	9.53081	10.035	10.7814	9.6019	10.3239	9.93733	10.0966	10.2831	9.71771	9.31478	9.1142	9.56213	9.48693	9.18744	10.2387	9.70064	10.0051	9.86856	8.54037	9.57086	8.54479	8.8483	8.73257	9.36948	-0.51709	0.003722	0.090748
Glutamate	GRIK3	2406926	10.1052	9.15836	9.97605	8.40295	7.96809	8.49264	8.67691	8.67644	8.65685	9.00167	8.81143	8.08814	10.1411	9.42466	8.97416	8.15813	8.19844	8.14684	7.22883	7.67089	6.76241	6.76452	7.45928	6.50194	-0.88196	0.004334	0.096319
Potassium	KCNK5	2952834	4.47159	5.45324	4.95615	5.55506	5.16784	4.93428	4.76816	5.18213	4.81433	5.14154	5.2192	4.9394	5.13816	5.64674	4.94418	5.26268	5.64688	5.75236	5.79016	5.14018	5.94944	5.61094	5.4494	5.62798	0.446348	0.005048	0.102608
Potassium	KCNC2	3462567	8.57515	8.03813	10.1676	9.30907	11.0614	8.83374	11.0401	11.0446	10.408	10.1779	9.76954	10.6529	6.53161	6.77728	8.93292	9.75532	9.84525	9.11858	10.0797	10.4501	8.2694	9.19981	10.2208	9.4255	-0.87266	0.005393	0.104738
Glutamate	GRIK4	3352618	7.27962	7.12795	8.43943	7.80409	8.16993	8.44398	8.82329	7.98322	8.23127	7.97187	8.18898	8.36823	6.13846	6.35946	7.83931	7.56769	8.27987	8.23591	7.82709	7.78749	6.65364	8.3401	7.58998	7.76225	-0.53755	0.005813	0.107475
Potassium	KCND3	2428119	7.33767	7.11952	8.07453	6.63761	7.14137	6.70106	8.16815	8.52788	8.56639	8.75992	7.28641	8.52539	5.97753	5.45133	6.91	6.87384	6.66378	6.99447	6.93166	7.5868	6.32823	6.76769	8.03281	7.44016	-0.9073	0.006331	0.110913
Potassium	KCNB2	3103062	7.29499	8.45801	8.03907	7.1275	6.91407	6.95024	8.25618	8.15796	7.25266	8.13209	7.16823	8.41306	7.72697	7.89809	8.0219	7.39271	6.00691	6.91937	6.7248	6.95143	6.19913	6.79339	6.70716	6.55303	-0.6891	0.008261	0.120192
Potassium	KCNA1	3401994	4.91811	5.32056	9.10055	6.34283	8.58671	7.22598	10.1692	9.75657	9.5546	10.2357	9.77793	9.63716	5.05551	4.61726	6.09208	8.19831	6.95175	7.82181	7.63647	7.62237	6.9652	7.14306	7.6498	7.40626	-1.4555	0.008499	0.120808

Supplementary data 6 - Heatmap showing reduction of gene expression (fold change >= 1.3 and p<0.01) in the dorsolateral prefrontal cortex (DFC) and cerebellar cortex (CBC)

Group	Gene	ID_REF	Con_DFC_S	Con_DFC_S	Con_DFC_S	Con_DFC_S	Con_DFC_S	Con_DFC_S	Con_DFC_S	Con_DFC_S	Con_DFC_S	Con_DFC_S	Con_DFC_S	Con_DFC_S	DS_DFC_St	DS_DFC_St	DS_DFC_St	DS_DFC_St	DS_DFC_St	DS_DFC_St	DS_DFC_St	DS_DFC_St	DS_DFC_St	DS_DFC_St	DS_DFC_St	DS_DFC_St	Log2FC	pvalue	FDR
Glutamate	GRIN2A	3679812	6.10714	6.77851	10.3608	9.36816	9.13758	9.69644	10.5161	9.75684	9.42188	9.723	8.63459	9.76603	4.92043	5.93043	8.92859	9.70293	8.46645	9.14424	8.69894	9.20708	8.11799	8.37273	8.64369	8.54121	-0.8827	0.000489	0.049618
Potassium	KCNF1	2469627	6.39239	6.5778	7.13753	6.75006	7.49189	6.45035	7.58204	7.16916	6.74213	7.20943	7.28432	6.63193	5.61782	5.53562	6.02626	6.98758	5.93758	6.55342	7.24814	6.98819	5.58086	6.06083	6.36482	5.16142	-0.77971	0.000873	0.05716
Potassium	KCNC4	2351294	6.27012	6.44236	7.55261	6.80305	6.93213	7.18034	7.3342	6.64058	7.77345	6.86981	7.18699	7.0007	5.71625	5.95901	6.96811	6.67611	6.14736	6.76083	6.51775	6.68477	5.53909	6.43926	6.40295	6.45597	-0.64324	0.002234	0.077798
Potassium	KCNK3	2473936	5.89775	6.32896	7.69524	6.46529	6.99961	6.44722	7.25075	6.91869	6.74702	6.94704	6.45617	6.55479	6.03326	5.59506	6.01718	6.64304	6.93064	6.23675	6.30418	6.56561	5.76706	5.88345	5.77188	5.69554	-0.60541	0.003012	0.084342
Potassium	KCNH1	2454140	5.16101	4.84467	7.96398	8.33697	8.67737	9.19243	9.78577	9.63783	9.65335	9.59559	9.51188	9.79924	4.59501	5.0093	6.66936	8.96639	7.74935	8.75632	9.17215	9.17752	8.31263	8.37919	9.00603	8.7575	-0.63411	0.003431	0.088655
Potassium	KCNQ3	3154002	9.53081	10.035	10.7814	9.6019	10.3239	9.93733	10.0966	10.2831	9.71771	9.31478	9.1142	9.56213	9.48693	9.18744	10.2387	9.70064	10.0051	9.86856	8.54037	9.57086	8.54479	8.8483	8.73257	9.36948	-0.51709	0.003722	0.090748
Glutamate	GRIK3	2406926	10.1052	9.15836	9.97605	8.40295	7.96809	8.49264	8.67691	8.67644	8.65685	9.00167	8.81143	8.08814	10.1411	9.42466	8.97416	8.15813	8.19844	8.14684	7.22883	7.67089	6.76241	6.76452	7.45928	6.50194	-0.88196	0.004334	0.096319
Potassium	KCNC2	3462567	8.57515	8.03813	10.1676	9.30907	11.0614	8.83374	11.0401	11.0446	10.408	10.1779	9.76954	10.6529	6.53161	6.77728	8.93292	9.75532	9.84525	9.11858	10.0797	10.4501	8.2694	9.19981	10.2208	9.4255	-0.87266	0.005393	0.104738
Glutamate	GRIK4	3352618	7.27962	7.12795	8.43943	7.80409	8.16993	8.44398	8.82329	7.98322	8.23127	7.97187	8.18898	8.36823	6.13846	6.35946	7.83931	7.56769	8.27987	8.23591	7.82709	7.78749	6.65364	8.3401	7.58998	7.76225	-0.53755	0.005813	0.107475
Potassium	KCND3	2428119	7.33767	7.11952	8.07453	6.63761	7.14137	6.70106	8.16815	8.52788	8.56639	8.75992	7.28641	8.52539	5.97753	5.45133	6.91	6.87384	6.66378	6.99447	6.93166	7.5868	6.32823	6.76769	8.03281	7.44016	-0.9073	0.006331	0.110913
Potassium	KCNB2	3103062	7.29499	8.45801	8.03907	7.1275	6.91407	6.95024	8.25618	8.15796	7.25266	8.13209	7.16823	8.41306	7.72697	7.89809	8.0219	7.39271	6.00691	6.91937	6.7248	6.95143	6.19913	6.79339	6.70716	6.55303	-0.6891	0.008261	0.120192
Potassium	KCNA1	3401994	4.91811	5.32056	9.10055	6.34283	8.58671	7.22598	10.1692	9.75657	9.5546	10.2357	9.77793	9.63716	5.05551	4.61726	6.09208	8.19831	6.95175	7.82181	7.63647	7.62237	6.9652	7.14306	7.6498	7.40626	-1.4555	0.008499	0.120808
