# Supplementary material for: Synaptic and intrinsic membrane defects disrupt early neural network dynamics in Down syndrome
Source: Nat Commun. 2026 Jan 22;17:1287. doi: 10.1038/s41467-025-68048-x (PMC12868644; doi:10.1038/s41467-025-68048-x)
Supplement: Supplementary file 2 — Description of Additional Supplementary Files [file 41467_2025_68048_MOESM2_ESM.pdf]

Description of Additional Supplementary Files

File name: Supplementary data 1

Description: Statistical analysis of results.

File name: Supplementary data 2

Description: Expression of K^+ channel, HCN channel, Na^+ channel and glutamatergic synapse genes in human dorsolateral prefrontal cortex across development and adulthood.

File name: Supplementary data 3

Description: Expression of K^+ channel, HCN channel, Na^+ channel and glutamatergic synapse genes in human cerebellar cortex across development and adulthood.

File name: Supplementary data 4

Description: List of differentially expressed genes in the dorsolateral prefrontal cortex (DFC) and cerebellar cortex (CBC).

File name: Supplementary data 5

Description: Heatmap showing gene expression-fold change >= 1.3 and $p<0.01$ in the dorsolateral prefrontal cortex (DFC) and cerebellar cortex (CBC).

File name: Supplementary data 6

Description: Heatmap showing reduction of gene expression (fold change >= 1.3 and $p<0.01$) in the dorsolateral prefrontal cortex (DFC) and cerebellar cortex (CBC).
