# Supplementary material for: Synaptic and intrinsic membrane defects disrupt early neural network dynamics in Down syndrome
Source: Nat Commun. 2026 Jan 22;17:1287. doi: 10.1038/s41467-025-68048-x (PMC12868644; doi:10.1038/s41467-025-68048-x)
Supplement: Supplementary file 3 — Supplementary Data 1 [file 41467_2025_68048_MOESM3_ESM.pdf]

Supplementary data 1 – Statistical analysis of results

Basal microelectrode array						
Parameter	Week	Disomic	Trisomic	Test	P-Value	Figure
Active electrodes	4	42.1 ± 5.7 (8 wells)	13.1 ± 7.9 (7 wells)	Mann-Whitney test	0.0140	Figure S3B
	5	48.9 ± 3.6 (11)	18.4 ± 5.6 (9)	Mann-Whitney test	0.0027	
	6	44.18 ± 5.1 (11)	15.4 ± 2.4 (9)	Unpaired t-test	0.0002	
	7	43.9 ± 4.4 (11)	16.9 ± 2.2 (9)	Unpaired t-test	0.0001	
	8	40.6 ± 5.6 (11)	17.5 ± 2.1 (8)	Unpaired t-test	0.0037	
	9	38.7 ± 6.6 (11)	21.2 ± 3 (8)	Unpaired t-test	0.0485	
	10	41.6 ± 7.9 (8)	25.6 ± 3 (7)	Unpaired t-test	0.0948	
	11	47.2 ± 9.6 (5)	26 ± 2.7 (5)	Unpaired t-test	0.0674	
	12	62.8 ± 1 (4)	23.6 ± 2.4 (5)	Unpaired t-test	2.66E-06	
	14	54.5 ± 6.9 (4)	32.5 ± 1.4 (4)	Unpaired t-test	0.0201	
Number of spikes	4	39733 ± 8955 (8 wells)	6304 ± 5305 (7 wells)	Mann-Whitney test	0.0037	Figure S3C
	5	65110 ± 8915 (11)	10050 ± 3879 (9)	Mann-Whitney test	0.000083	
	6	63776 ±12412 (11)	7614 ± 2039 (9)	Unpaired t-test	0.0008	
	7	75609 ± 13041 (11)	9656 ± 1930 (9)	Unpaired t-test	0.0003	
	8	88607 ± 19327 (11)	11293 ± 2329 (8)	Unpaired t-test	0.0036	
	9	101146 ± 24497 (11)	14989 ± 3476 (8)	Unpaired t-test	0.0088	
	10	126481 ± 35296 (8)	18978 ± 3250 (7)	Unpaired t-test	0.0143	
	11	188302 ± 67428 (5)	13930 ± 3355 (5)	Unpaired t-test	0.0325	
	12	354403 ± 44013 (4)	19604 ± 6125 (5)	Unpaired t-test	0.000061	
	14	338730 ±107206 (4)	38555 ± 8773 (4)	Unpaired t-test	0.0316	
Mean firing rate (Hz)	4	1.01 ± 0.2 (8 wells)	0.16 ± 0.13121 (7 wells)	Mann-Whitney test	0.0022	Figure S3D
	5	1.69 ± 0.2 (11)	0.26 ± 0.1 (9)	Mann-Whitney test	0.000083	
	6	1.64 ± 0.3 (11)	0.2 ± 0.05 (9)	Unpaired t-test	0.0007	
	7	1.95 ± 0.3 (11)	0.25 ± 0.1 (9)	Unpaired t-test	0.0003	
	8	2.29 ± 0.5 (11)	0.29 ± 0.1 (8)	Unpaired t-test	0.0036	
	9	2.53 ± 0.6 (11)	0.36 ± 0.1 (8)	Unpaired t-test	0.0081	
	10	3.28 ± 0.9 (8)	0.49 ± 0.1 (7)	Unpaired t-test	0.0145	
	11	4.89 ± 1.7 (5)	0.36 ± 0.1 (5)	Unpaired t-test	0.0325	
	12	9.21 ± 1.1 (4)	0.51 ± 0.16 (5)	Unpaired t-test	0.00006	
	14	8.8 ± 2.8 (4)	1 ± 0.2 (4)	Unpaired t-test	0.0319	
Weighted Mean firing rate (Hz)	4	1.35 ± 0.19 (8 wells)	0.38 ± 0.11 (7 wells)	Unpaired t-test	0.0010	Figure 1C
	5	2.1 ± 0.21 (11)	0.77 ± 0.19 (9)	Unpaired t-test	0.0003	
	6	2.25 ± 0.29 (11)	0.78 ± 0.15 (9)	Unpaired t-test	0.0005	

	7	2.66 ± 0.27 (11)	1.06 ± 0.28 (9)	Unpaired t-test	0.0008	
	8	3.43 ± 0.41 (11)	1.03 ± 0.14 (8)	Unpaired t-test	0.0002	
	9	3.57 ± 0.49 (11)	1.1 ± 0.22 (8)	Unpaired t-test	0.0008	
	10	4.36 ± 0.76 (8)	1.22 ± 0.19 (7)	Unpaired t-test	0.0023	
	11	5.9 ± 1.39 (5)	0.84 ± 0.15 (5)	Unpaired t-test	0.0069	
	12	9.42 ± 1.07 (4)	1.49 ± 0.35 (5)	Unpaired t-test	0.0004	
	14	8.38 ± 2.58 (4)	1.62 ± 0.44 (4)	Unpaired t-test	0.0420	
Electrode bursts	4	1338.37 ± 290.64 (8)	249.28 ± 216.17 (7)	Mann-Whitney test	0.0059	Figure S3E
	5	1843.36 ± 186.06 (11)	468.89 ± 187.29 (9)	Mann-Whitney test	0.00048	
	6	1509.54 ± 260.77 (11)	294.33 ± 89.48 (9)	Unpaired t-test	0.0008	
	7	1883.27 ± 317.45 (11)	292.78 ± 59.7 (9)	Unpaired t-test	0.0003	
	8	1875.36 ± 316.64 (11)	351.87 ± 58.22 (8)	Unpaired t-test	0.0009	
	9	1771.36 ± 369.23 (11)	577.25 ± 129.18 (8)	Unpaired t-test	0.0167	
	10	2254 ± 630.67 (8)	655.14 ± 119.27 (7)	Unpaired t-test	0.0366	
	11	3480.4 ± 1281.92 (5)	437.6 ± 108.26 (5)	Unpaired t-test	0.0456	
	12	6758.75 ± 1001.06 (4)	581.6 ± 146.42 (5)	Unpaired t-test	0.0002	
	14	6235 ± 1780.23 (4)	1045.5 ± 182.7 (4)	Unpaired t-test	0.0273	
Spikes per electrode burst	4	19.03 ± 3.3 (8 wells)	12.17 ± 3.1 (7 wells)	Mann-Whitney test	0.0813	Figure 1D
	5	27.94 ± 5 (11)	10 ± 0.92 (9)	Unpaired t-test	0.0050	
	6	33.6 ± 5.14 (11)	10.44 ± 0.96 (9)	Unpaired t-test	0.0008	
	7	34.91 ± 4.36 (11)	15.58 ± 5.03 (9)	Mann-Whitney test	0.0097	
	8	39.96 ± 6.39 (11)	10.94 ± 0.6 (8)	Unpaired t-test	0.0013	
	9	45.15 ± 6.62 (11)	9.2 ± 0.56 (8)	Unpaired t-test	0.0003	
	10	47.25 ± 9.92 (8)	11.22 ± 0.73 (7)	Unpaired t-test	0.0050	
	11	56.12 ± 9.44 (5)	9.97 ± 1.26 (5)	Unpaired t-test	0.0013	
	12	54.43 ± 10.91 (4)	14.14 ± 2.69 (5)	Unpaired t-test	0.0052	
	14	44.61 ± 15.34 (4)	16.46 ± 1.59 (4)	Unpaired t-test	0.1177	
Burst Duration (s)	4	0.38 ± 0.04 (8 wells)	0.38 ± 0.12 (7 wells)	Mann-Whitney test	0.3450	Figure 1D
	5	0.47 ± 0.04 (11)	0.37 ± 0.04 (9)	Unpaired t-test	0.0832	
	6	0.50 ± 0.04 (11)	0.38 ± 0.04 (9)	Unpaired t-test	0.0593	
	7	0.51 ± 0.04 (11)	0.42 ± 0.04 (9)	Unpaired t-test	0.0971	
	8	0.54 ± 0.05 (11)	0.36 ± 0.02 (8)	Unpaired t-test	0.0105	
	9	0.59 ± 0.05 (11)	0.33 ± 0.02 (8)	Unpaired t-test	0.0006	
	10	0.58 ± 0.06 (8)	0.38 ± 0.03 (7)	Unpaired t-test	0.0151	
	11	0.62 ± 0.05 (5)	0.33 ± 0.03 (5)	Unpaired t-test	0.0007	
	12	0.61 ± 0.06 (4)	0.38 ± 0.03 (5)	Unpaired t-test	0.0086	
	14	0.54 ± 0.09 (4)	0.4 ± 0.04 (4)	Unpaired t-test	0.2013	

Inter-burst interval	4	48.32 ± 7.12 (8 wells)	61.07 ± 11.54 (7 wells)	Unpaired t-test	0.3420	Figure 1D
	5	34.92 ± 4.93 (11)	51.96 ± 4.39 (9)	Mann-Whitney test	0.0057	
	6	38.04 ± 5.34 (11)	46.42 ± 7.67 (9)	Unpaired t-test	0.3689	
	7	29.65 ± 2.36 (11)	44.65 ± 4.96 (9)	Unpaired t-test	0.0095	
	8	29.36 ± 4.83 (11)	49.84 ± 8.94 (8)	Unpaired t-test	0.0448	
	9	33.09 ± 4.08 (11)	43.58 ± 7.39 (8)	Mann-Whitney test	0.3100	
	10	25.68 ± 4.05 (8)	43.67 ± 7.05 (7)	Unpaired t-test	0.0396	
	11	23.28 ± 5.64 (5)	59.38 ± 9.47 (5)	Unpaired t-test	0.0113	
	12	11.55 ± 1.1 (4)	38.61 ± 10.36 (5)	Unpaired t-test	0.0552	
	14	13.83 ± 5.63 (4)	45.28 ± 2.91 (4)	Unpaired t-test	0.0025	
Number of network bursts	4	23 ± 5.58 (8 wells)	7.71 ± 7.71 (7 wells)	Mann-Whitney test	0.0545	Figure S3F
	5	24.82 ± 2.73 (11)	10.44 ± 4.99 (9)	Unpaired t-test	0.0162	
	6	34.18 ± 8.47 (11)	9.78 ± 4.102 (9)	Unpaired t-test	0.0266	
	7	30.54 ± 4.49 (11)	11.89 ± 2.23 (9)	Mann-Whitney	0.0028	
	8	39.54 ± 5.03 (11)	12.37 ± 2.46 (8)	Unpaired t-test	0.0005	
	9	36.09 ± 3.61 (11)	21.12 ± 2.49 (8)	Mann-Whitney test	0.0105	
	10	46.12 ± 6.64 (8)	27 ± 3.46 (7)	Unpaired t-test	0.0296	
	11	39.8 ± 6.25 (5)	24.4 ± 1.86 (5)	Unpaired t-test	0.0458	
	12	43.75 ± 5.86 (4)	25.6 ± 2.09 (5)	Unpaired t-test	0.0150	
	14	52.5 ± 1.55 (4)	33.25 ± 1.11 (4)	Unpaired t-test	<0.0001	
Network burst frequency	4	0.043 ± 0.01 (7 wells)	0 (5 wells)	Mann-Whitney test	0.0006	Figure 1E
	5	0.041 ± 0.004 (11)	0.031 ± 0.012 (5)	Unpaired t-test	0.3430	
	6	0.062 ± 0.01 (10)	0.029 ± 0.01 (5)	Unpaired t-test	0.1423	
	7	0.05 ± 0.01 (11)	0.02 ± 0.004 (9)	Mann-Whitney test	0.0021	
	8	0.07 ± 0.01 (11)	0.02 ± 0.004 (8)	Unpaired t-test	0.0005	
	9	0.057 ± 0.01 (11)	0.034 ± 0.004 (8)	Unpaired t-test	0.0090	
	10	0.076 ± 0.01 (8)	0.045 ± 0.01 (7)	Mann-Whitney test	0.0078	
	11	0.066 ± 0.01 (5)	0.041 ± 0.0031 (5)	Unpaired t-test	0.0448	
	12	0.073 ± 0.01 (4)	0.043 ± 0.0035 (5)	Unpaired t-test	0.0137	
	14	0.087 ± 0.0026 (4)	0.055 ± 0.0018 (4)	Unpaired t-test	5.49E-05	
Spikes per network burst	4	1397 ± 226 (7 wells)	0 (7 wells)	Unpaired t-test	0.0004	Figure 1E
	5	2001 ± 267 (11)	183 ± 75 (5)	Unpaired t-test	0.0005	
	6	1882 ± 514 (10)	229 ± 49 (5)	Mann-Whitney test	0.0007	
	7	2277 ± 453 (11)	314 ± 80 (9)	Unpaired t-test	0.0011	
	8	2099 ± 555 (11)	292 ± 77 (8)	Mann-Whitney test	0.0002	
	9	2372 ± 571 (11)	380 ± 91 (8)	Unpaired t-test	0.0093	
	10	2451 ± 688 (8)	516 ± 101 (7)	Unpaired t-test	0.0221	
	11	4819 ± 2074 (5)	402 ± 110 (5)	Unpaired t-test	0.0661	

	12	8372 ± 1285 (4)	563 ± 191 (5)	Unpaired t-test	0.0003	
	14	6163 ± 2032 (4)	957 ± 237 (4)	Unpaired t-test	0.0438	
Synchrony (area under normalised cross-correlation curve)	4	0.047 ± 0.022 (7 wells)	0.001 ± 6.67E-4 (7 wells)	Mann-Whitney test	0.0073	Figure 1F
	5	0.07493 ± 0.025 (11)	0.00149 ± 4.88e-4 (5)	Mann-Whitney test	0.0005	
	6	0.09433 ± 0.023 (10)	0.00195 ± 4.03e-4 (5)	Unpaired t-test	0.0009	
	7	0.10019 ± 0.022 (11)	0.00513 ± 0.002 (9)	Mann-Whitney test	0.0006	
	8	0.10471 ± 0.029 (11)	0.00298 ± 6.36e-4 (8)	Mann-Whitney test	0.0005	
	9	0.12173 ± 0.03 (11)	0.0039 ± 6.71e-4 (8)	Unpaired t-test	0.0044	
	10	0.14083 ± 0.037 (8)	0.00727 ± 9.94e-4 (7)	Unpaired t-test	0.0080	
	11	0.16045 ± 0.034 (5)	0.00648 ± 0.001 (5)	Unpaired t-test	0.0018	
	12	0.19306 ± 0.04 (4)	0.00742 ± 0.001 (5)	Unpaired t-test	0.0011	
	14	0.16143 ± 0.057 (4)	0.01079 ± 0.001 (4)	Unpaired t-test	0.0363	
**Microelectrode array - pharmacology in CNQX and APV**						
Parameter	Control	CNQX+APV	Wash	Test	P-value	Figure
Normalised mean firing rate (Hz)	100 (6 wells)	74.1 ± 9.4 (6 wells)	94.5 ± 2.6 (6 wells)	One way-ANOVA	0.0132	Figure S3G-J
Normalised number of bursts	100 (6 wells)	80.4 ± 4.2 (6 wells)	95.2 ± 2.7 (6 wells)	One way-ANOVA	0.0006	
Normalised no. of network bursts	100 (6 wells)	18.3 ± 15.5 (6 wells)	115.3 ± 8.4 (6 wells)	One way-ANOVA	P<0.0001	
Normalised synchrony index	100 (6 wells)	44.8 ± 7.7 (6 wells)	102.5 ± 3.3 (6 wells)	One way-ANOVA	P<0.0001	
**Microelectrode array - pharmacology in tetrodotoxin**						
Parameter	Control	Tetrodotoxin	Wash	Test	P-value	Figure
Normalised mean firing rate (Hz)	100 (8 wells)	0.003 ± 0.002 (8 wells)	69.7 ± 3.1 (8 wells)	One way-ANOVA	P<0.0001	Figure S3G-J
Normalised number of bursts	100 (8 wells)	0 ± 0 (8 wells)	78.3 ± 4.1 (8 wells)	One way-ANOVA	P<0.0001	
Normalised no. of network bursts	100 (8 wells)	0 ± 0 (8 wells)	71.4 ± 13.7 (8 wells)	One way-ANOVA	P<0.0001	
Normalised synchrony index	100 (8 wells)	0 ± 0 (8 wells)	78.9 ± 5.2 (8 wells)	One way-ANOVA	P<0.0001	
**Microelectrode array – clonal parameters for disomic cells**						
Parameter	Genotype	C3 clone	C9 clone	Test	P-value	Figure
Active electrodes	Disomic	56 (13 wells)	53 (6 wells)	Mann-Whitney test	0.6928	Figure S2A
Weighted mean firing rate (Hz)	Disomic	3.9 ± 0.6 (13 wells)	3.9 ± 0.5 (6 wells)	Unpaired t-test	0.967	
Number of electrode bursts	Disomic	2100.2 ± 456 (13 wells)	1702.5 ± 418.7 (6 wells)	Unpaired t-test	0.5955	
Number of network bursts	Disomic	37 (13 wells)	34 (6 wells)	Mann-Whitney test	0.9301	
Synchrony	Disomic	0.06592 (13 wells)	0.1353 (6 wells)	Mann-Whitney test	0.467	
**Microelectrode array – clonal parameters for trisomic cells**						
Parameter	Genotype	C5 clone	C13 clone	Test	P-value	Figure
Active electrodes	Trisomic	22 ± 2.3 (9 wells)	25.2 ± 4.4 (6 wells)	Unpaired t-test	0.492	Figure S2A
Weighted mean firing rate (Hz)	Trisomic	1.14 ± 0.2 (9 wells)	1.17 ± 0.1 (6 wells)	Unpaired t-test	0.9347	
Number of electrode bursts	Trisomic	551 ± 118.1 (9 wells)	707.5 ± 124.1 (6 wells)	Unpaired t-test	0.3927	
Number of network bursts	Trisomic	22.1 ± 3.3 (9 wells)	26.5 ± 2.1 (6 wells)	Unpaired t-test	0.3373	
Synchrony	Trisomic	0.005 ± 0.001 (9 wells)	0.006 ± 0.001 (6 wells)	Unpaired t-test	0.3088	

Excitatory postsynaptic currents (EPSC)						
Parameter	Condition	Disomic	Trisomic	Test	P-value	Figure
EPSC Frequency (Hz)	In Mg^{2+}	2.020 (39 cells)	0.56 (32 cells)	Mann Whitney test	0.00006528	Figure 2C
	0 Mg^{2+}	7.242 (16 cells)	0.7312 (19 cells)	Mann Whitney test	0.00002789	Figure 2H
EPSC Amplitude (pA)	In Mg^{2+}	-36.6 ± 2.2 (39 cells)	-37.7 ± 2.8 (30)	Unpaired t-test	0.762	Figure 2D
	0 Mg^{2+}	-37.25 ± 2.9 (14 cells)	-32.2 ± 1.2 (16 cells)	Unpaired t-test	0.1011	Figure 2I
EPSC kinetics (charge transfer; pA.ms)	In Mg^{2+}	-73.8 ± 7 (27 cells)	-78.8 ± 6.4 (25 cells)	Unpaired t-test	0.6069	Figure S4G
	0 Mg^{2+}	-54.885 (9 cells)	-71.574 (10 cells)	Mann Whitney test	0.3562	Figure S4I
EPSC kinetics (rate of rise; pAms^{-1})	In Mg^{2+}	393.24 (19 cells)	369.77 (25 cells)	Mann Whitney test	0.8311	Figure S4G
	0 Mg^{2+}	350.02 (9 cells)	319.08 (10 cells)	Mann Whitney test	0.447	Figure S4I
EPSC kinetics (decay τ; ms)	In Mg^{2+}	2.277 ± 0.23 (18 cells)	2.153 ± 0.14 (24 cells)	Unpaired t-test	0.6269	Figure S4G
	0 Mg^{2+}	2.187 ± 0.32 (8 cells)	2.447 ± 0.29 (10 cells)	Unpaired t-test	0.5598	Figure S4I
**Excitatory postsynaptic currents (EPSC) – clonal parameters for disomic cells**						
Parameter	Genotype	C3 clone	C9 clone	Test	P-value	Figure
EPSC Frequency (Hz)	Disomic	9.2 ± 2 (9 cells)	7.7 ± 2.5 (7 cells)	Unpaired t-test	0.6488	Figure S2E
**Excitatory postsynaptic currents (EPSC)– clonal parameters for trisomic cells**						
Parameter	Genotype	C5 clone	C13 clone	Test	P-value	Figure
EPSC Frequency (Hz)	Trisomic	0.9839 (12 cells)	0.6024 (7 cells)	Mann Whitney test	0.8369	Figure S2E
**Bursts in 0 Mg^{2+}**						
Parameter		Disomic	Trisomic	Test	P-value	Figure
Bursts rate (min^{-1})		1.319 (20 cells)	0.8745 (28 cells)	Mann Whitney test	0.0395	Figure 3C
Burst amplitude (pA)		117.07 (20 cells)	42.7 (19 cells)	Mann Whitney test	0.0002	Figure 3D
Burst charge transfer (pA.ms)		-722392 ± 161212 (18)	-226700 ± 33474 (17)	Unpaired t-test	0.0061	Figure 3E
Capacitance in Cs-based internal (pF)		89.524 (51 cells)	69.762 (56 cells)	Mann Whitney test	0.0771	Figure S4E
**Action potentials**						
Parameter		Disomic	Trisomic	Test	P-value	Figure
Resting membrane potential (mV)		-50 (112 cells)	-44 (100 cells)	Mann Whitney test	0.061	Figure 4A
Rheobase (pA)		24.5 (72 cells)	16 (62 cells)	Mann Whitney test	0.0939	Figure 4B
Spike threshold (mV)		-34 (85 cells)	-35 (87 cells)	Mann Whitney test	0.1653	Figure 4E
I at max spiking (pA)		72.5 (78 cells)	55 (83 cells)	Mann Whitney test	0.002	Figure 4F
Spike peak (mV)		78.7 ± 2.4 (70 cells)	66.3 ± 3.5 (60 cells)	Unpaired t-test	0.0032	Figure 4J
Spike area (mV.ms)		169.4 (70 cells)	192.80 (60 cells)	Mann Whitney test	0.000033	Figure 4K
Rise time (ms)		1.437 (70 cells)	2 (60 cells)	Mann Whitney test	0.0052	Figure 4L
T_{50} (ms)		1.087 (70 cells)	1.508 (60 cells)	Mann Whitney test	0.000038	Figure 4M
Capacitance using a K-based internal (pF)		72.803 (113 cells)	58.216 (114 cells)	Mann Whitney test	0.0532	Figure S6D
Input resistance using a K-based internal (MΩ)		544.10 (113 cells)	511.73 (114 cells)	Mann Whitney test	0.9557	Figure S6D

Supplementary data 1 – Statistical analysis of results

Membrane time constant (ms)		0.6218 (113 cells)	0.5511 (114 cells)	Mann Whitney test	0.215	Figure S6D
Average number of spikes	Rheo x 1	1 (72 cells)	1 (62 cells)	-	-	Figure 4D
	Rheo x 2	3.583 ± 0.15 (72 cells)	3.095 ± 0.17 (62 cells)	Unpaired t-test	0.0351	
	Rheo x 3	5.035 ± 0.21 (72 cells)	3.994 ± 0.26 (62 cells)	Mann Whitney test	0.0055	
	Rheo x 4	5.694 ± 0.25 (72 cells)	4.258 ± 0.3 (62 cells)	Mann Whitney test	0.0004	
Average latency at first spike (s)	Rheo x 1	0.227 ± 0.007 (64 cells)	0.194 ± 0.01 (57 cells)	Mann Whitney test	0.0408	Figure 4G
	Rheo x 2	0.069 ± 0.004 (64 cells)	0.062 ± 0.004 (57 cells)	Unpaired t-test	0.2537	
	Rheo x 3	0.042 ± 0.003 (64 cells)	0.037 ± 0.003 (57 cells)	Unpaired t-test	0.1517	
	Rheo x 4	0.03 ± 0.002 (64 cells)	0.026 ± 0.002 (57 cells)	Unpaired t-test	0.2388	
Spike jitter		0.1235 (65 cells)	0.1457 (54 cells)	Mann Whitney test	0.0412	Figure 4H
**Current clamp – clonal parameters for disomic cells**						
Parameter	Genotype	C3 clone	C9 clone	Test	P-value	Figure
V_m (mV)	Disomic	-48 (47 cells)	-53 (57 cells)	Mann Whitney test	0.0812	Figure S2C
Rheobase (pA)	Disomic	28 (39 cells)	17 (33 cells)	Mann Whitney test	0.1824	Figure S2D
**Current clamp – clonal parameters for trisomic cells**						
Parameter	Genotype	C3 clone	C9 clone	Test	P-value	Figure
V_m (mV)	Trisomic	-41.931 ± 1.5 (87 cells)	-46.741 ± 2.6 (27 cells)	Unpaired t-test	0.1157	Figure S2C
Rheobase (pA)	Trisomic	17 (46 cells)	14.25 (16 cells)	Mann Whitney test	0.1206	Figure S2D
**Na^+ channel properties**						
Parameter		Disomic	Trisomic	Test	P-value	Figure
I_{Na} density (pA/ pF)		-54.635 (52 cells)	-42.209 (31 cells)	Mann Whitney test	0.2854	Figure 5A
50% fast inactivation (mV)		-38.83 ± 0.9 (29 cells)	-44.31 ± 0.6 (16 cells)	Unpaired t-test	P<0.0001	Figure 5C
50% slow inactivation (mV)		-42.7 ± 0.4 (29 cells)	-46.1 ± 0.3 (14 cells)	Unpaired t-test	P<0.0001	Figure 5D
**Na^+ channel properties – clonal parameters for disomic cells**						
Parameter	Genotype	C3 clone	C9 clone	Test	P-value	Figure
I_{Na} density (pA/ pF)	Disomic	-65.328 (27 cells)	-42.284 (28 cells)	Mann Whitney test	0.1008	Figure S2F
**Na^+ channel properties – clonal parameters for trisomic cells**						
Parameter	Genotype	C5 clone	C13 clone	Test	P-value	Figure
I_{Na} density (pA/ pF)	Trisomic	-38.188 (14 cells)	-54.403 (17 cells)	Mann Whitney test	0.3163	Figure S2F
**K^+ channel properties – IV curve**						
Parameter	Condition	Disomic	Trisomic	Test	P-value	Figure
K^+ IV curve (current density; pA/ pF)	-110 mV step	-3.58 ± 0.3 (56 cells)	-4.60 ± 0.54 (35 cells)	Unpaired t-test	ns	Figure 6B
	-100 mV step	-2.83 ± 0.24 (56 cells)	-3.76 ± 0.47 (35 cells)	Unpaired t-test	ns	
	-90 mV step	-2.13 ± 0.18 (56 cells)	-2.93 ± 0.39 (35 cells)	Unpaired t-test	ns	
	-80 mV step	-1.49 ± 0.13 (56 cells)	-2.07 ± 0.33 (35 cells)	Unpaired t-test	ns	
	-70 mV step	-0.83 ± 0.08 (56 cells)	-1.21 ± 0.28 (35 cells)	Unpaired t-test	ns	
	-60 mV step	-0.21 ± 0.06 (56 cells)	-0.54 ± 0.23 (35 cells)	Unpaired t-test	ns	
	-50 mV step	0.16 ± 0.07 (56 cells)	-0.05 ± 0.22 (35 cells)	Unpaired t-test	ns	

	-40 mV step	0.53 ± 0.09 (56 cells)	0.42 ± 0.22 (35 cells)	Unpaired t-test	ns	
	-30 mV step	0.88 ± 0.13 (56 cells)	0.91 ± 0.24 (35 cells)	Unpaired t-test	ns	
	-20 mV step	1.48 ± 0.19 (56 cells)	1.63 ± 0.31 (35 cells)	Unpaired t-test	ns	
	-10 mV step	3.07 ± 0.33 (56 cells)	2.79 ± 0.46 (35 cells)	Unpaired t-test	ns	
	0 mV step	6.1 ± 0.56 (56 cells)	4.91 ± 0.68 (35 cells)	Mann-Whitney test	0.0860	
	10 mV step	10.34 ± 0.83 (56 cells)	8.08 ± 0.99 (35 cells)	Mann-Whitney test	0.0241	
	20 mV step	15.52 ± 1.17 (56 cells)	11.99 ± 1.39 (35 cells)	Mann-Whitney test	0.0186	
	30 mV step	21.41 ± 1.56 (56 cells)	16.47 ± 1.82 (35 cells)	Mann-Whitney test	0.0182	
	40 mV step	27.94 ±1.98 (56 cells)	21.72 ± 2.27 (35 cells)	Mann-Whitney test	0.0212	
	50 mV step	34.87 ± 2.3 (56 cells)	27.4 ± 2.83 (35 cells)	Mann-Whitney test	0.0182	
	60 mV step	42.01 ± 2.75 (56 cells)	33.4 ± 3.45 (35 cells)	Mann-Whitney test	0.0171	
	70 mV step	49.44 ± 3.1 (56 cells)	39.47 ± 4.1 (35 cells)	Mann-Whitney test	0.0101	
	80 mV step	56.46 ± 3.39 (56 cells)	45.53 ± 4.76 (35 cells)	Mann-Whitney test	0.0079	
	90 mV step	63.31 ± 3.67 (56 cells)	50.9 ± 5.34 (35 cells)	Mann-Whitney test	0.003	
$I_{k(A\ current)}$ (pA/ pF)	Basal	29.145 (45 cells)	24.736 (31 cells)	Mann-Whitney test	0.0311	Figure 6D
A-current inhibition(% control)	+TEA	16.3 ± 5.5 (24 cells)	38.9 ± 4.9 (23 cells)	Unpaired t-test	0.0038	Figure 6E
	+TEA+4AP	67.8 ± 3.8 (12 cells)	81 ± 3.9 (11 cells)	Unpaired t-test	0.0251	Figure 6E
**K^+ channel properties – clonal parameters for disomic cells**						
Parameter	Genotype	C3 clone	C9 clone	Test	P-value	Figure
K^+ I-V (I density at 90 mV; pA/ pF)	Disomic	64.362 ± 4.9 (28 cells)	62.260 ± 5.3 (28 cells)	Unpaired t-test	0.7618	Figure S2G
$I_{k(A\ current)}$ (pA/ pF)	Disomic	30.578 (22 cells)	29.145 (23 cells)	Mann-Whitney test	0.5474	Figure S2H
**K^+ channel properties – clonal parameters for trisomic cells**						
Parameter	Genotype	C5 clone	C13 clone	Test	P-value	Figure
K^+ I-V (I density at 90 mV; pA/ pF)	Trisomic	40.471 (24 cells)	43.615 (11 cells)	Mann-Whitney test	0.6617	Figure S2G
$I_{k(A\ current)}$ (pA/ pF)	Trisomic	26.2 (15 cells)	26.13 (16 cells)	Mann-Whitney test	0.7518	Figure S2H
**Capacitance – clonal parameters for disomic cells**						
Parameter	Genotype	C3 clone	C9 clone	Test	P-value	Figure
In Cs-based internal	Disomic	86.8 ± 7 (33 cells)	103.23 ± 10.7 (18 cells)	Unpaired t-test	0.1882	Figure S2B
In K-based internal	Disomic	70.559 (56 cells)	77.141 (57 cells)	Mann-Whitney test	0.2181	Figure S2B
**Capacitance – clonal parameters for trisomic cells**						
Parameter	Genotype	C5 clone	C13 clone	Test	P-value	Figure
In Cs-based internal	Trisomic	86.2 ± 8.5 (31 cells)	71.6 ± 6.7 (25 cells)	Unpaired t-test	0.1865	Figure S2B
In K-based internal	Trisomic	62.174 (87 cells)	46.309 (27 cells)	Mann-Whitney test	0.0593	Figure S2B
**Immunofluorescence staining**						
Label		Disomic	Trisomic	Test	P-value	Figure
Synapsin-1/ MAP2		0.32 ± 0.03 (11 stacks)	0.18 ± 0.012 (11 stacks)	Unpaired t-test	0.0002	Figure 2K
Synapsin-1/ SMI-321		1.11 ± 0.06 (11 stacks)	0.68 ± 0.075 (11 stacks)	Unpaired t-test	0.0003	Figure S5A
PSD95/ MAP2		0.2 ± 0.01 (10 stacks)	0.15 ± 0.01 (10 stacks)	Unpaired t-test	0.0011	Figure S5B

PSD95/ SMI-321		0.81 ± 0.07 (10 stacks)	0.46 ± 0.045 (10 stacks)	Unpaired t-test	0.0008	Figure S5B
**RNA expression**						
Gene	Sliding window	Control	Down syndrome	Test	P-Value	Figure
KCND3	5 to 8	7.51 ± SEM 0.29 (3)	6.11 ± SEM 0.43 (3)	Paired t-test	0.010824	Figure 7B
	6 to 9	7.24 ± 0.3 (4)	6.47 ± 0.35 (4)	Paired t-test	0.160675	
	8 to 10	7.14 ± 0.33 (4)	6.86 ± 0.07 (4)	Paired t-test	0.477507	
	9 to 11	7.16 ± 0.35 (4)	6.87 ± 0.07 (4)	Paired t-test	0.470149	
	10 to 12	7.99 ± 0.44 (4)	6.96 ± 0.26 (4)	Paired t-test	0.142504	
	11 to 13	8.26 ± 0.26 (5)	7.13 ± 0.3 (5)	Paired t-test	0.097811	
	12 to 14	8.33 ± 0.27 (5)	7.23 ± 0.3 (5)	Paired t-test	0.104055	
KCND2	5 to 8	9.46 ± SEM 0.18 (3)	8.59 ± SEM 0.77 (3)	Paired t-test	0.278329	Figure 7C
	6 to 9	8.86 ± 0.48 (4)	9.09 ± 0.42 (4)	Paired t-test	0.738238	
	8 to 10	8.28 ± 0.62 (4)	8.83 ± 0.61 (4)	Paired t-test	0.346505	
	9 to 11	8.21 ± 0.56 (4)	8.3 ± 0.48 (4)	Paired t-test	0.910073	
	10 to 12	8.65 ± 0.62 (4)	7.51 ± 0.54 (4)	Paired t-test	0.137881	
	11 to 13	9.23 ± 0.22 (5)	7.63 ± 0.47 (5)	Paired t-test	0.004834	
	12 to 14	9.17 ± 0.2 (5)	7.42 ± 0.48 (5)	Paired t-test	0.005844	
KCNC3	5 to 8	6.39 ± SEM 0.36 (3)	6.15 ± SEM 0.34 (3)	Paired t-test	0.072349	Figure 7D
	6 to 9	6.39 ± 0.25 (4)	6.3 ± 0.26 (4)	Paired t-test	0.711641	
	8 to 10	6.6 ± 0.22 (4)	6.49 ± 0.22 (4)	Paired t-test	0.673465	
	9 to 11	6.41 ± 0.14 (4)	6.3 ± 0.22 (4)	Paired t-test	0.640508	
	10 to 12	6.92 ± 0.25 (4)	6.46 ± 0.15 (4)	Paired t-test	0.100199	
	11 to 13	7.1 ± 0.24 (5)	6.28 ± 0.13 (5)	Paired t-test	0.026606	
	12 to 14	7.09 ± 0.24 (5)	6.33 ± 0.11 (5)	Paired t-test	0.049084	
KCNC4	5 to 8	6.76 ± SEM 0.4 (3)	6.21 ± SEM 0.38 (3)	Paired t-test	0.003055	Figure 7E
	6 to 9	6.93 ± 0.23 (4)	6.44 ± 0.23 (4)	Paired t-test	0.036929	
	8 to 10	7.12 ± 0.16 (4)	6.64 ± 0.17 (4)	Paired t-test	0.041127	
	9 to 11	7.06 ± 0.12 (4)	6.53 ± 0.14 (4)	Paired t-test	0.046405	
	10 to 12	7.23 ± 0.23 (4)	6.38 ± 0.28 (4)	Paired t-test	0.179934	
	11 to 13	7.16 ± 0.2 (5)	6.32 ± 0.2 (5)	Paired t-test	0.090801	
	12 to 14	7.09 ± 0.19 (5)	6.3 ± 0.2 (5)	Paired t-test	0.10978	

Fluorescent in-situ hybridization (FISH)

FISH type	Number	Disomic	Trisomic	Figure
% FISH (chromosome 21)	1	1.6	1.4	Figure S1B
	2	96.8	4.9	
	3	1.6	88.73	
	>3	0	4.9	

% FISH (chromosome 13)	1	0	2.83			
	2	99.2	96.45			
	3	0.8	0			
	>3	0	0.7			
**Whole-cell agonist-activated current densities (pA/ pF)**						
Agonist		Disomic	Trisomic	Test	P-value	Figure
GABA		30.5 ± 17.3 (5 cells)	36.8 ± 14.2 (8 cells)	Unpaired t-test	0.7856	Figure S4D
AMPA (+cyclothiazine)		0.1250 (5 cells)	0.8947 (7 cells)	Mann-Whitney test	0.7432	Figure S4K
NMDA (+glycine)		0.129 ± 0.13 (5 cells)	0.194 ± 0.12 (5 cells)	Unpaired t-test	0.7221	Figure S4K
**mRNA levels (qPCR)**						
Gene		Disomic	Trisomic	Test	P-value	Figure
KCND3/ β-actin (primer set 1)		13.12 ± 0.1 (3 samples)	11.45 ± 0.1 (3 samples)	Paired t-test	0.0005	Figure 7F
KCND3/ β-actin (primer set 2)		12.97 ± 0.1 (3 samples)	10.93 ± 0.1 (3 samples)	Paired t-test	0.0002	Not plotted
KCND3/ β-actin (primer set 3)		12.96 ± 0.1 (3 samples)	10.86 ± 0.1 (3 samples)	Paired t-test	0.0002	Not plotted
**Western blot**						
Protein		Disomic	Trisomic	Test	P-value	Figure
Kv4.3/ β-actin		0.98 ± 0.1 (3 samples)	0.57 ± 0.05 (3 samples)	Paired t-test	0.0468	Figure 7G
**Protein expression (Immunofluorescence)**						
Protein		Disomic	Trisomic	Test	P-value	Figure
Kv4.3		0.19 ± 0.01 (6 stacks)	0.14 ± 0.004 (5 stacks)	Unpaired t-test	0.0245	Figure 7H, S9B
Kv4.2		0.24 ± 0.03 (5 stacks)	0.24 ± 0.03 (5 stacks)	Unpaired t-test	0.8833	Figure S9C
**Pie chart – neuronal identity probed using electrophysiology**						
Parameter		Neuronal	Non-neuronal	Figure		
% neuronal cells		98.2% (228/ 216 cells)	1.8% (4/ 216 cells)	Figure S1F		
**Pie chart – spike type of cells**						
Spike type		Disomic	Trisomic	Figure		
Single spiker		5.1% (5/ 98 cells)	10.1% (10/ 99 cells)	Figure S1G		
Double spiker		2% (2/ 98 cells)	8.1% (8/ 99 cells)	Figure S1G		
Multi Spiker		92.9% (91/ 98 cells)	81.8% (81/ 99 cells)	Figure S1G		
**Pie chart – cells receiving glutamatergic inputs**						
Parameter		Disomic	Trisomic	Figure		
%Cells receiving excitatory postsynaptic inputs in Mg^{2+}		96% (48/ 50)	80% (33/ 41)	Figure 2A		
%Cells receiving excitatory postsynaptic inputs in 0 Mg^{2+}		100% (49/ 49)	90% (53/ 59)	Figure 2F		
%Cells that undergo bursting in 0 Mg^{2+}		100% (37/ 37)	63% (27/ 43)	Figure 3A		
%Cells receiving inhibitory postsynaptic inputs in Mg^{2+}		12% (4/ 34)	2% (1/ 47)	Figure S4A		
