# Supplementary material for: Synaptic and intrinsic membrane defects disrupt early neural network dynamics in Down syndrome
Source: Nat Commun. 2026 Jan 22;17:1287. doi: 10.1038/s41467-025-68048-x (PMC12868644; doi:10.1038/s41467-025-68048-x)
Supplement: Supplementary file 5 — Supplementary data 3 [file 41467_2025_68048_MOESM5_ESM.pdf]

## Supplementary data 3 – Expression of $K^+$ channel, HCN channel, $Na^+$ channel and glutamatergic synapse genes in human cerebellar cortex across development and adulthood

| Group | Gene | ID_REF | Con_CBC_ | Con_CBC_ | Con_CBC_ | Con_CBC_ | Con_CBC_ | Con_CBC_ | Con_CBC_ | Con_CBC_ | Con_CBC_ | Con_CBC_ | DS_CBC_S | DS_CBC_S | DS_CBC_S | DS_CBC_S | DS_CBC_S | DS_CBC_S | DS_CBC_S | DS_CBC_S | DS_CBC_S | DS_CBC_S | Log2FC | pvalue | FDR |
|---|---|---|---|---|---|---|---|---|---|---|---|---|---|---|---|---|---|---|---|---|---|---|---|---|---|
| Potassium | KCNF1 | 2469627 | 4.90699 | 4.68351 | 4.76334 | 4.45581 | 4.31699 | 4.55371 | 4.78726 | 4.68047 | 4.58972 | 4.64983 | 4.98839 | 5.1735 | 4.38568 | 4.94143 | 4.59595 | 4.55785 | 4.75497 | 4.51958 | 5.11389 | 4.67684 | 0.132045 | 0.20265 | 0.496337 |
| Potassium | KCNC4 | 2351294 | 6.58257 | 6.23309 | 5.87348 | 6.19427 | 6.4605 | 5.95529 | 6.49353 | 6.58516 | 7.06777 | 6.79616 | 5.68436 | 5.56505 | 5.74179 | 6.05695 | 6.12598 | 5.93098 | 6.1393 | 6.16104 | 6.04274 | 6.5079 | -0.42857 | 0.002853 | 0.096143 |
| Potassium | KCNK3 | 2473936 | 8.82651 | 8.3614 | 7.18957 | 8.23342 | 7.11408 | 7.62961 | 7.3803 | 7.35489 | 7.29037 | 7.60356 | 6.54458 | 6.61848 | 7.58922 | 6.69164 | 7.2369 | 6.72259 | 6.15597 | 6.45412 | 5.71981 | 6.26681 | -1.09836 | 0.002266 | 0.090195 |
| Potassium | KCNH1 | 2454140 | 8.69404 | 9.01562 | 9.8337 | 9.11538 | 9.24057 | 9.33208 | 9.18692 | 9.09908 | 9.1387 | 8.49883 | 6.64692 | 8.97448 | 8.80529 | 8.6456 | 9.22643 | 8.50499 | 8.67706 | 9.09727 | 8.83685 | 8.56867 | -0.51714 | 0.033603 | 0.229357 |
| Potassium | KCNQ3 | 3154002 | 6.77415 | 6.22844 | 6.95772 | 7.32634 | 7.13384 | 6.9323 | 7.65367 | 6.46727 | 6.81283 | 6.39623 | 7.8075 | 6.88864 | 7.68408 | 6.47972 | 6.67226 | 7.56337 | 7.20317 | 5.84552 | 6.03698 | 6.40974 | -0.00918 | 0.968321 | 0.986451 |
| Potassium | KCNK5 | 2952834 | 5.11625 | 5.38704 | 4.91242 | 5.10537 | 5.07843 | 5.05877 | 4.6204 | 5.17046 | 5.30304 | 4.97748 | 5.20886 | 5.26494 | 4.91993 | 5.44468 | 5.24648 | 5.56482 | 5.11403 | 5.30597 | 5.51122 | 5.76461 | 0.261588 | 0.013877 | 0.162229 |
| Potassium | KCNC2 | 3462567 | 7.12577 | 6.82069 | 6.8185 | 5.59837 | 4.9048 | 4.96953 | 5.67244 | 5.31924 | 6.30229 | 6.02853 | 7.78324 | 7.78416 | 7.2656 | 5.41054 | 5.21841 | 5.21276 | 4.92354 | 5.29715 | 5.34779 | 5.09516 | -0.02218 | 0.91943 | 0.966288 |
| Potassium | KCND3 | 2428119 | 9.36973 | 8.89971 | 9.46858 | 9.78813 | 9.31737 | 10.2561 | 10.0912 | 9.73963 | 9.90285 | 8.49922 | 8.61195 | 8.82663 | 9.89914 | 8.53808 | 9.38155 | 9.03038 | 8.93472 | 8.68182 | 7.81024 | 8.4567 | -0.71613 | 0.018011 | 0.179246 |
| Potassium | KCNB2 | 3103062 | 7.01051 | 6.35234 | 6.04279 | 4.36131 | 4.07367 | 4.11924 | 3.88931 | 4.34188 | 4.14126 | 4.88344 | 8.49581 | 6.79366 | 7.68132 | 4.23215 | 4.43167 | 4.63399 | 4.53009 | 4.93099 | 4.97731 | 4.75709 | 0.624833 | 0.008044 | 0.13204 |
| Potassium | KCNA1 | 3401994 | 10.2306 | 7.5291 | 10.4117 | 11.2542 | 10.7481 | 10.8527 | 11.4883 | 10.9711 | 11.2626 | 10.5149 | 8.56903 | 9.83936 | 10.0285 | 9.84118 | 10.5364 | 9.6221 | 10.5831 | 10.3777 | 7.91669 | 9.4864 | -0.84628 | 0.091477 | 0.347851 |
| Potassium | KCNN2 | 2824581 | 7.271 | 7.88147 | 7.52272 | 7.31379 | 7.84806 | 7.4026 | 7.51864 | 6.56174 | 7.11497 | 6.47708 | 6.94256 | 6.44432 | 6.96507 | 6.63482 | 7.00958 | 5.82912 | 6.49429 | 6.58274 | 6.84685 | 6.39164 | -0.67711 | 0.003445 | 0.101599 |
| Potassium | KCNH3 | 3414029 | 5.90983 | 6.05209 | 6.23359 | 6.43514 | 6.44227 | 6.10283 | 5.64813 | 6.11443 | 6.01493 | 6.01623 | 6.15323 | 5.99355 | 5.98238 | 6.11922 | 6.03475 | 5.98682 | 6.3344 | 6.44276 | 6.89176 | 6.24168 | 0.121108 | 0.395317 | 0.668142 |
| Potassium | KCNJ5 | 3355860 | 4.29597 | 4.63337 | 4.73035 | 4.50473 | 4.36518 | 4.28379 | 4.20929 | 4.34076 | 4.07487 | 4.54462 | 4.43553 | 4.42762 | 4.37396 | 4.72616 | 4.63121 | 4.58073 | 4.61983 | 4.6946 | 4.98678 | 4.53629 | 0.202978 | 0.100886 | 0.363038 |
| Potassium | KCNQ4 | 2332091 | 5.21985 | 6.05541 | 5.72353 | 5.46733 | 5.29777 | 5.847 | 5.26759 | 5.89373 | 5.65184 | 6.2311 | 5.58251 | 5.67312 | 5.53862 | 5.58237 | 5.54287 | 5.68993 | 5.86123 | 5.75812 | 6.0058 | 6.21646 | 0.079588 | 0.432073 | 0.698422 |
| Potassium | KCNH6 | 3730698 | 5.10102 | 5.36072 | 4.94837 | 5.27615 | 5.22589 | 5.03587 | 4.86999 | 4.9624 | 4.75789 | 5.16073 | 5.2022 | 5.26828 | 4.89058 | 5.19373 | 5.00475 | 5.20478 | 5.18524 | 5.24743 | 5.43982 | 5.35761 | 0.129539 | 0.153894 | 0.436814 |
| Potassium | KCNG4 | 3702429 | 3.70588 | 4.55488 | 4.42254 | 4.30784 | 4.48108 | 4.45627 | 4.16421 | 4.30435 | 4.23454 | 4.62387 | 4.38707 | 4.28526 | 4.39545 | 4.49393 | 4.36609 | 4.07595 | 4.50121 | 4.63337 | 5.01423 | 4.70526 | 0.160236 | 0.216752 | 0.511701 |
| Potassium | KCNB1 | 3908901 | 5.8013 | 5.22304 | 5.32394 | 6.9119 | 6.72357 | 5.92272 | 6.41202 | 6.68384 | 7.65395 | 7.55412 | 6.35809 | 5.85952 | 5.85406 | 6.57264 | 6.94951 | 7.27702 | 6.74788 | 6.83172 | 6.21061 | 6.89218 | 0.134283 | 0.599273 | 0.802857 |
| Potassium | KCNK6 | 3832292 | 5.16115 | 5.80347 | 5.83381 | 5.63054 | 5.93761 | 5.76997 | 5.63154 | 5.57567 | 5.17054 | 5.86595 | 6.30971 | 5.85284 | 5.1909 | 5.95933 | 5.70971 | 5.61328 | 5.75895 | 5.69841 | 5.83833 | 5.6737 | 0.122491 | 0.461618 | 0.718432 |
| Potassium | KCNC3 | 3868378 | 8.74718 | 7.89029 | 8.22209 | 8.55341 | 8.53724 | 8.68139 | 7.64179 | 7.95606 | 8.13463 | 8.99285 | 8.00009 | 8.58459 | 8.33821 | 8.22052 | 8.14992 | 8.65538 | 8.12757 | 7.26869 | 7.79393 | 7.5474 | -0.26706 | 0.209614 | 0.503532 |
| Potassium | KCNA6 | 3401953 | 6.92032 | 5.79929 | 5.76354 | 6.50446 | 5.77751 | 5.84432 | 6.10953 | 5.57074 | 5.42583 | 5.88637 | 6.98644 | 6.50264 | 6.35945 | 5.66256 | 5.70853 | 5.72894 | 5.7069 | 5.46216 | 6.01953 | 5.88791 | 0.042315 | 0.787509 | 0.905918 |
| Potassium | KCNJ18 | 3714779 | 4.95519 | 5.00077 | 5.55956 | 6.03398 | 5.89271 | 5.97148 | 6.36389 | 5.88768 | 6.01137 | 5.09919 | 4.0593 | 5.20241 | 5.87554 | 6.0493 | 6.09068 | 6.27221 | 5.73119 | 5.316 | 5.81775 | 6.00399 | -0.03575 | 0.839429 | 0.92957 |
| Potassium | KCNK1 | 2385873 | 10.4477 | 10.1768 | 10.1101 | 10.3512 | 10.3745 | 10.2397 | 9.93577 | 9.94519 | 10.2379 | 10.0195 | 9.01659 | 10.0393 | 10.1664 | 9.86272 | 10.3065 | 9.82751 | 9.97008 | 10.1262 | 8.87148 | 9.7809 | -0.38707 | 0.060974 | 0.291904 |
| Potassium | KCNV2 | 3160229 | 4.65126 | 4.88627 | 5.07678 | 4.91488 | 4.74609 | 4.83619 | 5.14507 | 5.00346 | 4.62417 | 5.42296 | 4.80189 | 5.59304 | 5.02223 | 4.9575 | 5.24261 | 5.72823 | 5.35599 | 5.17854 | 5.28362 | 5.31246 | 0.316898 | 0.018083 | 0.179576 |
| Potassium | KCNV1 | 3148963 | 4.79117 | 4.89958 | 4.95944 | 5.15353 | 4.40568 | 3.95645 | 4.63817 | 4.82442 | 4.34503 | 5.2843 | 5.15731 | 4.95349 | 4.76008 | 5.13778 | 5.05776 | 5.42648 | 4.99231 | 5.27759 | 4.87321 | 5.18026 | 0.35585 | 0.045488 | 0.261417 |
| Potassium | KCNJ9 | 2362860 | 6.94482 | 7.09372 | 7.62151 | 8.35532 | 8.40587 | 8.39836 | 8.22466 | 7.78992 | 8.01496 | 8.00421 | 5.80252 | 7.06118 | 8.28723 | 6.99794 | 7.32052 | 6.78676 | 7.46174 | 7.35026 | 7.10099 | 6.79148 | -0.78927 | 0.005355 | 0.117369 |
| Potassium | KCND2 | 3021009 | 10.3899 | 9.4635 | 10.4935 | 11.356 | 11.0402 | 11.5025 | 11.4547 | 11.7113 | 11.7939 | 10.7584 | 10.4762 | 10.3482 | 10.7567 | 10.1226 | 10.8301 | 10.2913 | 11.0589 | 11.0512 | 9.40683 | 11.0634 | -0.45585 | 0.165215 | 0.451468 |
| Potassium | KCNJ15 | 3920850 | 3.77201 | 3.58761 | 4.64818 | 3.75971 | 4.43808 | 3.81245 | 3.75365 | 3.86018 | 3.78785 | 3.92822 | 3.98351 | 3.76332 | 3.80535 | 4.07197 | 3.55816 | 3.96678 | 4.07055 | 4.38249 | 4.75485 | 4.39584 | 0.140488 | 0.46213 | 0.718726 |
| Potassium | KCNJ3 | 2511153 | 10.1765 | 9.88677 | 10.4083 | 10.944 | 11.0964 | 11.1609 | 11.2276 | 10.757 | 11.3165 | 10.7013 | 10.3544 | 10.4667 | 11.3343 | 10.7483 | 11.103 | 10.5802 | 11.2374 | 10.937 | 10.0445 | 11.2229 | 0.035343 | 0.861949 | 0.940209 |
| Potassium | KCNQ1 | 3317352 | 5.01493 | 5.86213 | 5.20705 | 5.35676 | 5.1472 | 5.17981 | 5.39666 | 5.2877 | 4.89443 | 5.29896 | 5.39653 | 5.1169 | 4.98213 | 5.50022 | 5.20069 | 5.56636 | 5.24031 | 5.38757 | 5.62078 | 5.33436 | 0.070022 | 0.593187 | 0.799154 |
| Potassium | KCNK13 | 3548229 | 5.13903 | 5.70269 | 5 | 5.41619 | 5.33079 | 4.85506 | 5.44128 | 5.46855 | 5.04072 | 5.24355 | 5.0724 | 5.09312 | 4.84025 | 5.47503 | 5.33819 | 5.19989 | 5.27654 | 5.39525 | 5.64342 | 5.34031 | 0.003654 | 0.972094 | 0.987373 |
| Potassium | KCNN1 | 3824666 | 8.88585 | 7.69987 | 6.6167 | 8.04688 | 7.65737 | 7.46506 | 6.9829 | 7.72354 | 7.77285 | 7.69159 | 6.98895 | 8.20305 | 7.01985 | 7.78271 | 7.33467 | 8.10299 | 7.10313 | 6.06184 | 6.09192 | 6.43489 | -0.54186 | 0.117865 | 0.390001 |
| Potassium | KCNK2 | 2379974 | 8.15953 | 6.25953 | 6.72625 | 5.78569 | 5.29556 | 4.46186 | 5.22061 | 4.87438 | 4.37771 | 4.43023 | 8.35656 | 7.58495 | 7.13212 | 5.76542 | 5.76068 | 4.90473 | 5.74052 | 4.65511 | 5.16483 | 4.92129 | 0.439486 | 0.009606 | 0.141374 |
| Potassium | KCNA2 | 2427566 | 7.49631 | 6.98088 | 8.14016 | 8.26107 | 7.78075 | 8.29649 | 8.66575 | 8.45794 | 8.18148 | 8.51476 | 5.69662 | 6.79755 | 8.18888 | 7.31485 | 7.53309 | 7.87885 | 8.275 | 7.60658 | 6.76796 | 7.83749 | -0.68787 | 0.004469 | 0.109575 |
| Potassium | KCNH8 | 2613293 | 7.33053 | 8.71181 | 8.6156 | 8.83085 | 8.40745 | 7.73076 | 6.50315 | 5.79118 | 5.53474 | 5.40732 | 6.0225 | 6.21343 | 8.07938 | 8.12167 | 5.71262 | 7.06737 | 5.6271 | 4.50529 | 5.44919 | 4.63552 | -1.14293 | 0.002039 | 0.087647 |
| Potassium | KCNG1 | 3909442 | 4.53297 | 5.25463 | 5.08093 | 4.83195 | 5.05237 | 5.3902 | 5.16614 | 5.14315 | 5.47711 | 5.5429 | 4.24762 | 5.01342 | 4.77015 | 5.38849 | 5.93445 | 5.58699 | 5.7189 | 4.99743 | 6.04498 | 4.97072 | 0.12008 | 0.461665 | 0.718441 |
| Potassium | KCNC1 | 3322521 | 10.0325 | 10.1697 | 9.54555 | 10.3554 | 10.5422 | 10.027 | 10.0905 | 10.0691 | 10.2304 | 10.0685 | 8.08024 | 9.35767 | 9.99282 | 9.26733 | 9.59157 | 9.6757 | 9.7275 | 9.11449 | 9.26264 | 9.40313 | -0.76578 | 0.003527 | 0.102197 |
| Potassium | KCNQ5 | 2913277 | 5.43038 | 5.38244 | 5.50082 | 5.68998 | 4.72395 | 5.00697 | 5.36986 | 5.13615 | 5.55669 | 6.03289 | 4.78442 | 5.51985 | 6.48645 | 5.08775 | 5.38904 | 5.47767 | 4.7214 | 5.10522 | 4.75479 | 6.08347 | -0.04201 | 0.835684 | 0.928144 |
| Potassium | KCNS3 | 2471384 | 3.9009 | 4.45906 | 4.14981 | 4.23639 | 4.15279 | 4.02782 | 4.19614 | 4.22358 | 3.85845 | 4.35815 | 4.1408 | 4.49052 | 3.88551 | 4.13685 | 4.12716 | 4.53139 | 4.08163 | 4.20692 | 4.27263 | 4.39528 | 0.07056 | 0.381428 | 0.657703 |
| Potassium | KCNH7 | 2584258 | 8.62619 | 8.7461 | 9.55498 | 9.22592 | 8.93802 | 9.24783 | 9.30523 | 7.2067 | 7.8273 | 7.51668 | 8.88198 | 8.84014 | 10.1779 | 8.66399 | 8.83172 | 8.98071 | 7.90998 | 7.59052 | 6.25105 | 8.14404 | -0.19229 | 0.455145 | 0.7138 |
| Potassium | KCNJ14 | 3837825 | 4.21324 | 4.40763 | 4.5305 | 3.86522 | 4.33415 | 4.34019 | 4.09616 | 4.19418 | 3.9615 | 4.25894 | 4.28576 | 4.42954 | 4.2067 | 4.4785 | 4.55408 | 4.6624 | 4.43115 | 4.70076 | 4.37211 | 4.19808 | 0.211737 | 0.043427 | 0.255823 |
| Potassium | KCNK9 | 3155901 | 8.35154 | 7.29319 | 8.30851 | 8.7153 | 7.66299 | 8.32366 | 8.39594 | 9.27022 | 8.29297 | 6.89632 | 8.23211 | 8.242 | 8.09443 | 8.09895 | 8.377 | 7.56847 | 8.55653 | 8.1774 | 7.02978 | 8.28347 | -0.08505 | 0.768728 | 0.896397 |
| Potassium | KCNK12 | 2552051 | 8.84235 | 8.56956 | 9.16427 | 9.44552 | 9.08092 | 9.2354 | 8.88416 | 9.49925 | 9.11341 | 9.36094 | 8.37838 | 8.80975 | 8.89079 | 9.0942 | 8.94534 | 9.28748 | 8.76726 | 8.91158 | 8.87812 | 9.42184 | -0.1811 | 0.050889 | 0.27195 |
| Potassium | KCNK18 | 3266253 | 4.33634 | 4.94408 | 4.29019 | 4.53553 | 4.59318 | 4.40513 | 4.41322 | 4.49638 | 4.19875 | 4.50565 | 4.60922 | 4.19369 | 4.30619 | 4.84279 | 4.37111 | 4.50506 | 4.6089 | 4.66699 | 4.71464 | 4.64563 | 0.074577 | 0.514845 | 0.753045 |
| Potassium | KCNS2 | 3108782 | 5.09042 | 5.95011 | 4.54898 | 5.32391 | 5.488 | 5.11704 | 4.55152 | 4.81142 | 5.71012 | 6.46735 | 5.4618 | 5.20058 | 4.5829 | 6.13008 | 5.31346 | 5.01233 | 5.08981 | 5.13201 | 3.99314 | 5.18462 | -0.19581 | 0.467425 | 0.722567 |
| Potassium | KCNT2 | 2449391 | 5.38719 | 5.12875 | 4.68191 | 4.15352 | 4.36268 | 4.45244 | 4.09174 | 4.04412 | 4.14922 | 4.79613 | 6.45568 | 4.85229 | 6.02249 | 4.34702 | 4.49463 | 4.58273 | 4.24308 | 4.49146 | 5.2062 | 4.66545 | 0.411333 | 0.043156 | 0.25546 |
| Potassium | KCNN3 | 2436826 | 6.45831 | 6.55337 | 5.93919 | 6.59174 | 6.38264 | 6.62443 | 6.46782 | 6.56955 | 6.09548 | 5.66287 | 6.87143 | 6.23386 | 6.58154 | 6.6826 | 7.26659 | 7.48682 | 7.02498 | 7.64685 | 7.1942 | 7.47862 | 0.712209 | 0.004063 | 0.10541 |
| Potassium | KCNN4 | 3864646 | 5.29989 | 5.82525 | 5.69905 | 5.50552 | 5.51174 | 5.2962 | 5.6686 | 5.43014 | 5.40011 | 6.07838 | 5.62789 | 5.59932 | 5.53519 | 5.66774 | 5.46029 | 6.00176 | 5.70555 | 5.87058 | 5.64219 | 5.53846 | 0.093409 | 0.43389 | 0.699666 |
| Potassium | KCNK16 | 2952927 | 3.8396 | 4.48999 | 4.9044 | 4.59791 | 3.88463 | 3.90748 | 4.02171 | 4.54492 | 3.75779 | 4.32526 | 4.81064 | 4.07497 | 3.97206 | 4.47512 | 4.44073 | 4.38007 | 4.60355 | 4.9325 | 4.5427 | 4.8923 | 0.285095 | 0.161611 | 0.446333 |
| Potassium | KCNJ13 | 2603960 | 4.26312 | 3.97099 | 3.56598 | 3.57095 | 3.70371 | 3.74596 | 3.84618 | 3.89794 | 3.55861 | 4.22612 | 3.9648 | 3.40884 | 3.55983 | 4.08886 | 3.96862 | 4.21288 | 4.67758 | 3.89781 | 4.02043 | 4.15676 | 0.160685 | 0.260616 | 0.55408 |
| Potassium | KCNK17 | 2952893 | 5.35736 | 5.13311 | 4.42791 | 4.66587 | 4.92498 | 4.13709 | 4.76484 | 4.87401 | 4.11832 | 4.8349 | 4.86812 | 4.36762 | 4.27336 | 4.89388 | 4.65439 | 4.43713 | 4.83161 | 4.34873 | 4.70603 | 4.83449 | -0.1023 | 0.460605 | 0.717683 |
| Potassium | KCNJ11 | 3364869 | 6.7329 | 4.95488 | 6.83085 | 7.57191 | 7.32391 | 7.26736 | 7.82772 | 7.58469 | 7.64472 | 6.3427 | 5.74312 | 6.64962 | 7.44138 | 6.46663 | 6.99152 | 7.4653 | 6.57751 | 6.17977 | 6.73408 | 6.30018 | -0.35325 | 0.28564 | 0.576634 |
| Potassium | KCNJ2 | 3733275 | 6.48134 | 5.89707 | 6.60178 | 5.92403 | 4.99916 | 4.7851 | 5.33375 | 6.37021 | 5.47509 | 5.32216 | 7.75995 | 5.46953 | 5.4019 | 5.94447 | 5.30594 | 6.75509 | 5.05093 | 5.10593 | 6.2985 | 5.53664 | 0.143919 | 0.667065 | 0.843055 |
| Potassium | KCNJ16 | 3733238 | 5.28062 | 5.38124 | 5.80792 | 6.6992 | 5.66657 | 7.7337 | 7.86875 | 7.22208 | 7.98491 | 6.23974 | 6.17835 | 6.57946 | 5.75568 | 7.17374 | 8.42747 | 7.08716 | 5.68832 | 7.74801 | 8.51654 | 7.85583 | 0.512583 | 0.252366 | 0.546673 |
| Potassium | KCNMA1 | 3296046 | 9.10534 | 8.43896 | 7.59709 | 9.56487 | 8.27073 | 8.98848 | 9.2373 | 8.9269 | 9.01469 | 9.27757 | 7.92915 | 9.23202 | 7.71052 | 9.30837 | 9.1548 | 9.83808 | 9.2059 | 8.48807 | 8.12079 | 8.87325 | -0.0561 | 0.811962 | 0.916537 |
| Potassium | KCNJ6 | 3931495 | 7.76906 | 7.04484 | 8.56799 | 8.37765 | 7.72983 | 8.1939 | 8.13105 | 7.23715 | 7.13794 | 6.28591 | 9.34668 | 8.70086 | 9.41497 | 8.23734 | 8.72416 | 8.86945 | 8.17973 | 8.45803 | 7.45558 | 8.25011 | 0.916159 | 0.002637 | 0.095186 |
| Potassium | KCNJ10 | 2439960 | 8.38991 | 7.85549 | 7.49367 | 9.26231 | 8.99043 | 9.472 | 9.30443 | 9.671 | 8.98284 | 8.98406 | 7.95514 | 7.43002 | 7.37636 | 9.18194 | 9.38882 | 9.48592 | 8.67476 | 8.91469 | 9.84063 | 8.95892 | -0.11989 | 0.453758 | 0.712973 |
| Potassium | KCNJ8 | 3446910 | 5.5321 | 4.27017 | 4.93848 | 5.0103 | 5.16901 | 5.03188 | 5.47133 | 5.38032 | 5.05423 | 4.6676 | 5.18265 | 5.52353 | 5.00453 | 4.91584 | 5.14809 | 5.41058 | 4.98781 | 5.12093 | 5.61264 | 5.20365 | 0.158483 | 0.366423 | 0.645881 |
| Potassium | KCNS1 | 3907151 | 3.61529 | 3.96489 | 3.69995 | 3.84943 | 3.86131 | 3.6382 | 3.8854 | 3.83822 | 3.70669 | 3.67251 | 4.39619 | 3.82554 | 3.74683 | 4.11157 | 3.656 | 3.90173 | 3.83026 | 3.88744 | 3.98989 | 3.96624 | 0.15798 | 0.114184 | 0.38412 |
| Potassium | KCNK10 | 3575241 | 5.83858 | 6.66131 | 7.34493 | 6.98894 | 6.59831 | 7.38099 | 7.16669 | 7.08854 | 6.21241 | 5.3792 | 6.16344 | 5.44726 | 6.33443 | 6.13897 | 6.75811 | 6.11905 | 6.24098 | 6.75631 | 5.94345 | 6.30919 | -0.44487 | 0.088017 | 0.342097 |
| Potassium | KCNK4 | 3334446 | 4.57982 | 4.86738 | 5.86819 | 5.16789 | 4.95839 | 4.97013 | 5.11842 | 5.21006 | 4.86675 | 5.06325 | 4.98791 | 4.68107 | 5.06233 | 5.05243 | 5.13619 | 5.13379 | 5.37628 | 5.37353 | 5.31098 | 5.38337 | 0.08276 | 0.499772 | 0.743349 |
| Potassium | KCNG2 | 3795403 | 4.93737 | 5.1448 | 5.36854 | 5.00956 | 5.56561 | 5.56452 | 5.07254 | 5.17626 | 5.35592 | 5.53184 | 5.78612 | 5.03884 | 5.52388 | 5.26437 | 5.36678 | 5.82656 | 5.02384 | 5.07185 | 5.29109 | 5.49164 | 0.095801 | 0.351655 | 0.635264 |
| Potassium | KCNA10 | 2427538 | 4.06378 | 5.33271 | 4.59406 | 4.3103 | 4.5916 | 4.37836 | 4.32968 | 4.3136 | 4.53848 | 4.39775 | 4.52892 | 4.26727 | 4.17543 | 4.81831 | 4.25156 | 4.69976 | 4.45831 | 5.03574 | 4.8948 | 4.92023 | 0.120001 | 0.513242 | 0.752599 |
| Potassium | KCND1 | 4007689 | 6.8094 | 5.96141 | 7.51795 | 7.42529 | 7.42526 | 7.37759 | 7.0135 | 7.13653 | 7.24316 | 7.82272 | 5.49344 | 6.76092 | 7.87067 | 6.08533 | 6.94389 | 6.27478 | 7.22946 | 6.06182 | 6.61593 | 5.85143 | -0.65451 | 0.042763 | 0.254244 |
| Potassium | KCNH4 | 3757690 | 4.37548 | 4.88552 | 4.86829 | 4.76711 | 4.90906 | 4.53689 | 4.79828 | 4.80636 | 4.45266 | 4.79664 | 4.85875 | 4.91468 | 4.86343 | 4.83089 | 4.78499 | 4.7551 | 4.80826 | 4.80559 | 5.20224 | 4.94689 | 0.157453 | 0.094314 | 0.352465 |
| Potassium | KCNJ1 | 3397774 | 3.8211 | 4.09562 | 4.16417 | 4.24877 | 4.16877 | 4.07251 | 3.69409 | 4.12107 | 3.98377 | 3.95114 | 4.09468 | 3.72122 | 4.05839 | 4.01876 | 4.07774 | 4.22714 | 4.32678 | 4.09587 | 4.09788 | 4.04418 | 0.044163 | 0.631996 | 0.822263 |
| Potassium | KCNK7 | 3377737 | 3.95928 | 4.3349 | 4.88395 | 4.26148 | 5.03334 | 4.73307 | 4.50169 | 4.10348 | 4.60989 | 4.95515 | 4.11767 | 4.29282 | 4.52898 | 4.26521 | 4.13521 | 4.39512 | 4.26214 | 4.73283 | 4.50898 | 4.20602 | -0.19313 | 0.196561 | 0.489436 |
| Potassium | KCNK15 | 3886598 | 5.2519 | 4.87994 | 5.58809 | 5.61194 | 4.75902 | 5.17434 | 5.58475 | 5.97667 | 5.19506 | 4.69968 | 5.94815 | 6.18041 | 5.50379 | 5.81827 | 5.64943 | 5.83913 | 6.07618 | 5.94743 | 5.60165 | 5.79623 | 0.563928 | 0.003669 | 0.104194 |
| Potassium | KCNH5 | 3567873 | 3.69901 | 4.05123 | 3.82235 | 3.81558 | 3.65466 | 3.68948 | 3.78913 | 3.73795 | 3.69964 | 4.2849 | 3.84008 | 3.75557 | 3.56301 | 3.81633 | 3.95997 | 4.08756 | 3.93446 | 3.86458 | 3.87251 | 3.84091 | 0.029105 | 0.745769 | 0.88448 |
| Potassium | KCNQ2 | 3913821 | 7.54558 | 7.75548 | 8.12485 | 7.42943 | 7.92675 | 8.20139 | 7.69432 | 7.41344 | 7.62545 | 7.72078 | 7.28994 | 7.47531 | 7.97143 | 7.06634 | 7.36354 | 8.05716 | 8.0314 | 7.20725 | 8.06937 | 7.23169 | -0.1674 | 0.13756 | 0.41575 |
| Potassium | KCNG3 | 2550175 | 3.83126 | 4.77727 | 4.67847 | 4.23353 | 4.25285 | 4.05354 | 3.89117 | 5.4027 | 4.08388 | 4.50593 | 4.41822 | 4.26617 | 3.93443 | 4.12722 | 3.93031 | 4.46562 | 3.98717 | 4.29119 | 4.14921 | 4.60969 | -0.15314 | 0.379374 | 0.656094 |
| Potassium | KCNA7 | 3867669 | 4.32459 | 4.22531 | 3.99339 | 4.5388 | 3.99628 | 3.95966 | 4.19874 | 4.51414 | 4.08552 | 4.23674 | 4.48056 | 4.30949 | 4.28086 | 4.32497 | 4.11116 | 4.39638 | 4.0944 | 4.25537 | 4.33289 | 4.51555 | 0.102846 | 0.190406 | 0.481338 |
| Potassium | KCNU1 | 3093781 | 3.8819 | 4.67182 | 4.4511 | 3.84397 | 4.38926 | 3.96386 | 3.97613 | 3.96952 | 4.20224 | 4.94089 | 4.46948 | 4.17658 | 4.00761 | 3.90001 | 3.72811 | 4.85247 | 4.37484 | 4.17254 | 4.56311 | 4.22271 | 0.017677 | 0.923185 | 0.967086 |
| Potassium | KCNH2 | 3079202 | 7.82256 | 7.7224 | 7.6466 | 7.63191 | 7.8841 | 7.77671 | 8.07502 | 8.15249 | 8.50425 | 8.42969 | 7.17825 | 7.43391 | 7.60597 | 7.56608 | 7.38569 | 7.34987 | 7.56087 | 7.49621 | 7.21604 | 7.66128 | -0.51916 | 0.001437 | 0.080361 |
| Potassium | KCNA5 | 3402039 | 4.53105 | 4.73508 | 5.25132 | 4.60999 | 5.12644 | 4.74853 | 4.86233 | 4.65645 | 4.51413 | 5.09219 | 4.48786 | 4.88729 | 4.89195 | 4.77545 | 4.54937 | 4.71002 | 4.79949 | 5.25864 | 5.09308 | 4.99537 | 0.032101 | 0.788731 | 0.906503 |
| Potassium | KCNJ4 | 3960605 | 6.34042 | 6.07768 | 5.47025 | 5.85372 | 6.10288 | 4.72294 | 5.44935 | 5.75547 | 5.04853 | 5.67039 | 6.40401 | 6.085 | 5.6838 | 5.81055 | 6.06094 | 5.69453 | 5.85239 | 5.99497 | 6.03495 | 6.67635 | 0.380586 | 0.023358 | 0.20044 |
| Potassium | KCNA4 | 3367580 | 4.07692 | 4.8185 | 4.96259 | 4.97561 | 4.46972 | 4.08661 | 4.27735 | 5.21973 | 4.24957 | 4.7847 | 5.70467 | 5.03771 | 5.53606 | 5.72441 | 5.34863 | 4.96118 | 4.9182 | 5.48468 | 4.8455 | 5.41836 | 0.70581 | 0.000295 | 0.052205 |
| Potassium | KCNA3 | 2427619 | 5.41066 | 6.35233 | 8.40448 | 6.51498 | 7.30303 | 7.03199 | 7.25972 | 6.18337 | 7.30503 | 6.48871 | 6.64946 | 6.56955 | 7.80695 | 6.39401 | 7.0904 | 7.41532 | 7.27975 | 7.37409 | 5.74487 | 6.56931 | 0.063941 | 0.809402 | 0.915341 |
| Potassium | HCN1 | 2855963 | 10.4408 | 9.34681 | 10.5036 | 11.0155 | 10.2846 | 10.9845 | 11.0231 | 11.147 | 11.0951 | 10.4586 | 9.98733 | 10.3427 | 10.4761 | 10.4789 | 10.6607 | 10.5053 | 10.5534 | 9.93731 | 8.48018 | 10.2838 | -0.45939 | 0.16462 | 0.450685 |
| Potassium | HCN2 | 3815045 | 5.8553 | 6.02531 | 5.39004 | 6.38358 | 6.679 | 6.07162 | 6.40731 | 6.71448 | 6.98489 | 7.1997 | 5.88604 | 6.24562 | 6.05048 | 6.2198 | 6.32317 | 6.95046 | 6.63548 | 6.38755 | 6.16831 | 6.62685 | -0.02175 | 0.90025 | 0.957094 |
| Potassium | HCN3 | 2360818 | 7.19556 | 6.76939 | 6.55855 | 6.26345 | 5.83428 | 6.39777 | 5.38168 | 6.58177 | 6.71774 | 6.5309 | 6.57959 | 6.72342 | 7.16443 | 6.13977 | 7.21808 | 6.9683 | 7.21691 | 7.02867 | 6.93839 | 7.66712 | 0.541359 | 0.046911 | 0.263487 |
| Potassium | HCN4 | 3632424 | 6.11822 | 5.86743 | 5.76432 | 6.12182 | 5.66634 | 5.22677 | 5.56308 | 5.72685 | 5.89348 | 6.62743 | 5.78577 | 5.54577 | 5.87423 | 5.27856 | 6.08808 | 5.76446 | 5.22017 | 5.6543 | 4.67938 | 6.07402 | -0.2611 | 0.160694 | 0.445494 |
| Sodium | SCN1A | 2585322 | 6.18733 | 6.25488 | 6.27489 | 6.02553 | 5.98809 | 7.06024 | 7.41775 | 7.04089 | 7.1269 | 6.87379 | 6.62607 | 6.61135 | 6.69027 | 6.48984 | 6.99184 | 6.67896 | 6.59748 | 6.99316 | 6.53531 | 8.01483 | 0.197882 | 0.36046 | 0.642041 |
| Sodium | SCN2A | 2513471 | 10.4258 | 11.4027 | 11.7509 | 11.7868 | 12.0013 | 11.9413 | 11.9629 | 11.7439 | 11.5927 | 10.7427 | 9.89516 | 11.0861 | 11.769 | 11.1661 | 11.5723 | 11.3797 | 11.6029 | 11.8779 | 11.2524 | 11.4379 | -0.23115 | 0.104881 | 0.369429 |
| Sodium | SCN3A | 2584957 | 6.11343 | 6.84199 | 7.76991 | 7.49557 | 7.09507 | 7.94217 | 7.98857 | 7.35174 | 7.33358 | 6.60368 | 7.63316 | 7.51278 | 8.2843 | 6.89309 | 8.18647 | 7.73262 | 7.7722 | 8.42362 | 7.30464 | 7.98469 | 0.519186 | 0.055698 | 0.282453 |
| Sodium | SCN4A | 3766549 | 4.55726 | 4.74858 | 4.48518 | 4.78357 | 4.66389 | 5.1416 | 4.71984 | 4.80439 | 4.42543 | 4.77068 | 4.30486 | 4.6303 | 4.33094 | 5.12163 | 4.67435 | 4.62732 | 4.44139 | 4.73745 | 4.95304 | 4.87741 | -0.04017 | 0.686574 | 0.853656 |
| Sodium | SCN5A | 2669641 | 4.49117 | 5.87622 | 4.79089 | 4.91645 | 4.50205 | 4.68723 | 4.37649 | 4.90749 | 4.31603 | 4.9067 | 5.22917 | 4.80429 | 4.62255 | 5.032 | 4.55129 | 4.88299 | 5.13897 | 5.23141 | 5.44849 | 5.43001 | 0.260045 | 0.210178 | 0.50418 |
| Sodium | SCN8A | 3414969 | 7.82391 | 7.60184 | 9.44439 | 9.13482 | 9.20652 | 9.32929 | 9.41904 | 9.14548 | 9.16614 | 7.97662 | 8.44901 | 8.16303 | 9.13115 | 8.21342 | 8.70229 | 9.45341 | 9.4096 | 9.12777 | 8.79182 | 9.31905 | 0.05125 | 0.809586 | 0.915432 |
| Sodium | SCN9A | 2585400 | 4.17683 | 4.75811 | 4.16816 | 4.06942 | 4.02056 | 4.21085 | 4.04048 | 4.28975 | 3.95755 | 4.18879 | 4.35468 | 4.30224 | 4.09323 | 4.15819 | 4.06269 | 4.18213 | 4.3075 | 4.42345 | 4.5057 | 4.4968 | 0.100611 | 0.262927 | 0.556169 |
| Sodium | SCN10A | 2669732 | 3.94407 | 4.75744 | 4.22199 | 3.98749 | 4.12511 | 4.0148 | 3.86966 | 4.16434 | 4.06804 | 4.36741 | 4.09855 | 3.91523 | 3.72459 | 4.103 | 4.18437 | 4.21799 | 4.10053 | 4.23746 | 4.29274 | 4.513 | -0.01329 | 0.90967 | 0.961766 |
| Sodium | SCN11A | 2669803 | 3.94453 | 4.42812 | 4.30263 | 3.98506 | 4.01577 | 4.16788 | 3.85281 | 4.0651 | 3.85936 | 4.30126 | 4.25527 | 4.02139 | 3.92742 | 4.37578 | 4.04098 | 4.30951 | 4.24705 | 4.27551 | 4.46015 | 4.47282 | 0.146336 | 0.188522 | 0.480249 |
| Sodium | SCN1B | 3830051 | 9.95822 | 9.60907 | 9.09504 | 9.42627 | 9.90435 | 9.59526 | 9.60445 | 9.72084 | 10.1624 | 10.0775 | 8.23507 | 9.24148 | 8.85725 | 9.2538 | 9.39817 | 8.94937 | 9.61184 | 9.17547 | 9.07331 | 9.18642 | -0.61712 | 0.004011 | 0.104946 |
| Sodium | SCN2B | 3393652 | 7.79876 | 6.59991 | 7.37471 | 8.0079 | 8.81494 | 9.15326 | 9.01634 | 8.68184 | 9.31452 | 9.64719 | 6.1738 | 7.50504 | 8.15182 | 7.45866 | 8.6186 | 8.77526 | 8.59404 | 8.93772 | 8.29742 | 9.78911 | -0.21079 | 0.411862 | 0.68089 |
| Sodium | SCN3B | 3395691 | 8.58969 | 8.29535 | 8.16496 | 8.45406 | 6.43153 | 6.585 | 7.73433 | 7.92237 | 6.15473 | 6.2976 | 9.00091 | 8.51624 | 9.11373 | 6.87825 | 7.43052 | 7.34609 | 7.16863 | 8.15435 | 5.18672 | 5.33779 | -0.04964 | 0.866268 | 0.942438 |
| Sodium | SCN4B | 3393622 | 7.45468 | 6.92573 | 6.68475 | 7.8805 | 7.80714 | 7.66308 | 8.09904 | 8.10452 | 8.69453 | 8.20546 | 5.86161 | 6.95466 | 7.27591 | 7.26443 | 7.09739 | 7.55968 | 8.18445 | 7.1975 | 8.4193 | 8.36054 | -0.3344 | 0.128568 | 0.404565 |
| Glutamate | GRM1 | 2929571 | 9.4005 | 8.93756 | 9.33096 | 9.87273 | 9.33907 | 9.45584 | 9.45505 | 9.39024 | 9.31717 | 8.75371 | 8.54255 | 8.57368 | 9.4127 | 8.7766 | 9.15061 | 8.91006 | 8.83241 | 8.60576 | 7.6943 | 8.44508 | -0.63091 | 0.002796 | 0.095869 |
| Glutamate | GRM2 | 2623308 | 7.0303 | 6.64405 | 4.77514 | 5.99169 | 4.76192 | 5.94841 | 5.16727 | 6.20791 | 6.57896 | 6.84066 | 6.05116 | 5.55185 | 5.4625 | 5.53487 | 4.88681 | 5.01223 | 5.23427 | 5.21363 | 5.085 | 5.23653 | -0.66775 | 0.020211 | 0.188129 |
| Glutamate | GRM3 | 3011180 | 8.77526 | 8.3347 | 7.15041 | 9.08949 | 7.93624 | 9.0802 | 9.38841 | 9.1612 | 8.70451 | 8.71776 | 8.05065 | 8.20293 | 7.06206 | 8.5521 | 8.81003 | 9.03714 | 7.6739 | 8.62103 | 8.50397 | 8.62977 | -0.31946 | 0.157478 | 0.441728 |
| Glutamate | GRM4 | 2950970 | 8.85547 | 8.34437 | 9.80059 | 9.61621 | 9.83761 | 10.4625 | 9.18205 | 9.60193 | 9.80978 | 9.99181 | 8.5283 | 9.53667 | 9.52133 | 9.32322 | 9.72678 | 9.40532 | 9.29478 | 9.36504 | 9.24442 | 8.85229 | -0.27042 | 0.219473 | 0.513798 |
| Glutamate | GRM5 | 3385834 | 7.9371 | 7.15736 | 7.10817 | 7.58622 | 7.17376 | 7.62756 | 8.1265 | 6.82273 | 6.27834 | 6.68866 | 8.50303 | 7.40929 | 7.6332 | 7.37907 | 8.16145 | 7.79801 | 6.92066 | 7.49412 | 7.96973 | 7.09321 | 0.385537 | 0.14148 | 0.419506 |
| Glutamate | GRM6 | 2889848 | 4.69617 | 5.12094 | 4.20384 | 4.7607 | 4.29923 | 4.44326 | 4.60675 | 4.84085 | 4.7442 | 4.82147 | 4.33468 | 4.734 | 4.4275 | 4.60406 | 4.41108 | 4.44034 | 4.31346 | 4.47293 | 4.56403 | 4.68893 | -0.15464 | 0.045462 | 0.261417 |
| Glutamate | GRM7 | 2609061 | 5.87762 | 5.29747 | 4.5719 | 5.49864 | 4.83301 | 5.06465 | 5.24669 | 5.97306 | 5.48247 | 6.92368 | 5.04877 | 5.90606 | 4.49084 | 6.0485 | 6.05823 | 5.94469 | 5.57719 | 5.33169 | 4.94253 | 5.45997 | 0.003928 | 0.98874 | 0.994404 |
| Glutamate | GRM8 | 3071063 | 4.12905 | 4.86893 | 4.91509 | 4.19557 | 4.06021 | 4.181 | 4.90716 | 4.84036 | 4.13665 | 5.31784 | 5.08134 | 4.13638 | 4.85554 | 4.57169 | 4.16933 | 3.99599 | 4.77746 | 4.37298 | 4.30938 | 4.88226 | -0.03995 | 0.798318 | 0.91043 |
| Glutamate | GRIK1 | 3928211 | 9.48318 | 7.76307 | 7.12669 | 8.3454 | 8.18392 | 7.36361 | 7.60703 | 8.50561 | 7.81434 | 8.24859 | 9.59039 | 9.71032 | 8.43763 | 9.46746 | 8.90093 | 9.51595 | 8.79462 | 7.77535 | 6.69438 | 8.44762 | 0.689321 | 0.073349 | 0.317501 |
| Glutamate | GRIK2 | 2918982 | 10.7422 | 11.4999 | 11.2632 | 11.0696 | 11.098 | 11.2724 | 11.0105 | 11.1744 | 10.8702 | 10.232 | 10.6209 | 11.1837 | 11.5905 | 11.1116 | 10.8433 | 10.2991 | 10.7809 | 11.1411 | 10.5732 | 10.3623 | -0.17258 | 0.152438 | 0.434968 |
| Glutamate | GRIK3 | 2406926 | 7.51475 | 6.66044 | 6.59741 | 7.49802 | 7.02636 | 7.74512 | 8.24994 | 7.67963 | 8.19896 | 8.13402 | 6.94572 | 7.40932 | 6.33968 | 7.45126 | 7.21151 | 7.51413 | 8.00695 | 7.01443 | 6.4029 | 7.59512 | -0.34136 | 0.135163 | 0.412132 |
| Glutamate | GRIK4 | 3352618 | 8.16836 | 7.08804 | 7.09744 | 8.03031 | 7.63305 | 7.91488 | 8.27741 | 8.08175 | 7.73961 | 8.06134 | 7.16022 | 7.95535 | 7.17484 | 7.76985 | 7.88755 | 8.32961 | 7.25483 | 6.94079 | 7.62465 | 7.38907 | -0.26054 | 0.259217 | 0.552986 |
| Glutamate | GRIK5 | 3863380 | 8.42683 | 8.05099 | 8.45636 | 8.35972 | 8.4496 | 8.09419 | 7.94203 | 7.79887 | 8.39858 | 8.06708 | 8.4193 | 8.56588 | 8.90035 | 8.09547 | 7.94424 | 7.78733 | 8.22033 | 7.67775 | 7.83823 | 7.34225 | -0.12531 | 0.379704 | 0.656278 |
| Glutamate | GRIA1 | 2836242 | 10.7258 | 10.6949 | 10.4752 | 10.9146 | 10.8447 | 10.865 | 11.2872 | 10.5706 | 10.0366 | 10.1124 | 11.0417 | 10.4926 | 10.5107 | 10.5995 | 11.2595 | 11.2522 | 9.90599 | 10.8789 | 11 | 10.928 | 0.134209 | 0.5392 | 0.765735 |
| Glutamate | GRIA2 | 2749222 | 9.92934 | 10.1394 | 10.8087 | 10.2485 | 10.5659 | 10.8226 | 10.3318 | 9.92246 | 10.1803 | 9.64577 | 10.6849 | 10.2545 | 11.3583 | 10.1017 | 10.2309 | 10.2836 | 10.3531 | 10.4572 | 10.4155 | 10.1036 | 0.164853 | 0.246237 | 0.541346 |
| Glutamate | GRIA3 | 3989448 | 7.60344 | 6.89258 | 6.9687 | 8.68555 | 7.22262 | 7.42346 | 8.34409 | 7.84798 | 7.77692 | 7.5379 | 7.47549 | 7.1054 | 7.11401 | 7.744 | 7.31734 | 8.02739 | 7.44099 | 6.53873 | 6.13471 | 6.87106 | -0.45341 | 0.085438 | 0.338378 |
| Glutamate | GRIA4 | 3347118 | 9.62383 | 9.32346 | 9.60623 | 9.84958 | 9.38352 | 9.92919 | 10.2047 | 9.78255 | 9.37871 | 9.27365 | 9.99234 | 9.90186 | 10.268 | 9.64479 | 10.0325 | 9.96407 | 9.85651 | 9.94583 | 9.5366 | 9.79295 | 0.258003 | 0.048328 | 0.265899 |
| Glutamate | GRIN1 | 3195238 | 7.83587 | 7.28308 | 7.79107 | 7.2722 | 7.62376 | 7.99729 | 7.20801 | 7.19543 | 7.96648 | 7.95484 | 7.18485 | 7.58984 | 7.71079 | 6.9536 | 6.845 | 7.14136 | 7.4056 | 6.58934 | 7.10867 | 6.86995 | -0.4729 | 0.012023 | 0.15411 |
| Glutamate | GRIN2A | 3679812 | 7.36373 | 7.12196 | 9.36197 | 9.20891 | 8.25839 | 9.00261 | 8.89762 | 8.59891 | 8.39299 | 6.97396 | 7.01845 | 8.0213 | 8.68074 | 7.8778 | 8.66673 | 8.16746 | 8.124 | 8.12714 | 6.98749 | 8.22496 | -0.3285 | 0.278768 | 0.571152 |
| Glutamate | GRIN2B | 3445326 | 7.15861 | 5.69366 | 8.15981 | 6.72227 | 5.52149 | 6.43217 | 7.57654 | 6.65252 | 7.24711 | 6.11883 | 9.56274 | 7.49662 | 7.91067 | 5.81927 | 6.32161 | 5.6113 | 6.01889 | 6.17724 | 4.52846 | 5.80329 | -0.20329 | 0.68339 | 0.85216 |
| Glutamate | GRIN2C | 3770422 | 8.4899 | 7.9469 | 8.24562 | 8.66415 | 8.63937 | 8.7965 | 7.96634 | 8.66512 | 8.38281 | 8.45265 | 6.41348 | 7.57825 | 8.3475 | 7.66116 | 7.75627 | 7.78053 | 8.07061 | 7.70628 | 8.13961 | 7.36402 | -0.74317 | 0.006143 | 0.121193 |
| Glutamate | GRIN2D | 3837759 | 5.83275 | 5.44855 | 5.36826 | 5.70068 | 5.28806 | 5.27184 | 5.57643 | 5.46706 | 5.58157 | 5.71445 | 6.13382 | 6.14335 | 5.77141 | 5.41607 | 5.45691 | 5.85607 | 5.2327 | 5.0873 | 5.18372 | 5.40185 | 0.043355 | 0.758672 | 0.891537 |
| Glutamate | GRIN3A | 3218151 | 4.12668 | 4.72033 | 3.87035 | 3.77858 | 3.69653 | 3.60486 | 3.81998 | 3.98959 | 3.44673 | 3.8911 | 4.79754 | 4.04153 | 3.91118 | 3.77341 | 3.89346 | 3.7912 | 3.59408 | 3.76732 | 3.97585 | 3.95655 | 0.055739 | 0.658596 | 0.837737 |
| Glutamate | GRID1 | 3298557 | 6.91956 | 6.90694 | 5.746 | 6.75825 | 6.57 | 6.5097 | 6.73775 | 6.45169 | 6.50906 | 7.1925 | 6.57643 | 6.65243 | 6.82386 | 6.21405 | 6.38858 | 5.95271 | 6.06659 | 6.69533 | 5.93382 | 6.51643 | -0.24812 | 0.183261 | 0.47414 |
| Glutamate | GRID2 | 2736060 | 11.5224 | 11.2944 | 10.6291 | 11.2454 | 10.9324 | 11.2327 | 11.3397 | 11.3302 | 11.2938 | 11.2014 | 11.1562 | 11.2469 | 11.0173 | 11.3334 | 11.3315 | 11.448 | 10.9354 | 11.0201 | 11.4558 | 11.0383 | -0.00386 | 0.968486 | 0.986466 |
| Glutamate | SYP | 4007899 | 10.5499 | 9.45981 | 9.57019 | 10.6273 | 10.5731 | 10.3881 | 9.54535 | 10.4626 | 10.6917 | 11.6725 | 10.3717 | 9.84601 | 10.1372 | 9.72189 | 10.1942 | 10.8408 | 10.5247 | 10.1276 | 10.396 | 10.1266 | -0.12539 | 0.609807 | 0.80876 |
| Glutamate | SYN1 | 4007126 | 10.131 | 10.1355 | 8.7335 | 10.1572 | 9.64393 | 9.74819 | 9.79106 | 10.3338 | 10.508 | 10.3286 | 8.63097 | 9.61449 | 8.90125 | 9.32292 | 9.38671 | 9.5356 | 9.58745 | 9.70629 | 8.73912 | 9.17232 | -0.69137 | 0.006549 | 0.12338 |
| Glutamate | DLG4 | 3743393 | 10.2692 | 9.71334 | 9.38457 | 10.8315 | 9.9982 | 10.1717 | 10.2689 | 11.1726 | 10.6911 | 11.0295 | 9.50624 | 10.362 | 9.82187 | 9.96568 | 9.84972 | 9.53961 | 10.0535 | 9.70751 | 8.92606 | 9.86433 | -0.59341 | 0.040009 | 0.246509 |
| Glutamate | NLGN1 | 2652801 | 9.66134 | 8.95397 | 10.5488 | 10.4268 | 9.55232 | 10.1362 | 10.5493 | 9.90002 | 9.82081 | 8.34852 | 10.0011 | 10.0078 | 10.8184 | 9.43144 | 10.1293 | 8.85915 | 9.42993 | 9.90314 | 8.3817 | 9.30327 | -0.16329 | 0.602151 | 0.804136 |
| Glutamate | SLC17A7 | 3867842 | 12.034 | 11.5777 | 11.1303 | 12.2157 | 11.6409 | 11.3614 | 11.5476 | 12.0447 | 11.803 | 11.3312 | 9.71332 | 11.1208 | 11.212 | 10.9152 | 11.1125 | 10.8904 | 11.0031 | 10.7686 | 9.53144 | 10.2316 | -1.01875 | 0.002907 | 0.097261 |
| Glutamate | SHANK1 | 3868587 | 9.17993 | 8.40669 | 8.77442 | 8.9168 | 9.08431 | 9.1486 | 9.10367 | 8.69062 | 8.82309 | 8.60207 | 8.48808 | 8.69559 | 8.78498 | 8.68967 | 8.61499 | 9.29408 | 8.75907 | 8.44891 | 8.4761 | 8.64436 | -0.18344 | 0.086503 | 0.340066 |
| Glutamate | SHANK2 | 3380365 | 8.68045 | 7.77537 | 8.29598 | 8.3461 | 7.92538 | 8.58798 | 7.91412 | 7.30505 | 7.95506 | 8.38975 | 7.98398 | 8.1431 | 8.52089 | 8.0068 | 7.81707 | 8.33968 | 8.10958 | 7.25479 | 6.86622 | 7.25593 | -0.28772 | 0.121144 | 0.394624 |
