# Supplementary material for: Synaptic and intrinsic membrane defects disrupt early neural network dynamics in Down syndrome
Source: Nat Commun. 2026 Jan 22;17:1287. doi: 10.1038/s41467-025-68048-x (PMC12868644; doi:10.1038/s41467-025-68048-x)
Supplement: Supplementary file 6 — Supplementary data 4 [file 41467_2025_68048_MOESM6_ESM.pdf]

Supplementary data 4 - List of differentially expressed in the dorsolateral prefrontal cortex (DFC)

Altered expression of genes with a fold-change (FC) >=1.3 and p<0.01

GENE LIST	p<0.01	|FC|>1.3 & p<0.01
GRIN2A	GRIN2A	GRIN2A
KCNF1	KCNF1	KCNF1
KCNC4	KCNC4	KCNC4
KCNK3	KCNK3	KCNK3
KCNH1	KCNH1	KCNH1
KCNQ3	KCNQ3	KCNQ3
GRIN1	GRIN1	GRIK3
GRIK3	GRIK3	KCNK5
KCNK5	KCNK5	KCNC2
KCNC2	KCNC2	GRIK4
GRIK4	GRIK4	KCND3
KCND3	KCND3	KCNB2
KCNB2	KCNB2	KCNA1
KCNA1	KCNA1	
GRIN2D		
KCNN2		
KCNH3		
SCN4A		
KCNJ5		
KCNQ4		
KCNH6		
KCNG4		
SCN5A		
KCNB1		
KCNK6		
SCN1A		
KCNC3		
KCNA6		
KCNJ18		
KCNK1		
KCNV2		
KCNV1		
KCNJ9		
GRM2		
KCND2		
KCNJ15		
SLC17A7		
KCNJ3		
KCNQ1		
SCN3B		
GRM3		
GRIA4		
KCNK13		
KCNN1		
GRM7		
KCNK2		
GRID1		
GRIN2B		
SCN9A		
KCNA2		
SYN1		
KCNH8		
SCN11A		
GRM8		
KCNG1		
HCN3		
DLG4		
KCNC1		
SHANK2		
SCN2A		
KCNQ5		
KCNS3		
GRIA1		
KCNH7		
KCNJ14		
SCN8A		
KCNK9		
GRIA3		
KCNK12		
KCNK18		
KCNS2		
GRIK1		
GRIA2		
KCNT2		
HCN4		
KCNN3		
SCN4B		
HCN1		
GRIK2		
KCNN4		
GRM6		
SHANK1		
KCNK16		
KCNJ13		
KCNK17		
KCNJ11		
GRM4		
KCNJ2		
KCNJ16		
KCNMA1		
SCN2B		
KCNJ6		
KCNJ10		
NLGN1		
KCNJ8		
KCNS1		
GRIN3A		
SCN3A		
KCNK10		
KCNK4		
KCNG2		
SCN10A		
KCNA10		
SCN1B		
SYP		
GRID2		
GRIK5		
KCND1		
HCN2		
KCNH4		
KCNJ1		
KCNK7		
KCNK15		
KCNH5		
KCNQ2		
GRM1		
KCNG3		
KCNA7		
KCNU1		
KCNH2		
GRIN2C		
KCNA5		
GRM5		
KCNJ4		
KCNA4		
KCNA3		
