# Supplementary material for: Synaptic and intrinsic membrane defects disrupt early neural network dynamics in Down syndrome
Source: Nat Commun. 2026 Jan 22;17:1287. doi: 10.1038/s41467-025-68048-x (PMC12868644; doi:10.1038/s41467-025-68048-x)
Supplement: Supplementary file 7 — Supplementary data 5 [file 41467_2025_68048_MOESM7_ESM.pdf]

Supplementary data 5 - Heatmap showing expression fold Change >= 1.3 and p<0.01

Group	Gene	ID_REF	Con_DFC_S	Con_DFC_S	Con_DFC_S	Con_DFC_S	Con_DFC_S	Con_DFC_S	Con_DFC_S	Con_DFC_S	Con_DFC_S	Con_DFC_S	Con_DFC_S	Con_DFC_S	DS_DFC_St	DS_DFC_St	DS_DFC_St	DS_DFC_St	DS_DFC_St	DS_DFC_St	DS_DFC_St	DS_DFC_St	DS_DFC_St	DS_DFC_St	DS_DFC_St	DS_DFC_St	Log2FC	pvalue	FDR
Glutamate	GRIN2A	3679812	6.10714	6.77851	10.3608	9.36816	9.13758	9.69644	10.5161	9.75684	9.42188	9.723	8.63459	9.76603	4.92043	5.93043	8.92859	9.70293	8.46645	9.14424	8.69894	9.20708	8.11799	8.37273	8.64369	8.54121	-0.8827	0.000489	0.049618
Potassium	KCNF1	2469627	6.39239	6.5778	7.13753	6.75006	7.49189	6.45035	7.58204	7.16916	6.74213	7.20943	7.28432	6.63193	5.61782	5.53562	6.02626	6.98758	5.93758	6.55342	7.24814	6.98819	5.58086	6.06083	6.36482	5.16142	-0.77971	0.000873	0.05716
Potassium	KCNC4	2351294	6.27012	6.44236	7.55261	6.80305	6.93213	7.18034	7.3342	6.64058	7.77345	6.86981	7.18699	7.0007	5.71625	5.95901	6.96811	6.67611	6.14736	6.76083	6.51775	6.68477	5.53909	6.43926	6.40295	6.45597	-0.64324	0.002234	0.077798
Potassium	KCNK3	2473936	5.89775	6.32896	7.69524	6.46529	6.99961	6.44722	7.25075	6.91869	6.74702	6.94704	6.45617	6.55479	6.03326	5.59506	6.01718	6.64304	6.93064	6.23675	6.30418	6.56561	5.76706	5.88345	5.77188	5.69554	-0.60541	0.003012	0.084342
Potassium	KCNH1	2454140	5.16101	4.84467	7.96398	8.33697	8.67737	9.19243	9.78577	9.63783	9.65335	9.59559	9.51188	9.79924	4.59501	5.0093	6.66936	8.96639	7.74935	8.75632	9.17215	9.17752	8.31263	8.37919	9.00603	8.7575	-0.63411	0.003431	0.088655
Potassium	KCNQ3	3154002	9.53081	10.035	10.7814	9.6019	10.3239	9.93733	10.0966	10.2831	9.71771	9.31478	9.1142	9.56213	9.48693	9.18744	10.2387	9.70064	10.0051	9.86856	8.54037	9.57086	8.54479	8.8483	8.73257	9.36948	-0.51709	0.003722	0.090748
Glutamate	GRIK3	2406926	10.1052	9.15836	9.97605	8.40295	7.96809	8.49264	8.67691	8.67644	8.65685	9.00167	8.81143	8.08814	10.1411	9.42466	8.97416	8.15813	8.19844	8.14684	7.22883	7.67089	6.76241	6.76452	7.45928	6.50194	-0.88196	0.004334	0.096319
Potassium	KCNK5	2952834	4.47159	5.45324	4.95615	5.55506	5.16784	4.93428	4.76816	5.18213	4.81433	5.14154	5.2192	4.9394	5.13816	5.64674	4.94418	5.26268	5.64688	5.75236	5.79016	5.14018	5.94944	5.61094	5.4494	5.62798	0.446348	0.005048	0.102608
Potassium	KCNC2	3462567	8.57515	8.03813	10.1676	9.30907	11.0614	8.83374	11.0401	11.0446	10.408	10.1779	9.76954	10.6529	6.53161	6.77728	8.93292	9.75532	9.84525	9.11858	10.0797	10.4501	8.2694	9.19981	10.2208	9.4255	-0.87266	0.005393	0.104738
Glutamate	GRIK4	3352618	7.27962	7.12795	8.43943	7.80409	8.16993	8.44398	8.82329	7.98322	8.23127	7.97187	8.18898	8.36823	6.13846	6.35946	7.83931	7.56769	8.27987	8.23591	7.82709	7.78749	6.65364	8.3401	7.58998	7.76225	-0.53755	0.005813	0.107475
Potassium	KCND3	2428119	7.33767	7.11952	8.07453	6.63761	7.14137	6.70106	8.16815	8.52788	8.56639	8.75992	7.28641	8.52539	5.97753	5.45133	6.91	6.87384	6.66378	6.99447	6.93166	7.5868	6.32823	6.76769	8.03281	7.44016	-0.9073	0.006331	0.110913
Potassium	KCNB2	3103062	7.29499	8.45801	8.03907	7.1275	6.91407	6.95024	8.25618	8.15796	7.25266	8.13209	7.16823	8.41306	7.72697	7.89809	8.0219	7.39271	6.00691	6.91937	6.7248	6.95143	6.19913	6.79339	6.70716	6.55303	-0.6891	0.008261	0.120192
Potassium	KCNA1	3401994	4.91811	5.32056	9.10055	6.34283	8.58671	7.22598	10.1692	9.75657	9.5546	10.2357	9.77793	9.63716	5.05551	4.61726	6.09208	8.19831	6.95175	7.82181	7.63647	7.62237	6.9652	7.14306	7.6498	7.40626	-1.4555	0.008499	0.120808
