# Supplementary material for: Synaptic and intrinsic membrane defects disrupt early neural network dynamics in Down syndrome
Source: Nat Commun. 2026 Jan 22;17:1287. doi: 10.1038/s41467-025-68048-x (PMC12868644; doi:10.1038/s41467-025-68048-x)
Supplement: Supplementary file 9 — Reporting Summary [file 41467_2025_68048_MOESM9_ESM.pdf]

nature portfolio

Corresponding author(s): Saad Hannan and Trevor Smart

Last updated by author(s): 17/11/25

Reporting Summary

Nature Portfolio wishes to improve the reproducibility of the work that we publish. This form provides structure for consistency and transparency in reporting. For further information on Nature Portfolio policies, see our Editorial Policies and the Editorial Policy Checklist.

Statistics

For all statistical analyses, confirm that the following items are present in the figure legend, table legend, main text, or Methods section.

n/a	Confirmed	
[ ]	[x]	The exact sample size (*n*) for each experimental group/condition, given as a discrete number and unit of measurement
[ ]	[x]	A statement on whether measurements were taken from distinct samples or whether the same sample was measured repeatedly
[ ]	[x]	The statistical test(s) used AND whether they are one- or two-sided<br>*Only common tests should be described solely by name; describe more complex techniques in the Methods section.*
[x]	[ ]	A description of all covariates tested
[ ]	[x]	A description of any assumptions or corrections, such as tests of normality and adjustment for multiple comparisons
[ ]	[x]	A full description of the statistical parameters including central tendency (e.g. means) or other basic estimates (e.g. regression coefficient) AND variation (e.g. standard deviation) or associated estimates of uncertainty (e.g. confidence intervals)
[ ]	[x]	For null hypothesis testing, the test statistic (e.g. *F*, *t*, *r*) with confidence intervals, effect sizes, degrees of freedom and *P* value noted<br>*Give P values as exact values whenever suitable.*
[x]	[ ]	For Bayesian analysis, information on the choice of priors and Markov chain Monte Carlo settings
[x]	[ ]	For hierarchical and complex designs, identification of the appropriate level for tests and full reporting of outcomes
[x]	[ ]	Estimates of effect sizes (e.g. Cohen's *d*, Pearson's *r*), indicating how they were calculated

Our web collection on statistics for biologists contains articles on many of the points above.

Software and code

Policy information about availability of computer code

Data collection	Clampex Ver 10.2 - for patch clamp recordings<br>Maestro Pro AxIS Navigator 1.5.2 - for MEA recordings<br>Olympus FV acquisition software- For image acquisition
Data analysis	Maestro Pro Axion Biosystems - for analysing MEA data<br>AxIS Navigator Axion Biosystems- for analysing MEA data<br>Neural Metric Tool Axion Biosystems- for analysing MEA data<br>Clampfit 10.6 - for analysing electrophysiological data<br>Imaris ver 9.9.1 - for image analysis<br>WinWCP 5.7.0 - for analysing electrophysiological data<br>WinEDR 4.0.2 - for analysing electrophysiological data<br>Instat 3.06 - for statistical analysis<br>Prism ver 9 - for statistical analysis<br>Origin 6 - for generating graphs and statistical tests<br>OriginPro 2021b - for generating graphs and statistical tests.<br>ImageJ FiJi 1.54- for analysing Western Blot images

For manuscripts utilizing custom algorithms or software that are central to the research but not yet described in published literature, software must be made available to editors and reviewers. We strongly encourage code deposition in a community repository (e.g. GitHub). See the Nature Portfolio guidelines for submitting code & software for further information.

Data

Policy information about availability of data

All manuscripts must include a data availability statement. This statement should provide the following information, where applicable:

- Accession codes, unique identifiers, or web links for publicly available datasets
- A description of any restrictions on data availability
- For clinical datasets or third party data, please ensure that the statement adheres to our policy

All data are available in the main text, supplementary materials or on servers discussed in the manuscript. Additional requests for data, code, and materials used in the manuscript will be made available upon reasonable request by the corresponding authors under suitable materials transfer agreements (MTAs).

Research involving human participants, their data, or biological material

Policy information about studies with human participants or human data. See also policy information about sex, gender (identity/presentation), and sexual orientation and race, ethnicity and racism.

Reporting on sex and gender	Uses previously reported lines and results from both genders - Included in manuscript figures
Reporting on race, ethnicity, or other socially relevant groupings	N/A - uses previously reported datasets and lines
Population characteristics	N/A
Recruitment	N/A - uses previously reported datasets and lines
Ethics oversight	N/A - uses previously reported datasets and lines. Briefly, primary skin fibroblasts and used and stored under UK-Human Tissue Authority License 12199, and ethical approval from the Ethical Committee of the NELHA (North East London Health Authority), P/03/086.

Note that full information on the approval of the study protocol must also be provided in the manuscript.

Field-specific reporting

Please select the one below that is the best fit for your research. If you are not sure, read the appropriate sections before making your selection.

[x] Life sciences [ ] Behavioural & social sciences [ ] Ecological, evolutionary & environmental sciences

For a reference copy of the document with all sections, see nature.com/documents/nr-reporting-summary-flat.pdf

Life sciences study design

All studies must disclose on these points even when the disclosure is negative.

Sample size	Sample sizes for our experiments were based on many previous studies where adequate statistical power was achieved at P= 0.05.
Data exclusions	All data included unless otherwise specified
Replication	Number of replicates for each experiment is stated in the figure legends and in supplementary data table
Randomization	Systematic randomization was not used in this study as it is impractical for such in vitro experiments that invariably have the control and drug-test designed into the same experiment
Blinding	Some experiments were carried out blind as stated in the manuscript. For others iInvestigators were not blind to study conditions since this is difficult to achieve when the electrophysiological signals are so easily recognised, ie, GABA-activated currents

Reporting for specific materials, systems and methods

We require information from authors about some types of materials, experimental systems and methods used in many studies. Here, indicate whether each material, system or method listed is relevant to your study. If you are not sure if a list item applies to your research, read the appropriate section before selecting a response.

Materials & experimental systems

n/a	Involved in the study
	[x] Antibodies
	[x] Eukaryotic cell lines
[x]	Palaeontology and archaeology
[x]	Animals and other organisms
[x]	Clinical data
[x]	Dual use research of concern
[x]	Plants

Methods

n/a	Involved in the study
[x]	ChIP-seq
[x]	Flow cytometry
[x]	MRI-based neuroimaging

Antibodies

Antibodies used

Anti-MAP2 antibody Abcam ab5392
Anti-Nestin antibody Abcam ab6320
Anti-Neurofilament Marker (pan axonal, cocktail, SMI-312) antibody Biolegend 837904
Anti-PAX6 antibody Biolegend 901301
Anti-PSD95 antibody Cell Signaling 3450
Anti-SOX2 antibody Santa Cruz sc-17320
Anti-Synapsin-1 antibody Cell Signaling 5297
Anti-TUBB3 antibody Biolegend 802001
Donkey anti-Mouse IgG (H + L) ThermoFisher Scientific A-21202
Donkey anti-Mouse IgG (H + L) ThermoFisher Scientific A-31570
Donkey anti-Mouse IgG (H + L) ThermoFisher Scientific A-31571
Donkey anti-Rabbit IgG (H + L) ThermoFisher Scientific A-21206
Donkey anti-Rabbit IgG (H + L) ThermoFisher Scientific A-31572
Donkey anti-Rabbit IgG (H + L) ThermoFisher Scientific A-31573
Donkey anti-Goat IgG (H + L) ThermoFisher Scientific A-21432
Goat anti-Chicken IgY (H + L) ThermoFisher Scientific A-21103

Validation

Validation stated in manufacturers' websites and previous publications.

Eukaryotic cell lines

Policy information about cell lines and Sex and Gender in Research

Cell line source(s)	Previously published
Authentication	Previously published - tested for 2 or 3 copies of chromosome 21 using FISH
Mycoplasma contamination	All lines tested negative for mycoplasma contamination
Commonly misidentified lines (See ICLAC register)	N/A

Plants

Seed stocks	N/A
Novel plant genotypes	N/A
Authentication	N/A
