# Supplementary material for: Synaptic and intrinsic membrane defects disrupt early neural network dynamics in Down syndrome
Source: Nat Commun. 2026 Jan 22;17:1287. doi: 10.1038/s41467-025-68048-x (PMC12868644; doi:10.1038/s41467-025-68048-x)
Supplement: Supplementary file 4 — Supplementary data 2 [file 41467_2025_68048_MOESM4_ESM.pdf]

Supplementary data 2 – Expression of K^{+} channel, HCN channel, Na^{+} channel and glutamatergic synapse genes in human dorsolateral prefrontal cortex across development and adulthood

Group	Gene	ID_REF	Con_DFC_S	Con_DFC_S	Con_DFC_S	Con_DFC_S	Con_DFC_S	Con_DFC_S	Con_DFC_S	Con_DFC_S	Con_DFC_S	Con_DFC_S	Con_DFC_S	Con_DFC_S	DS_DFC_St	DS_DFC_St	DS_DFC_St	DS_DFC_St	DS_DFC_St	DS_DFC_St	DS_DFC_St	DS_DFC_St	DS_DFC_St	DS_DFC_St	DS_DFC_St	DS_DFC_St	Log2FC	pvalue	FDR
Potassium	KCNF1	2469627	6.39239	6.5778	7.13753	6.75006	7.49189	6.45035	7.58204	7.16916	6.74213	7.20943	7.28432	6.63193	5.61782	5.53562	6.02626	6.98758	5.93758	6.55342	7.24814	6.98819	5.58086	6.06083	6.36482	5.16142	-0.77971	0.000873	0.05716
Potassium	KCNC4	2351294	6.27012	6.44236	7.55261	6.80305	6.93213	7.18034	7.3342	6.64058	7.77345	6.86981	7.18699	7.0007	5.71625	5.95901	6.96811	6.67611	6.14736	6.76083	6.51775	6.68477	5.53909	6.43926	6.40295	6.45597	-0.64324	0.002234	0.077798
Potassium	KCNK3	2473936	5.89775	6.32896	7.69524	6.46529	6.99961	6.44722	7.25075	6.91869	6.74702	6.94704	6.45617	6.55479	6.03326	5.59506	6.01718	6.64304	6.93064	6.23675	6.30418	6.56561	5.76706	5.88345	5.77188	5.69554	-0.60541	0.003012	0.084342
Potassium	KCNH1	2454140	5.16101	4.84467	7.96398	8.33697	8.67737	9.19243	9.78577	9.63783	9.65335	9.59559	9.51188	9.79924	4.59501	5.0093	6.66936	8.96639	7.74935	8.75632	9.17215	9.17752	8.31263	8.37919	9.00603	8.7575	-0.63411	0.003431	0.088655
Potassium	KCNQ3	3154002	9.53081	10.035	10.7814	9.6019	10.3239	9.93733	10.0966	10.2831	9.71771	9.31478	9.1142	9.56213	9.48693	9.18744	10.2387	9.70064	10.0051	9.86856	8.54037	9.57086	8.54479	8.8483	8.73257	9.36948	-0.51709	0.003722	0.090748
Potassium	KCNK5	2952834	4.47159	5.45324	4.95615	5.55506	5.16784	4.93428	4.76816	5.18213	4.81433	5.14154	5.2192	4.9394	5.13816	5.64674	4.94418	5.26268	5.64688	5.75236	5.79016	5.14018	5.94944	5.61094	5.4494	5.62798	0.446348	0.005048	0.102608
Potassium	KCNC2	3462567	8.57515	8.03813	10.1676	9.30907	11.0614	8.83374	11.0401	11.0446	10.408	10.1779	9.76954	10.6529	6.53161	6.77728	8.93292	9.75532	9.84525	9.11858	10.0797	10.4501	8.2694	9.19981	10.2208	9.4255	-0.87266	0.005393	0.104738
Potassium	KCND3	2428119	7.33767	7.11952	8.07453	6.63761	7.14137	6.70106	8.16815	8.52788	8.56639	8.75992	7.28641	8.52539	5.97753	5.45133	6.91	6.87384	6.66378	6.99447	6.93166	7.5868	6.32823	6.76769	8.03281	7.44016	-0.9073	0.006331	0.110913
Potassium	KCNB2	3103062	7.29499	8.45801	8.03907	7.1275	6.91407	6.95024	8.25618	8.15796	7.25266	8.13209	7.16823	8.41306	7.72697	7.89809	8.0219	7.39271	6.00691	6.91937	6.7248	6.95143	6.19913	6.79339	6.70716	6.55303	-0.6891	0.008261	0.120192
Potassium	KCNA1	3401994	4.91811	5.32056	9.10055	6.34283	8.58671	7.22598	10.1692	9.75657	9.5546	10.2357	9.77793	9.63716	5.05551	4.61726	6.09208	8.19831	6.95175	7.82181	7.63647	7.62237	6.9652	7.14306	7.6498	7.40626	-1.4555	0.008499	0.120808
Potassium	KCNN2	2824581	5.76807	5.6336	6.65	7.41097	6.6624	6.29742	6.78941	6.33577	6.15071	6.5101	6.19584	6.10648	6.1827	5.40431	6.11585	6.33526	6.19469	6.08924	6.29291	6.21134	5.5021	6.12535	5.14661	6.26827	-0.38685	0.010592	0.12799
Potassium	KCNH3	3414029	5.99398	6.22568	8.88799	9.09199	8.78702	9.076	8.76493	8.538	9.00205	8.81209	8.58579	7.78912	6.20229	6.67408	7.99192	8.36271	8.18963	8.62902	8.16188	8.56407	8.41898	8.03332	8.02807	7.84853	-0.37085	0.01363	0.13863
Potassium	KCNJ5	3355860	4.03973	4.22356	5.4636	4.81094	4.76937	4.27727	4.31859	4.65457	4.12746	4.30713	4.607	4.53849	4.26957	4.41793	6.05625	4.75126	4.5783	4.66752	5.02338	4.54585	5.06309	4.89779	4.53983	4.94632	0.301615	0.01493	0.142999
Potassium	KCNQ4	2332091	5.37082	5.79189	6.08963	5.63986	5.74856	5.89283	5.60941	6.31125	6.21947	6.21783	6.01727	6.01096	5.7405	6.09462	6.14349	5.88629	6.20538	6.13072	6.29645	6.2543	6.4057	6.01136	5.91395	6.33895	0.208494	0.015795	0.145955
Potassium	KCNH6	3730698	4.66758	5.42541	4.89781	4.71567	4.8218	4.95472	4.8561	5.71466	4.91909	4.68073	5.06488	5.0801	5.50544	5.38668	5.019	5.10241	5.1832	5.13796	5.76063	5.28123	5.9622	5.21707	5.41572	5.07046	0.353621	0.015879	0.146272
Potassium	KCNG4	3702429	4.00174	4.26955	3.99932	4.39467	3.98626	4.00194	4.21675	4.22862	4.06527	4.38053	4.20104	4.30394	4.54873	4.17846	4.27021	4.31306	4.36501	4.04328	4.41263	4.33338	4.57235	4.58502	4.37449	4.21395	0.180078	0.016029	0.1469
Potassium	KCNB1	3908901	7.50216	7.67993	9.48992	7.80112	8.14683	9.41386	9.215	10.0115	10.0887	10.322	10.2006	9.67495	6.62173	7.06071	8.61611	9.32716	7.94211	9.76275	7.73146	8.65548	7.92891	8.19586	8.81625	9.11	-0.81484	0.020183	0.157982
Potassium	KCNK6	3832292	5.45846	5.69028	5.31998	5.83304	5.62915	4.98594	5.61223	5.66351	5.90187	5.33638	5.43658	6.07713	5.93049	5.74023	5.91585	5.57025	5.55037	5.79994	6.21538	5.83344	6.41827	5.78455	6.30043	5.71717	0.319318	0.020237	0.157982
Potassium	KCNC3	3868378	6.07765	5.99374	7.10386	6.15191	6.32686	6.80882	6.36701	6.92247	7.5648	6.94921	7.67163	6.32918	5.74989	5.88837	6.81824	6.67995	5.83066	6.65024	6.02637	6.62533	6.54087	6.10658	6.07792	6.27696	-0.41631	0.021258	0.160944
Potassium	KCNA6	3401953	8.74685	8.62606	8.51044	6.77563	7.6463	7.27269	8.06484	8.12117	7.8397	7.90997	8.07937	7.43402	7.71673	8.02049	8.21834	8.05834	7.49612	7.51554	6.12154	7.22786	6.79594	6.59949	7.05702	6.83531	-0.61369	0.025882	0.174481
Potassium	KCNJ18	3714779	4.21054	4.43643	4.69056	5.3093	5.21089	5.2281	4.3923	5.17609	4.72283	4.27831	3.99881	4.29507	4.59554	4.3879	5.03912	5.75468	5.75664	5.01665	5.59089	4.89223	4.42932	4.98049	5.71064	5.61619	0.485088	0.026798	0.17661
Potassium	KCNK1	2385873	4.81435	5.37774	6.78925	7.44149	7.48198	7.85939	8.78784	8.09363	8.42582	8.49997	8.55724	8.20549	5.24725	5.45566	5.11989	7.48914	6.80608	7.94348	8.21315	8.06119	6.11943	7.81154	7.60092	7.43009	-0.58636	0.026832	0.176772
Potassium	KCNV2	3160229	3.91152	4.08216	3.99078	4.35908	5.08136	3.90377	4.11573	4.35009	4.87205	4.26044	4.35375	5.29547	4.75655	4.78524	4.31046	4.16371	5.00528	4.38498	4.94975	4.44555	5.63912	5.43114	5.04294	4.52533	0.405321	0.027206	0.178363
Potassium	KCNV1	3148963	7.39177	8.80558	8.87307	7.58168	8.85703	7.80768	10.051	8.98062	7.35341	8.33424	7.03883	8.44388	7.13418	7.61	8.32239	8.20273	7.38585	7.59808	7.20752	8.12121	7.7732	6.6569	7.54993	6.57629	-0.78171	0.028081	0.18063
Potassium	KCNJ9	2362860	5.12646	5.11401	7.58456	6.89999	6.36893	7.54458	6.32764	7.33497	7.79055	7.66058	6.80631	6.641	5.3571	4.79284	6.5059	7.39839	6.43042	7.04988	5.72298	6.57318	6.74709	5.78239	6.58179	6.63116	-0.46887	0.031625	0.191594
Potassium	KCND2	3021009	9.22493	9.3322	9.82566	7.62101	8.67702	6.98905	9.54199	9.63165	8.41833	9.19921	9.35886	9.256	7.37911	8.39236	10.0118	9.59653	8.37318	7.33498	7.89388	8.67966	6.11802	7.06496	8.39534	6.85224	-0.91532	0.032769	0.194627
Potassium	KCNJ15	3920850	3.68414	3.87722	3.99445	4.00429	3.84277	4.52958	3.86595	4.00223	3.75265	3.58865	4.2327	3.96151	3.91852	4.04886	3.69457	4.26631	4.18566	4.41789	4.51089	4.29951	5.12239	4.51927	3.85726	4.51475	0.334978	0.039622	0.21206
Potassium	KCNJ3	2511153	8.02533	7.70903	10.0372	8.85414	9.52921	7.91527	10.6413	10.2092	10.3159	10.5105	10.3729	10.3455	8.1382	5.81645	8.84476	10.0384	9.152	8.88743	9.49355	9.81049	6.43083	8.68008	9.47915	8.6654	-0.91906	0.041369	0.216165
Potassium	KCNQ1	3317352	5.08106	5.29606	5.20588	5.95714	4.93459	5.34104	4.91977	5.32952	4.81648	4.95943	5.22423	5.2471	5.53904	5.5925	5.19115	5.34105	5.59371	5.38278	5.86164	5.10791	6.29366	5.34261	5.49316	5.90596	0.361073	0.043804	0.222032
Potassium	KCNK13	3548229	5.07252	5.15526	5.42475	5.50605	5.38362	5.48564	5.38184	5.77938	5.56926	5.7566	5.8277	5.31078	5.30397	5.22714	5.49829	5.59132	5.92495	5.81962	5.77901	5.62656	6.72684	5.76599	5.47324	5.91733	0.250072	0.052054	0.237072
Potassium	KCNN1	3824666	7.95465	8.07019	9.59698	8.94447	9.65952	9.52367	9.06503	9.58652	10.0254	9.75794	8.69914	8.97837	7.39042	7.45441	8.56644	8.96669	8.82045	9.70593	9.08411	9.62377	8.74239	9.68915	9.03647	8.91626	-0.32212	0.056705	0.24661
Potassium	KCNK2	2379974	9.93713	10.4298	8.1919	7.63705	8.30001	6.84135	8.64486	7.61173	6.59881	7.31042	7.29171	6.99251	9.51144	10.7823	8.84345	6.62306	7.36436	6.72901	8.59496	6.83516	6.80736	6.87983	6.47065	6.35931	-0.3322	0.057254	0.246868
Potassium	KCNA2	2427566	5.45086	6.01602	7.28337	5.66456	6.46068	6.63883	7.63418	8.2783	8.72928	8.40902	8.3813	7.915	5.49413	6.08414	6.13001	7.21847	5.74045	6.44258	7.30722	7.92207	6.31459	5.85265	7.74339	6.9398	-0.63933	0.069662	0.268335
Potassium	KCNH8	2613293	7.16894	5.86093	5.26796	5.47834	4.72965	6.46183	4.60787	7.08096	6.90232	7.85711	6.02584	7.78793	6.08527	5.31636	5.33379	4.86477	5.09767	5.80387	6.23679	4.56626	6.9987	6.28239	5.33998	5.69133	-0.63438	0.076688	0.28021
Potassium	KCNG1	3909442	6.94194	5.80693	8.19273	7.84907	7.69286	7.14005	8.08582	7.04396	6.29069	6.77017	6.49621	5.92367	6.81023	5.84328	7.70511	8.09855	7.32066	7.22339	6.59589	7.28317	5.19079	7.11008	5.81072	5.53251	-0.30914	0.08581	0.293109
Potassium	KCNC1	3322521	7.27216	7.49133	8.38474	6.87473	7.28234	8.08656	7.87467	8.41902	9.36669	8.93058	8.25456	8.27431	7.41094	7.35075	7.58966	7.47947	7.39062	7.47421	7.74612	8.11946	8.06528	7.73225	8.13911	8.38268	-0.3026	0.093522	0.303226
Potassium	KCNQ5	2913277	10.224	10.3649	10.1589	8.9503	9.60988	8.25552	10.4055	11.0994	10.7466	11.0803	10.3249	10.91	9.37794	10.3581	9.56251	9.93637	8.78101	9.60324	10.2151	10.0091	8.1235	9.18675	10.6343	9.36106	-0.58177	0.108821	0.325924
Potassium	KCNS3	2471384	3.92421	4.41761	4.75124	5.1423	4.55525	4.48064	5.24547	5.04232	5.05343	5.11389	5.12684	5.10946	4.22037	4.4172	4.27994	4.69568	4.90273	4.48084	5.3974	4.97878	4.65914	4.23726	4.78709	4.78104	-0.1771	0.116681	0.336077
Potassium	KCNH7	2584258	8.98665	8.92019	9.64137	9.15069	10.0022	8.47106	10.3444	9.18306	9.09686	9.20419	8.54454	9.41667	9.23791	9.83449	9.36684	9.393	9.07856	9.05503	8.50868	9.10434	7.49514	8.2388	8.49229	7.98833	-0.43071	0.12572	0.34844
Potassium	KCNJ14	3837825	4.13845	4.2178	3.91418	4.64762	4.51029	4.28371	4.39041	4.2458	3.9172	4.16134	4.57889	4.39878	4.14506	4.32056	4.29606	4.35304	4.36434	4.82078	4.50089	4.21962	5.03539	4.52447	4.39794	4.58332	0.17975	0.13374	0.359692
Potassium	KCNK9	3155901	7.12914	7.8981	6.72561	5.46404	5.83201	5.28677	6.56303	6.42167	5.83894	6.25052	5.14555	6.15384	6.60623	5.79365	6.32985	5.84008	6.20614	5.16572	5.44499	6.24731	5.64947	5.4879	6.09916	5.49614	-0.36188	0.139759	0.366834
Potassium	KCNK12	2552051	7.00697	8.18371	9.36392	8.19552	9.30788	8.9486	8.49905	9.08695	8.57604	8.90364	8.66785	8.73893	6.79342	6.9728	7.93785	8.82846	8.61517	9.0907	8.77222	9.05127	9.01571	8.28914	8.48783	8.24003	-0.28204	0.151499	0.380485
Potassium	KCNK18	3266253	4.12228	4.50657	4.59478	5.03741	4.23068	4.7772	4.20001	4.68026	4.41671	4.21705	4.84122	4.97686	4.66916	4.82623	4.62756	4.68346	4.74705	4.59618	4.66993	4.41782	5.79255	4.69773	4.6359	4.8783	0.22007	0.152909	0.381715
Potassium	KCNS2	3108782	5.6604	5.28876	8.07143	7.11395	7.14295	7.00335	7.93104	7.94734	8.21879	7.91787	8.32892	7.9647	5.39036	4.65973	7.11648	7.9347	7.38688	6.55658	8.45131	8.07771	6.96784	7.60424	7.8095	7.54889	-0.25711	0.163509	0.394711
Potassium	KCNT2	2449391	7.58355	7.54459	8.70879	9.74663	9.40544	8.15629	8.66043	7.33435	7.3252	7.27275	6.67297	6.99958	8.11984	7.87844	8.6777	8.88783	9.13652	7.72018	8.05137	7.32869	6.98999	6.47142	7.02531	6.83375	-0.19079	0.171042	0.4028
Potassium	KCNN3	2436826	6.28061	6.11699	7.98987	7.66328	7.37857	8.22161	6.85579	7.54032	8.44965	7.65581	7.3554	7.93081	6.90607	5.55518	7.27295	7.66071	7.32668	8.19526	7.43742	8.51811	8.83868	7.96599	7.96408	8.2604	0.205235	0.182032	0.416258
Potassium	KCNN4	3864646	5.308	5.62945	5.71828	5.85336	5.20437	5.5423	5.33774	5.44895	5.69317	5.15469	5.75169	6.10998	5.64317	5.48275	5.72352	5.7501	5.46724	5.87025	6.07628	5.20798	6.81977	5.29113	5.54083	5.87329	0.166194	0.202151	0.43996
Potassium	KCNK16	2952927	4.04994	5.26486	4.21601	4.50187	4.52817	4.25753	3.9137	4.4477	3.97819	4.25524	4.89707	4.87382	4.65345	4.29074	4.03536	4.75252	5.11462	5.01378	4.82991	4.28806	5.26124	4.23568	4.51142	4.8903	0.224415	0.243675	0.482563
Potassium	KCNJ13	2603960	3.42682	3.70203	4.51746	4.85921	4.05756	3.9816	4.17642	4.13128	3.93724	3.67246	4.33092	3.80724	4.02643	4.81678	5.34066	4.07137	3.67794	3.97862	4.9818	4.17167	4.06276	3.89299	3.8803	4.0724	0.19779	0.253918	0.491276
Potassium	KCNK17	2952893	4.12397	4.34401	4.50707	4.64418	4.2197	5.01979	4.78411	4.6138	4.44356	4.42253	5.36686	4.68073	4.04976	4.38079	4.33335	4.6783	4.69548	5.02816	5.23285	4.74405	5.30045	4.84161	4.61489	4.88903	0.134868	0.270495	0.506714
Potassium	KCNJ11	3364869	5.68011	5.68726	5.92006	4.80607	4.92097	5.12845	4.94342	6.07298	6.34156	5.5848	5.10404	5.66828	5.35826	4.89162	5.64496	5.9688	5.7832	5.90256	5.40329	5.62032	6.55351	5.58065	5.82137	5.73742	0.200663	0.273709	0.509922
Potassium	KCNJ2	3733275	5.55384	5.41147	6.19952	5.36742	7.61937	6.71006	7.53679	8.02807	6.77026	7.53433	7.57594	8.67725	5.25258	5.43859	5.79791	6.03325	6.0819	7.68515	6.05618	7.27488	6.60793	8.26601	7.84599	7.42457	-0.26828	0.302264	0.536533
Potassium	KCNJ16	3733238	4.41125	5.00511	6.02386	6.85858	6.60781	7.95467	7.68663	6.01855	6.36557	5.22795	6.10097	6.20717	4.28375	4.42081	7.25903	7.0483	8.13022	7.16805	6.76791	7.18488	7.4259	7.44872	5.37018	5.90074	0.328364	0.307889	0.542149
Potassium	KCNMA1	3296046	8.16462	7.88639	10.1635	9.10165	9.98164	9.4228	10.0542	10.3558	10.2507	9.96659	9.71639	10.2211	8.71962	8.849	9.79875	9.85936	9.9269	9.3097	8.80221	9.79315	8.76601	9.48748	9.76023	9.42501	-0.23233	0.30902	0.543199
Potassium	KCNJ6	3931495	8.60994	8.15914	8.52092	7.95777	8.6631	7.57383	8.21832	7.98189	8.01606	7.73394	7.69295	7.78514	8.42394	7.18535	9.1351	9.60393	8.58348	9.32131	7.74755	8.61696	7.2633	7.75599	7.96334	8.32052	0.250648	0.32839	0.560414
Potassium	KCNJ10	2439960	4.82944	5.71396	9.19146	8.92869	9.38302	11.0179	10.0989	10.0037	10.9005	9.97683	10.1344	10.081	5.94338	4.41504	8.01037	9.14225	9.35167	9.57627	9.93416	9.84441	11.0222	10.0033	9.39342	10.8207	-0.23355	0.330872	0.56253
Potassium	KCNJ8	3446910	4.78114	5.8071	5.98396	4.86585	5.73697	5.92432	7.24119	5.93599	6.42251	6.71096	5.49566	6.43143	6.30362	5.33661	5.28163	5.15085	6.03211	5.72343	6.35318	6.258	5.43898	5.34793	6.07279	4.98582	-0.25434	0.340584	0.572311
Potassium	KCNS1	3907151	3.97747	4.10733	4.94269	4.33896	4.72034	4.23722	5.24402	5.35415	6.66194	6.64252	6.37294	6.8646	4.22006	4.17889	4.81379	4.54927	4.47247	4.54933	5.57986	5.03894	6.69842	5.16788	6.24035	6.49951	-0.12128	0.409925	0.632506
Potassium	KCNK10	3575241	6.45021	6.83229	5.74287	5.52924	4.9334	5.51471	5.48553	5.81567	5.52377	5.82328	5.71848	5.58214	6.9019	6.44713	6.87793	5.74306	5.67042	5.40121	5.40482	5.42759	5.88242	5.63453	5.30505	5.61243	0.113075	0.435972	0.653493
Potassium	KCNK4	3334446	4.92739	5.17517	5.39327	5.80294	5.62049	4.90421	4.64118	5.30164	5.47755	5.84208	5.55778	5.34939	4.85817	5.1295	5.42706	5.32773	5.02876	5.1687	5.4564	5.14703	5.67733	5.37174	5.07566	5.20807	-0.09308	0.436261	0.653696
Potassium	KCNG2	3795403	5.11693	5.11815	5.41198	5.57113	6.12872	5.26668	6.41847	6.49114	5.22797	5.99993	5.52449	5.17395	5.44458	5.02023	5.18816	5.81707	5.38544	5.67069	6.12684	5.70912	5.7183	5.83047	5.25439	5.17604	-0.09235	0.455287	0.668631
Potassium	KCNA10	2427538	4.18057	4.58135	4.32283	4.9484	4.28118	4.17828	4.57776	4.01498	3.87894	4.19156	3.88905	4.3265	4.16911	4.3066	4.27097	4.24533	4.38783	3.69812	4.5912	4.0307	4.26	5.04622	4.30795	5.24753	0.09918	0.494165	0.698949
Potassium	KCND1	4007689	3.81142	4.33405	5.85199	4.90604	4.88687	5.17135	5.24294	5.16559	5.24344	5.57032	5.43819	4.96415	4.34695	4.46821	6.33418	5.19458	4.96156	5.29119	5.63989	4.91089	5.62315	4.88134	4.71627	5.0362	0.068172	0.586347	0.762894
Potassium	KCNH4	3757690	4.81882	5.27858	7.2426	6.66747	6.6492	5.51466	5.52924	5.74291	6.56447	5.53293	6.09705	5.34835	4.97008	5.15665	6.55018	6.98776	6.2139	5.58318	6.08996	6.28456	5.75296	5.45046	5.53644	5.56727	-0.07024	0.612026	0.781096
Potassium	KCNJ1	3397774	3.9399	3.9078	4.02794	4.18432	4.15172	4.12751	3.82735	4.32716	3.71964	4.02026	3.81346	4.13505	4.08936	4.05293	4.15716	4.02494	3.99654	3.95871	4.22961	3.89354	4.65682	3.92961	3.48534	4.35567	0.05401	0.62191	0.788138
Potassium	KCNK7	3377737	4.19255	4.18413	4.61584	4.43997	4.53127	4.80848	4.54831	4.89469	4.24982	4.62526	5.36013	4.58619	4.35918	4.51311	4.39638	4.6177	5.07343	4.84761	4.73819	4.48334	4.98408	4.40907	4.58267	4.75106	0.059932	0.626422	0.791546
Potassium	KCNK15	3886598	5.66432	5.91218	5.61889	5.7174	6.55541	5.28463	5.97666	5.91039	5.53602	5.94351	5.5197	5.46903	5.83705	5.70463	5.79281	5.96808	5.75388	6.23388	5.78394	5.95478	5.55807	5.60014	5.76579	5.8966	0.061793	0.63096	0.794561
Potassium	KCNH5	3567873	6.56128	7.67427	8.29534	7.68839	8.15988	7.69938	9.48657	8.50003	8.27797	7.91353	8.21216	8.33514	7.31053	7.20493	9.69289	8.59712	8.04014	8.65916	7.6094	8.68509	6.83443	7.03119	7.7448	7.88708	-0.1256	0.673933	0.821987
Potassium	KCNQ2	3913821	7.59951	7.41043	8.25072	8.24573	7.7253	8.77523	7.25349	7.95952	8.60134	8.22067	7.84397	7.56773	7.56828	7.67077	7.86513	8.11404	7.77408	8.89377	7.20637	8.25534	8.20624	7.661	7.66817	8.17205	-0.0332	0.73353	0.858283
Potassium	KCNG3	2550175	4.1097	5.02003	6.61288	7.0687	7.33079	6.29449	6.93273	6.65626	6.46312	6.73748	7.2661	7.31885	4.29642	4.73461	5.79428	7.31671	7.17836	7.27946	6.57494	7.08135	6.09383	8.17881	6.46878	7.37601	0.046869	0.813696	0.902686
Potassium	KCNA7	3867669	4.12122	4.40257	3.9156	4.18012	4.33418	4.14123	4.20237	4.49281	4.00303	4.19016	4.51882	4.31663	4.21697	4.28844	4.17716	4.31586	4.57628	4.29427	4.24327	4.08175	4.53213	3.65506	4.17374	4.5255	0.021808	0.814339	0.903016
Potassium	KCNU1	3093781	3.96221	4.48437	4.6849	4.52991	4.2731	4.60671	3.91104	3.75156	3.87086	4.84154	3.93906	4.45915	4.22278	4.11971	3.93317	4.02654	4.15154	4.37905	3.999	3.70277	4.79809	3.97698	4.75508	4.89961	-0.02917	0.862094	0.928747
Potassium	KCNH2	3079202	7.0702	6.84784	7.18179	6.45699	6.73137	7.08137	6.92838	7.16262	7.20784	7.50171	7.3332	6.78653	7.67895	8.02534	6.44851	6.76581	6.67236	6.89796	7.47763	7.05435	6.55498	6.45217	7.03694	6.84091	-0.03199	0.863257	0.929258
Potassium	KCNA5	3402039	6.22138	5.89131	5.14755	5.03491	4.43532	5.04307	5.1916	4.90997	4.69501	5.61114	5.45913	5.43319	5.16045	4.90621	5.08056	5.12618	5.49717	5.65863	5.06851	4.54688	5.66738	5.793	5.14151	5.10129	-0.02715	0.890955	0.944025
Potassium	KCNJ4	3960605	6.28048	6.97726	8.61798	9.18924	7.72485	9.45809	8.6207	8.59531	9.23999	8.85277	8.91156	8.89219	6.63295	7.22251	7.8479	8.78211	8.69217	9.23124	8.49853	8.87404	9.71185	8.08357	8.88954	8.91986	-0.00618	0.967167	0.984608
Potassium	KCNA4	3367580	5.744	5.72085	6.72697	7.0446	6.73948	7.17291	7.14566	6.84036	7.21521	6.93005	7.17905	7.44638	6.29289	6.12099	7.102	7.78871	7.02267	7.34167	6.29822	6.8171	6.96387	6.98333	6.75283	6.34564	-0.0063	0.969578	0.985883
Potassium	KCNA3	2427619	8.62049	7.85939	7.62314	6.24776	8.45337	6.1157	7.47918	6.66681	7.1789	7.59004	7.43666	6.99102	8.20759	8.17151	9.36025	7.94491	7.62916	6.89983	6.74768	7.5466	5.9951	6.57026	6.97641	6.23914	0.002165	0.994396	0.997521
Potassium	HCN1	2855963	9.35342	9.95411	10.6952	8.86805	9.7325	8.38923	11.3784	11.6025	10.8457	11.3541	10.8245	11.1711	9.69588	9.75675	10.0316	10.3722	9.46156	9.66324	10.6212	10.728	7.13209	9.84289	10.7625	9.54928	-0.54597	0.198612	0.435498
Potassium	HCN2	3815045	6.10539	6.10859	6.90443	6.80081	6.74483	6.96328	5.72053	7.2461	7.37921	7.40154	7.74246	6.79794	5.96435	5.97503	6.51705	6.6181	6.44128	6.81955	6.92065	7.2954	7.04154	6.96355	7.33906	7.13631	-0.0736	0.587868	0.763784
Potassium	HCN3	2360818	7.99023	8.39632	6.38717	6.29303	6.88625	6.47457	5.99824	6.82223	6.3067	6.54177	6.38187	6.77441	8.53851	8.07536	7.36543	7.65333	6.62805	7.13087	6.81579	5.69411	7.24314	7.06579	6.66287	6.83725	0.371476	0.088822	0.29732
Potassium	HCN4	3632424	7.19238	6.924	5.92973	5.13131	5.77943	4.66591	5.25882	5.645	5.31128	5.37936	5.89767	5.6886	6.8134	6.94898	6.49272	6.12994	5.63555	5.82766	5.83521	5.65354	5.61394	5.34715	5.20335	6.0058	0.225313	0.177448	0.41036
Sodium	SCN1A	2585322	5.09245	4.34262	7.22245	7.97553	8.14429	7.48974	9.05867	8.38514	8.91581	9.03052	8.71196	9.42909	4.58631	4.46886	7.43465	7.50653	7.95859	7.76599	7.43904	7.53261	6.69966	6.11241	8.62566	8.04418	-0.80198	0.020597	0.158883
Sodium	SCN2A	2513471	10.5228	10.6503	11.3036	11.3463	11.6448	10.7064	11.3588	11.0596	10.6798	10.8556	10.818	11.2585	10.4703	10.7531	11.8115	11.421	11.2172	10.7066	10.6072	10.7416	8.20967	10.5773	10.8178	10.1496	-0.39347	0.108007	0.324602
Sodium	SCN3A	2584957	10.3917	10.3573	8.96297	9.02547	9.44213	8.13385	8.6215	7.34056	7.54933	7.42681	6.72451	7.59467	9.79416	9.97771	10.4348	8.85393	8.58329	7.89077	7.43699	7.92156	7.05312	6.78069	7.66669	6.95718	-0.18499	0.431718	0.649536
Sodium	SCN4A	3766549	4.38249	4.37222	4.39594	4.94557	4.85396	4.56573	4.35467	4.70471	4.4002	4.5038	4.52845	4.44022	4.75017	4.53033	4.42415	4.71306	4.88178	4.75601	4.99946	4.71937	4.98446	4.66119	4.70653	4.98483	0.221948	0.014354	0.140714
Sodium	SCN5A	2669641	4.34245	5.18348	5.85487	5.62962	5.19862	5.14616	5.17836	5.10002	4.95621	4.68651	5.00701	4.96833	5.03811	5.20328	5.74858	5.33378	5.16902	5.44621	5.54665	5.58481	5.76652	5.0328	5.10281	5.50342	0.268696	0.018531	0.153765
Sodium	SCN8A	3414969	8.30856	7.68418	9.77903	8.19316	8.49161	8.80149	9.96461	9.9567	9.9028	9.91984	9.11127	9.86996	8.10741	7.95535	9.44835	9.97284	8.10599	8.92645	8.18823	8.97711	8.14702	8.76793	9.18254	8.6338	-0.46418	0.136579	0.362781
Sodium	SCN9A	2585400	8.38801	8.07937	6.11842	5.78658	5.60142	5.36795	5.15894	4.56253	4.49688	4.85259	4.51106	4.51174	7.0851	8.1886	9.00124	6.20812	6.18104	5.89314	5.40822	4.83281	5.84306	5.30188	4.83964	5.39252	0.561657	0.066699	0.262857
Sodium	SCN10A	2669732	3.71665	4.45259	4.16798	4.46467	4.18817	4.39718	4.0321	4.3077	3.90551	4.13684	4.40294	4.31557	4.02351	4.16365	3.89301	3.99864	4.311	4.52961	4.28139	4.19107	4.95046	4.22058	4.17448	4.84307	0.091048	0.464868	0.676284
Sodium	SCN11A	2669803	3.78279	4.19691	4.11764	4.01916	4.05607	4.2704	4.09979	4.24076	4.07304	4.098	4.32245	4.3907	4.07729	4.41507	3.83416	4.37045	4.24818	4.37581	4.59992	4.02466	5.12993	4.14915	4.17493	4.70438	0.203018	0.07688	0.280486
Sodium	SCN1B	3830051	4.64145	5.25027	7.24494	7.52404	6.9956	7.87476	7.52753	8.78336	9.61386	9.5001	9.18483	9.00659	5.94773	6.23397	6.14325	6.7974	7.46953	7.27454	8.44075	7.93349	8.52309	7.94254	8.95263	9.218	-0.1892	0.502735	0.705162
Sodium	SCN2B	3393652	4.98951	4.96797	8.09231	7.49153	9.10493	7.48493	8.13989	9.12652	9.07657	9.02433	9.80972	8.54692	4.88082	5.27161	7.05086	8.17448	8.54269	8.70765	7.72232	8.89647	6.73839	8.61786	9.16426	8.79084	-0.27474	0.312606	0.54619
Sodium	SCN3B	3395691	10.9775	10.4077	11.4502	10.4856	11.3461	10.2333	11.3363	11.1744	10.6977	10.9056	10.7745	10.6679	10.4305	10.5432	11.2048	11.0493	10.0693	10.458	10.669	11.105	8.04298	10.5616	10.2061	9.20152	-0.57629	0.044024	0.222553
Sodium	SCN4B	3393622	6.35224	6.13634	7.22367	7.20383	7.09261	6.69986	7.50278	7.58036	8.48693	8.26724	7.94237	7.86816	5.81188	5.77145	6.93853	7.30083	6.89573	6.69555	7.50358	7.99323	7.46216	7.34274	7.92963	8.41324	-0.19149	0.190548	0.426437
Glutamate	GRM1	2929571	6.9646	7.66599	7.88528	6.06117	6.91248	5.92181	6.96374	6.58456	6.17401	6.46173	6.2542	6.82959	6.31541	6.15232	8.00567	7.15371	6.63151	6.51451	7.01186	7.02115	5.8387	6.6571	6.58342	6.04399	-0.06248	0.762578	0.874728
Glutamate	GRM2	2623308	7.78965	7.46343	7.30547	6.8592	7.46776	7.09161	7.82568	8.21332	6.81237	7.5979	7.44641	7.03506	8.14434	7.97804	7.1066	7.65768	5.47051	7.12179	6.88763	7.32461	5.3747	6.0755	6.45724	5.47796	-0.65261	0.032535	0.193731
Glutamate	GRM3	3011180	9.74173	10.1592	9.9968	10.1394	10.1856	11.2244	10.5459	10.2972	10.3916	10.0238	9.78729	10.1139	10.2006	10.2335	10.1702	9.79147	10.4848	10.2267	9.98797	9.86444	9.39486	9.75358	9.33279	9.38808	-0.31482	0.045087	0.224119
Glutamate	GRM4	2950970	6.54535	6.50557	6.94835	5.9122	6.51156	6.07749	6.16747	6.90883	6.04156	6.61541	7.14051	6.68577	6.82759	6.5307	6.62747	7.10177	6.47441	6.77802	6.89038	6.51252	7.04858	6.47718	6.39521	6.76149	0.197104	0.275738	0.51165
Glutamate	GRM5	3385834	10.0207	9.84068	10.726	8.85442	9.54661	9.14863	10.4384	9.77156	9.8191	9.70718	9.68514	10.0075	9.7842	9.51233	10.9225	10.4948	9.99087	9.77466	9.16089	9.85892	8.80281	9.35967	9.91232	9.78563	-0.01719	0.939036	0.96939
Glutamate	GRM6	2889848	4.30041	4.85992	4.38101	4.59965	4.46797	4.39557	4.39672	4.51018	4.12381	4.78546	4.49877	4.4059	4.33079	4.90699	4.31605	4.60515	4.28005	4.7664	5.11363	4.17771	4.89822	4.50723	4.51261	4.99747	0.140578	0.227246	0.46511
Glutamate	GRM7	2609061	9.84873	9.67059	8.89439	8.8615	8.23802	7.96431	8.2186	7.24969	7.6388	7.46705	7.68894	6.78426	7.42308	8.63918	9.6062	8.86337	8.49004	7.86963	7.0413	7.80061	6.61144	6.85507	6.24304	6.36922	-0.55939	0.057153	0.246761
Glutamate	GRM8	3071063	3.96692	4.30785	5.53899	6.11841	5.45035	5.22925	6.00485	6.06567	5.92758	6.16355	5.90578	5.90832	4.16212	4.99274	5.08245	5.86418	5.13825	6.08603	4.84952	5.35511	4.61438	5.17809	5.38301	5.42743	-0.37118	0.082889	0.288985
Glutamate	GRIK1	3928211	4.31625	4.64284	8.08866	8.2507	9.07786	6.97122	7.53415	7.66934	7.41725	7.49234	7.58118	7.21589	5.51478	4.71849	7.47732	9.36772	9.26277	7.88216	8.16258	8.78608	7.1632	7.3485	7.0451	6.99354	0.288713	0.165706	0.39739
Glutamate	GRIK2	2918982	10.1621	9.61731	8.7164	9.94105	9.44504	9.07282	8.27331	8.56561	9.02951	8.71417	8.28433	8.58209	9.11004	8.43402	9.53687	9.30805	9.53007	8.78975	8.71052	8.55479	7.82872	8.68958	8.32998	8.54137	-0.25357	0.198872	0.435579
Glutamate	GRIK3	2406926	10.1052	9.15836	9.97605	8.40295	7.96809	8.49264	8.67691	8.67644	8.65685	9.00167	8.81143	8.08814	10.1411	9.42466	8.97416	8.15813	8.19844	8.14684	7.22883	7.67089	6.76241	6.76452	7.45928	6.50194	-0.88196	0.004334	0.096319
Glutamate	GRIK4	3352618	7.27962	7.12795	8.43943	7.80409	8.16993	8.44398	8.82329	7.98322	8.23127	7.97187	8.18898	8.36823	6.13846	6.35946	7.83931	7.56769	8.27987	8.23591	7.82709	7.78749	6.65364	8.3401	7.58998	7.76225	-0.53755	0.005813	0.107475
Glutamate	GRIK5	3863380	9.11596	8.51005	9.25841	8.51467	8.22999	9.15891	7.92031	8.00354	8.72673	7.90862	8.06568	7.63386	8.90341	8.83718	8.98477	8.55566	8.00198	8.4132	7.5379	8.35606	8.11802	8.33519	8.04697	8.10729	-0.07076	0.559441	0.74432
Glutamate	GRIA1	2836242	11.0211	10.8211	10.6809	10.3538	10.6996	9.35001	9.94894	10.3305	9.95932	10.097	9.82156	9.74576	10.2026	10.4579	11.2085	10.8007	10.8006	9.97197	9.34459	9.9673	7.02357	9.16141	9.44774	8.84882	-0.46616	0.118504	0.338724
Glutamate	GRIA2	2749222	10.9234	10.2847	11.2441	11.8872	11.378	11.352	11.3981	11.1646	11.1604	11.1467	11.0234	11.1356	11.1458	10.8499	11.2804	11.4459	11.6987	11.3425	10.4819	10.9909	9.94699	10.7385	10.9067	10.6878	-0.21518	0.170023	0.401546
Glutamate	GRIA3	3989448	9.56881	9.82365	10.1694	9.14236	9.84165	9.24381	9.81427	10.9293	10.1323	9.88356	9.5027	9.84392	10.5841	9.59795	10.4513	9.65947	9.19341	9.26615	8.70077	9.64087	7.73303	9.16492	9.68172	9.25148	-0.41421	0.14784	0.376779
Glutamate	GRIA4	3347118	7.48708	7.01593	8.7413	8.78399	9.06976	7.64798	8.69252	8.65511	8.44519	7.94484	7.69603	8.19854	7.02921	7.06859	8.80103	9.27678	8.51563	7.83438	7.93177	8.11026	6.39428	7.82935	7.22171	7.35107	-0.41785	0.049723	0.232894
Glutamate	GRIN1	3195238	7.33534	7.40386	8.53439	7.89631	7.79487	7.47043	7.86017	8.25287	8.6011	8.43387	8.46537	7.68949	7.05255	7.14304	8.10018	7.91445	7.69116	7.71437	7.29537	8.02881	7.94499	7.62215	8.02448	7.5334	-0.30609	0.004251	0.095684
Glutamate	GRIN2A	3679812	6.10714	6.77851	10.3608	9.36816	9.13758	9.69644	10.5161	9.75684	9.42188	9.723	8.63459	9.76603	4.92043	5.93043	8.92859	9.70293	8.46645	9.14424	8.69894	9.20708	8.11799	8.37273	8.64369	8.54121	-0.8827	0.000489	0.049618
Glutamate	GRIN2B	3445326	11.0904	11.4254	11.4836	9.66821	11.0694	10.205	10.9001	11.3196	10.1423	10.2118	9.53379	10.7597	11.7454	11.5557	11.6166	10.9755	9.84527	10.1913	8.36598	10.2654	7.6352	8.42587	9.2817	9.14249	-0.73024	0.066416	0.262333
Glutamate	GRIN2C	3770422	5.28043	5.20397	6.03956	5.82734	6.65948	7.03079	5.78644	6.11076	7.01796	5.84011	6.52767	6.09869	5.30061	5.31528	5.80155	5.98512	6.01609	6.28033	6.06948	6.84161	6.79688	6.1762	6.50968	6.57663	0.020522	0.87284	0.934076
Glutamate	GRIN2D	3837759	5.74182	6.009	5.66749	5.42231	5.59601	5.2319	5.22584	5.69406	5.52036	5.76351	5.4437	5.53933	5.92869	5.94012	6.44239	5.78772	5.58031	5.78348	6.00137	5.58462	5.80856	5.6282	5.80355	6.29156	0.310437	0.010276	0.127165
Glutamate	GRIN3A	3218151	5.88717	5.21065	9.35098	8.45349	7.51908	7.41287	7.8673	7.18527	7.14011	6.82792	6.75276	5.95	5.60416	5.3358	9.94109	9.80521	8.88631	8.52325	7.18901	7.44969	5.72729	6.94437	5.69899	7.18136	0.227411	0.42441	0.643913
Glutamate	GRID1	3298557	7.53386	7.47144	7.99477	7.52231	6.5954	7.91308	7.65355	7.72807	7.61686	7.92873	7.54701	7.90629	7.8604	7.37144	8.01985	7.57939	7.28884	7.35694	6.48053	6.9917	6.99688	6.86205	7.14014	7.54162	-0.3268	0.065438	0.261523
Glutamate	GRID2	2736060	7.99446	8.31885	8.14918	8.74224	7.58931	7.884	7.78355	7.25176	6.85949	7.29675	6.56081	6.61633	7.68551	6.54832	8.45185	7.96404	8.86142	7.6525	7.47567	7.19915	6.23597	7.83941	6.46046	7.01234	-0.13834	0.540068	0.730675
Glutamate	SYP	4007899	9.05091	8.23396	11.5858	11.2078	11.8251	11.4516	10.3238	11.6055	11.3452	11.5414	11.9851	10.5678	8.24211	8.55675	11.0838	11.8957	11.9674	12.012	11.3855	11.2921	11.6839	11.399	10.8795	12.0837	0.146458	0.52098	0.718941
Glutamate	SYN1	4007126	8.82551	9.31361	11.0638	10.6514	9.95353	9.99878	10.4523	10.6464	10.83	11.0132	10.7208	10.4692	8.45257	8.6494	10.1122	10.3523	9.69248	10.2778	10.5806	10.6166	8.721	11.0812	10.539	10.4761	-0.36561	0.076567	0.279942
Glutamate	DLG4	3743393	9.83209	10.0544	11.1888	10.6702	11.3336	10.9283	10.1639	11.5077	10.7324	10.9642	11.0162	10.5422	9.8524	9.66243	10.3498	10.6474	10.2864	10.6969	11.2084	11.0234	10.0988	10.9105	10.5785	10.2345	-0.28205	0.091433	0.300729
Glutamate	NLGN1	2652801	9.91758	9.80807	10.0697	8.70537	10.0502	8.1761	10.55	9.6438	8.73988	9.16096	8.31006	9.74758	11.1957	10.0118	10.4631	9.81776	9.88244	8.52626	9.00654	9.20337	6.94752	8.11668	8.28663	7.46698	-0.32954	0.33326	0.565046
Glutamate	SLC17A7	3867842	7.69406	8.51846	12.3159	11.8199	12.3213	12.0996	11.9806	12.3116	12.303	12.2752	11.7531	12.0439	8.40037	8.03974	11.0339	11.813	11.752	12.0008	11.8462	11.9719	11.6003	11.9919	11.7127	11.3328	-0.32842	0.039978	0.212425
Glutamate	SHANK1	3868587	7.65944	7.84441	9.59671	9.09994	9.21904	10.0467	8.60386	9.32814	9.53698	8.75027	8.865	8.5757	7.42654	7.95086	8.53623	9.41365	8.50856	9.54224	8.37642	9.42078	9.06288	9.17782	9.04077	8.7346	-0.16124	0.243541	0.482492
Glutamate	SHANK2	3380365	8.35048	7.95664	9.65794	8.56271	8.84246	9.14096	8.60743	8.34782	8.84867	8.32895	8.82146	7.71966	8.20242	7.75536	9.24505	8.8448	9.13434	8.879	7.48901	8.54022	8.00916	7.81353	7.81235	8.15686	-0.27526	0.096321	0.307035
